# IFI6 depletion inhibits esophageal squamous cell carcinoma progression through reactive oxygen species accumulation via mitochondrial dysfunction and endoplasmic reticulum stress

**DOI:** 10.1186/s13046-020-01646-3

**Published:** 2020-07-29

**Authors:** Zhenchuan Liu, Shaorui Gu, Tiancheng Lu, Kaiqing Wu, Lei Li, Chenglai Dong, Yongxin Zhou

**Affiliations:** Department of Thoracic Surgery, Shanghai Tongji Hospital Affiliated with Tongji University, Shanghai, 200065 P. R. China

**Keywords:** Interferon alpha inducible protein 6, Esophageal squamous cell carcinoma, Reactive oxygen species, Mitochondrial oxidative phosphorylation, Endoplasmic reticulum stress

## Abstract

**Background:**

Esophageal squamous cell carcinoma (ESCC) is one of the most lethal forms of adult cancer with poor prognosis. Substantial evidence indicates that reactive oxygen species (ROS) are important modulators of aggressive cancer behavior. However, the mechanism by which ESCC cells integrate redox signals to modulate carcinoma progression remains elusive.

**Methods:**

The expression of interferon alpha inducible protein 6 (IFI6) in clinical ESCC tissues and cell lines was detected by RT-PCR and Western blotting. The correlation between IFI6 expression levels and aggressive ESCC disease stage was examined by immunohistochemistry. Bioinformatic analysis was conducted to explore the potential function of IFI6 in ESCC. ESCC cell lines stably depleted of IFI6 and ectopically expressing IFI6 were established using lentiviruses expressing shRNAs and an IFI6 expression plasmid, respectively. The effects of IFI6 on ESCC cells were determined by cell-based analyses, including EdU assay, apoptotic assay, cellular and mitochondria-specific ROS detection, seahorse extracellular flux, and mitochondrial calcium flux assays. Blue native-polyacrylamide gel electrophoresis was used to determine mitochondrial supercomplex assembly. Transcriptional activation of NADPH oxidase 4 (NOX4) via ATF3 was confirmed by dual luciferase assay. In vivo tumor growth was determined in mouse xenograft models.

**Results:**

We find that the expression of IFI6, an IFN-stimulated gene localized in the inner mitochondrial membrane, is markedly elevated in ESCC patients and a panel of ESCC cell lines. High IFI6 expression correlates with aggressive disease phenotype and poor prognosis in ESCC patients. IFI6 depletion suppresses proliferation and induces apoptosis by increasing ROS accumulation. Mechanistically, IFI6 ablation induces mitochondrial calcium overload by activating mitochondrial Ca^2+^ uniporter and subsequently ROS production. Following IFI6 ablation, mitochondrial ROS accumulation is also induced by mitochondrial supercomplex assembly suppression and oxidative phosphorylation dysfunction, while IFI6 overexpression produces the opposite effects. Furthermore, energy starvation induced by IFI6 inhibition drives endoplasmic reticulum stress through disrupting endoplasmic reticulum calcium uptake, which upregulates NOX4-derived ROS production in an ATF3-dependent manner. Finally, the results in xenograft models of ESCC further corroborate the in vitro findings.

**Conclusion:**

Our study unveils a novel redox homeostasis signaling pathway that regulates ESCC pathobiology and identifies IFI6 as a potential druggable target in ESCC.

## Background

Esophageal carcinoma is one of the most lethal adult digestive tract tumors with serious malignant characteristics in terms of both mortality and prognosis [1]. In China, esophageal squamous cell carcinoma (ESCC) is the predominant histological subtype of esophageal carcinoma [2]. The current standard of care for ESCC management consists of complete surgical resection followed by chemotherapy as well as radiation [3, 4]. Although enormous advancement has been made in diagnostic technologies and comprehensive management approaches for ESCC, the overall 5-year survival rate for ESCC remains unsatisfactory [5]. Currently, the specific mechanism underlying ESCC onset and progression remains to be further defined. Above all, identifying the molecular mechanisms involved in ESCC development and progression, ultimately facilitating the development of more effective treatment strategies against ESCC, is crucial.

Emerging evidence indicates that oxidative stress, which occurs in response to excessive reactive oxygen species (ROS) accumulation and dysregulated cellular redox dynamics, plays diverse and important roles in modulating various aspects of cell behavior, ranging from inducing oxidative damage and subsequent cell death to modulating cell proliferation and survival [6]. Not surprisingly, therefore, the control of ROS production has been associated with many aspects of carcinogenesis, metabolic reprogramming, aggressive cancer phenotypes, and drug resistance development [7]. Numerous lines of evidence support the idea that the role of ROS in transformed cells is highly complex and somewhat controversial. For instance, mitochondria-derived ROS are necessary for Kras-mediated tumorigenicity [8]. In contrast, ROS can enhance chronic inflammation and induce genotoxic damage in cancer cells [9, 10]. However, subsequent studies support the idea that although moderately increased oxidative stress is essential for the initiation and progression of carcinoma, cancer cells inherently exhibit a high ROS burden in response to aberrant oncogenic pathways and microenvironments. Multiple concomitant, highly efficient antioxidant mechanisms that are not necessarily needed in normal cells must exist to ensure the survival of these cancer cells. Thus, specifically inhibiting these ROS scavenging pathways or increasing the ROS burden may selectively kill tumor cells [11–13].

As so-called cell powerhouses, mitochondria comprise a large percentage of the cell mass and are central to cellular bioenergetics and metabolism. As studies have shown, the Warburg effect, or the shift to aerobic glycolysis, has been assumed to be a prototypical characteristic of tumor cells, reflecting an elevated metabolic demand, rapid growth, and the use of a more efficient energy source [14, 15]. However, recent studies provide evidence that mitochondrial oxidative phosphorylation (OXPHOS) also plays an indispensable role in carcinogenesis [16, 17]. As a byproduct of oxidative metabolism, ROS are released through the electron transport chain (ETC), and aberrant ROS production consequently profoundly influences tumor initiation and progression, as stated previously.

As a player in the redox balance, the endoplasmic reticulum (ER) has a well-established role in protein synthesis/folding, which is highly sensitive to cellular redox dynamics, and dysregulation of disulfide bond formation due to endoplasmic stress ultimately induces ER oxidative stress and aberrant ER function [18]. In addition, the ER contains a set of ROS scavengers that are indispensable for proper ER function [19]. Moreover, because of their precise contact sites and close proximity to one another, functional changes in the ER or mitochondria can influence the redox balance in the other organelle [20].

Recent findings demonstrated that interferon alpha inducible protein 6 (IFI6), an interferon (IFN)-stimulated gene (ISG), is enriched mainly in the inner mitochondrial membrane and implicated in diverse malignant diseases, including myeloma and breast and gastric cancers [21–23]. Although the understanding of its biological functions is limited, IFI6 was characterized as a proliferative and anti-apoptotic factor [21, 23]. Furthermore, IFI6 was suggested to facilitate breast cancer metastasis by modulating mitochondrial ROS production [24]. However, both the biological role of IFI6 and the mechanism underlying IFI6-mediated effects in ESCC are unknown.

In this study, we first examined the abundance of IFI6 in ESCC tissues. We showed that IFI6 contributed to ESCC cell proliferation and survival by modulating redox homeostasis. We further established that IFI6 downregulation led to mitochondrial Ca^2+^ uniporter (MCU) mediated mitochondrial calcium overload, and inhibited mitochondrial supercomplex assembly and suppressed the respiratory phosphorylation efficiency, which ultimately elevated mitochondrial ROS production. Finally, we found that IFI6 inhibition induced ATP deprivation-mediated ER stress, which in turn elevated NOX4 expression in an ATF3-dependent manner and enhanced ROS production. Therefore, disrupting IFI6 is a promising therapeutic strategy for ESCC.

## Materials and methods

### Reagents and antibodies

Lipofectamine 3000 (L3000015), MitoSOX (M36008), carboxy-H_2_DCFDA (C400) and the anti-IFI6 antibody (1:1000, PA5–99824) were purchased from Invitrogen (CA, USA). RPMI-1640 (72400047), penicillin and streptomycin (10378016), and Fetal bovine serum (FBS) (16140071) were obtained from Gibco (CA, USA). N-acetyl-L-cysteine (NAC) (A7250), PEG-catalase (CAT) (C1345), dithiothreitol (DDT) (45839), MitoTEMPO (SML0737), ATP (A6559), BAPTA-AM (A4926), Phenformin (PHE) (P7045), Cyclopiazonic acid (CPA) (C1530), Tunicamycin (Tunica) (654380) and Thapsigargin (Tg) (T9033) were purchased from Sigma-Aldrich (Munich, Germany). DS16570511 (HY-115595) was obtained from MedChemExpress (NJ, USA). Antibodies against GAPDH (1:10000, ab181602), β-Actin (1:5000, ab179467), NCLX (1:1000, ab136975), MCU (1:1000, ab121499), VDAC1 (1 μg/ml, ab15895), NDUFB8 (1:5000, ab192878), SDHA (1:5000, ab137040), RISP (1 μg/ml, ab14746), COXIV (1 μg/ml, ab33985), ATPB (1:10000, ab170947), 4-HNE (1:1000, ab48506), MDA (1:1000, ab243066), ATF3 (1:2000, ab207434), ATF4 (1:1000, ab184909), ATF6 (1:1000, ab122897), XBP1 (1:5000, ab109221), PDI (1:1000, ab2792), BiP (1:1000, ab21685), NOX1 (0.5 μg/ml, ab131088), NOX2 (1 μg/ml, ab80508), NOX3 (1 μg/ml, ab81864), NOX4 (1:1000, ab133303), and NOX5 (1:1000, ab198213) were obtained from Abcam (Cambridge, UK).

### Human study subjects and cell cultures

A total of 23 fresh ESCC samples and paired adjacent non-tumorous tissues (PANTs) were collected from patients undergoing esophagectomy at Shanghai Tongji Hospital Affiliated with Tongji University. Immunohistochemistry (IHC) for IFI6 was conducted on 3-μm sections of formalin-fixed, paraffin-embedded tissue samples consisting of nonpathological esophageal tissues (8 specimens), esophageal hyperplasia tissues (12 specimens) and ESCC tissues (83 specimens). The clinicopathological characteristics of the subjects are summarized in Tables S1 and S3. Informed consent was obtained from each patient before enrollment in this study. This study was approved by the Medical Ethics Committee of Shanghai Tongji Hospital.

The human ESCC cell lines Eca109, TE-1, Ec9706, Kyse150, and Kyse410 and the normal esophageal squamous epithelial cell line Het-1a were obtained from the Cell Bank of the Chinese Academy of Sciences (Shanghai, China). All cells were cultivated in RPMI 1640 medium supplemented with 10% FBS and 1% penicillin and streptomycin at 37 °C in 5% CO_2_. All cell lines were authenticated by the STR method and tested for mycoplasma contamination.

### In vivo study

Four- to five-week-old female BALB/c nude mice were purchased from Jiesijie Laboratory Animal (Shanghai, China). All animal experiments were approved by the Animal Care and Use Committee of Shanghai Tongji Hospital and conducted in accordance with ethical standards. Nude mice were randomly divided into four groups as follows (*n* = 5 mice/group): (i) ShControl; (ii) IFI6 KD; (iii) OEControl; and (iv) IFI6 OE.

For in vivo model exploring the effect of MCU inhibition on tumor growth, nude mice were randomly divided into three groups as follows (*n* = 5 mice/group): (i) ShControl; (ii) IFI6 KD; (iii) IFI6 KD + DS16570511 (DS16570511 (1 mg/kg) was administered to mice intraperitoneally every week for four weeks).

For in vivo model exploring the effect of OXPHOS inhibition on tumor growth, nude mice were randomly divided into three groups as follows (*n* = 5 mice/group): (i) OEControl; (ii) IFI6 OE; (iii) IFI6 OE+ Phenformin (Phenformin (500 mg/kg) was administered to mice intraperitoneally every other day for four weeks).

The indicated Eca109 cells (2 × 10^6^ cells per 0.1 ml of phosphate-buffered saline) were suspended in Matrigel (Biosciences, CA, USA) and implanted subcutaneously into the right flanks of the nude mice. The tumor volume was measured using a caliper every 5 days. Four weeks after implantation, the nude mice were sacrificed, and tumor samples were harvested.

### RNA extraction and qRT-PCR

One microgram of total RNA was extracted using TRIzol obtained from Invitrogen (CA, USA) and was reverse transcribed to cDNA with SuperScript II (Invitrogen, CA, USA). qRT-PCR was conducted using SYBR Green technology (Takara Bio, Beijing, China). β-Actin was used as the housekeeping gene. Data were analyzed through the 2^-ΔCT^ method. The primers used for detection are shown in Table S4.

### Immunoblotting

Cells and tissues were collected and lysed in cell/tissue lysis buffer (Solarbio, Beijing, China). Proteins were extracted, and the protein concentration was measured by the BCA method (Solarbio, Beijing, China). Proteins were separated by 10% sodium dodecyl sulfate (SDS)-PAGE and transferred to 0.45-μm polyvinylidene difluoride membranes (Invitrogen, CA, USA), which were blocked with blocking buffer (EpiZyme, Shanghai, China), incubated overnight with primary antibodies, and washed and incubated with the HRP-conjugated anti-rabbit secondary antibody (Abcam, UK) for 1 h. Peroxidase activity was visualized via the ECL method (EpiZyme, Shanghai, China).

### Immunohistochemistry

Paraffin-embedded sections were deparaffinized and rehydrated and were then subjected to antigen retrieval for 20 min in citrate buffer (pH 6.0). After the sections were cooled, 3% H_2_O_2_ was used to block endogenous peroxidases. The sections were blocked with 10% goat serum and incubated with the anti-IFI6 primary antibody (Invitrogen, Carlsbad, CA, USA) at a 1:1000 dilution overnight at 4 °C. After incubation with the secondary antibody, immunodetection was performed with DAB staining (Zhongshan Goldenbridge Biotechnology Company, Beijing, China). For evaluation, three staining fields in each section were randomly selected and evaluated by two independent pathologists who were blinded to patient information. The (Staining index) SI of IFI6 was determined by combining the staining intensity score (1, negative; 2, weak staining; 3, moderate staining; 4, strong staining) and the proportion of positively stained tumor cells (0, no positive cells; 1, < 10%; 2, 10–35%; 3, 35–75%; 4, > 75%). Samples with SI ≥ 8 were considered to exhibit high expression, samples with SI 3–7 were regarded as medium expression, while samples with SI < 3 were deemed to be low expression samples.

### BN-page

Mitochondria were isolated from cells with a mitochondria extraction kit (Solarbio, Beijing, China) according to the manufacturer’s instructions. BN-PAGE was performed with the NativePAGE™ system (Invitrogen, CA, USA). In brief, mitochondria were solubilized with digitonin (digitonin/protein ratio of 4 g g^− 1^), incubated on ice for 30 min, and centrifuged at 20000×g for 30 min. The solubilized protein was collected from the supernatant, and the protein concentration was determined by the BCA (Solarbio, Beijing, China) method. Protein samples were mixed with Coomassie blue G-250 (Invitrogen, CA, USA) to obtain a dye/detergent mass ratio of 1/4 and were then loaded into a 4–16% Bis-Tris gel. After electrophoresis, proteins were transferred to 0.45-μm polyvinylidene difluoride membranes (Invitrogen, CA, USA) and were then sequentially probed with antibodies against complex I (NDUFB8), complex III (RISP), complex IV (COX IV), complex II (SDHA) and complex V (ATPB). The NDUFB8, RISP and COX IV immunoblot bands with high molecular weights were used to identify the complex I-, complex III-, or complex IV-containing respiratory supercomplexes (RSCs). Signal intensities were normalized to their corresponding ATPB signals.

### Cell proliferation assay

To assess cell proliferation, the indicated Eca109 and TE-1 cells were seeded in 96-well plates and incubated under standard conditions in complete medium. Twenty-four hours after seeding, cells were incubated with 50 μM EdU (RiboBio, Guangzhou, China) for 6 h and subsequently subjected to fixation, permeabilization and EdU staining, which were conducted according to the manufacturer’s instructions. Nuclei were counterstained with Hoechst 33342 (Invitrogen, CA, USA) for 10 min. The proportion of cells stained with EdU was determined via fluorescence microscopy.

### Detection of the mitochondrial membrane potential and apoptosis induction

The indicated Eca109 and TE-1 cells were seeded in 6-well plates and treated as indicated. Then, the cells were detached, washed, double-stained with Annexin V-FITC and PI (BD Biosciences, CA, USA) based on the manufacturer’s recommendation, and analyzed by flow cytometry (Beckman Coulter, CA, USA). FlowJo (Tree Star, OR, USA) was used for data presentation and analysis.

The mitochondrial membrane potential was assessed via the JC-1 method. In brief, the indicated cells were washed and incubated with JC-1 (Beyotime, Beijing, China) working solutions for 20 min at 37 °C in the dark. After incubation, the cells were washed with JC-1 wash buffer and observed under a fluorescence microscope. In healthy cells with a Δψm indicating membrane depolarization, JC-1 forms JC-1 aggregates exhibiting red fluorescence. In apoptotic cells, JC-1 remains monomeric, exhibiting green fluorescence.

### Measurement of ATP levels

To assess cellular ATP production, an ATP luminescence assay (Invitrogen, CA, USA) was used for quantitative determination of ATP levels with recombinant firefly luciferase and its substrate D-luciferin according to the manufacturer’s recommendation. After a standard curve for a series of ATP concentrations was generated, the ATP content in the samples was calculated from the standard curve.

### Analysis of cellular respiration

The Seahorse XFe96 Bioanalyzer (Seahorse Bioscience, MA, USA) was used to determine the OCR and ECAR according to the manufacturer’s instructions. ECAR and OCR were determined using the Seahorse XF Glycolysis Stress Test Kit (Seahorse Bioscience, MA, USA) and the Seahorse XF Cell Mito Stress Test Kit (Seahorse Bioscience, MA, USA), respectively. In brief, for OCR determination, 20,000 of the indicated ESCC cells were seeded in a Seahorse 96-well plate, and 1 h before measurement, the culture medium was replaced with Seahorse XF medium supplemented with 1 mM sodium pyruvate, 2 mM glutamate, and 25 mM glucose. Periodic measurements of the OCR were performed at baseline and after sequential administration of oligomycin (Oligo, 1 μM), trifluoromethoxy carbonylcyanide phenylhydrazone (FCCP, 0.5 μM), and rotenone (Rot, 0.5 μM) and antimycin A (AMA, 0.5 μM) (Rot&AMA). For ECAR measurement, the culture medium was replaced with XF assay medium supplemented with L-glutamine (300 μg/ml). Glucose (10 mM), Oligo (1 μM) and 2-deoxy-D-glucose (2-DG) (100 mM) were sequentially added to the cell culture, and real-time ECAR was determined with the Seahorse XFe96 Bioanalyzer. For complex-specific OCR measurement, cells were permeabilized with a solution of digitonin (500 μg per 10^5^ cells) supplemented with 1 mM MgCl_2_, 1 mM ADP, 5 mM KH_2_PO_4_, 150 mM KCl and 5 mM Tris (pH 7.2). The substrates and inhibitors used for complex-specific OCR measurement were malate (5 mM), Rot (1 μM), AMA (1 μM), ascorbate (5 mM), TMPD (500 μM), pyruvate (5 mM), succinate (5 mM), and G3P (5 mM). All reagents and compounds were purchased from Sigma-Aldrich (Munich, Germany).

### Cell transfection

For lentiviral-mediated transduction of shRNA, cells were initially treated with polybrene (Sigma-Aldrich, Munich, Germany) and selected with puromycin (Sigma-Aldrich, Munich, Germany). ShRNA-IFI6, shRNA-ATF3, shRNA-NOX4, and negative control shRNAs were purchased from Genechem (Shanghai, China). The oligonucleotide sequences used to silence target genes are shown in Supplementary Table S5. IFI6, ATF3, and NOX4 lentiviruses were purchased from Genechem (Shanghai, China).

For construction of overexpression plasmids, the pcDNA3.1 (V79020) expression plasmid was obtained from Invitrogen (CA, USA). Human IFI6, ATF3, and NOX4 cDNA from Eca109 cells were cloned by PCR and subcloned into the pcDNA3.1 expression vector to generate IFI6-pcDNA3.1, ATF3-pcDNA3.1, and NOX4-pcDNA3.1. After construction, the IFI6-, ATF3-, or NOX4-pcDNA3.1 plasmid was transfected into Eca109 and TE-1 cells using Lipofectamine 3000 and selected with G418.

### Measurement of Ca^2+^

To monitor mitochondrial calcium dynamics, the indicated ESCC cells were incubated with the calcium-specific fluorescent probe Rhod-2 AM (Invitrogen, CA, USA) in Hanks’ balanced salt solution at room temperature for 30 min. After washout, fluorescence was recorded with an integrated spectrofluorometer (Photon Technology International, NJ, USA) at excitation and emission wavelengths of 550 nm and 580 nm, respectively. After 1 min of baseline recording (basal signal, F_0_), cells were promptly treated with the agonist Tg, and fluorescence intensities were monitored at 3-s intervals for another 10 min. The relative fluorescence intensity (F/F_0_), where F represents the fluorescence intensity at a given time and F_0_ represents the initial fluorescence intensity, was used to indicate [Ca^2+^].

For determination of Ca^2+^ concentration in endoplasmic reticulum, Eca109 and TE-1 cells were transfected with low affinity aequorin construct targeted to the ER (erAEQ) [25]. Cells expressing erAEQ were reconstituted in its active form with 1 μM coelenterazine by incubation for 1 h at room temperature in Ca^2+^ − free medium containing 0.5 mM EGTA. Cells were washed with Krebs Ringer Buffer (KRB: 125 mM NaCl, 5 mM KCl, 400 mM KH_2_PO_4_, 1 mM MgSO_4_, 20 mM HEPES, pH 7.4) supplemented with 2% bovine serum albumin (Sigma) and 1 mM EGTA and were then placed in KRB supplemented with 5 mM glucose and 75 mM EGTA and luminescence measurement was started. After 60 s, 1 mM CaCl_2_ at the final concentration was injected to refill the ER lumen. The measurement was carried out with an integrated spectrofluorometer (Photon Technology International, NJ, USA) 3-s intervals for 800 s.

### Detection of cellular and mitochondria-specific ROS determination

To detect mitochondrial ROS, the indicated ESCC cells were seeded in 24-well plates and incubated with the mitochondria-specific ROS probe MitoSOX (Invitrogen, CA, USA) for 20 min at 37 °C. For cellular ROS determination, the indicated ESCC cells were seeded in 24-well plates, loaded with the cellular ROS indicator carboxy-H_2_DCFDA and further incubated at 37 °C for 20 min. Nuclei were counterstained with Hoechst 33342, and the fluorescence intensity was measured by fluorescence microscopy or flow cytometry.

### Construction of reporter plasmids and luciferase assay

Fragments of the human ATF3, PDI and XBP1s were synthesized and subcloned into the pGL3 vector (Promega, WI, USA). Furthermore, different fragments (containing bp − 3000 to + 1, − 2000 to + 1, or − 1000 to + 1) of the human NOX4 promoter were synthesized and subcloned into the pGL3 vector (Promega, WI, USA). Another set of mutant plasmids was constructed; in these plasmids, one of the potential ATF3-binding motifs (AAGGACTCACT, ACTAATGTCATG, TATGAAGACATTT, or AATTGCATCACC) was deleted from the NOX4 promoter.

The above promoter reporters were transfected into Eca109, TE-1, or HEK293T cells or were cotransfected with the ATF3-pcDNA3.1 expression vector. The indicated cells were harvested, and the promoter activity of NOX4 was measured with a dual luciferase reporter assay kit (Promega, WI, USA) according to the manufacturer’s instructions. In brief, the indicated cells were seeded in 24-well plates, transfected with reporter plasmids using Lipofectamine 3000 (Invitrogen, CA, USA) and incubated for 24 h. After transfection, cells were collected with passive buffer, and firefly and Renilla luciferase activities were measured with a Dual Luciferase Reporter Assay System (Promega, WI, USA) and an illuminometer.

### Bioinformatic analyses

RNA-seq expression data (quantified by count and fragments per kilobase per million mapped reads (FPKM)) in the TCGA-ESCA dataset were downloaded from the Genomic Data Commons (GDC) portal (https://portal.gdc.cancer.gov/). TCGA dataset manipulation and analysis as well as GSEA were implemented in R 3.6.0.

For microarray data analysis, normalized data (GEO accession no. GSE20347, no. GSE23400, no. GSE45670 and no. GSE75241) and the corresponding probe annotation data were downloaded from the NCBI GEO database (https://www.ncbi.nlm.nih.gov/gds). Microarray data manipulation and analysis were performed in R 3.6.0.

IFI6 coexpression analysis was based on calculation of the Pearson correlation coefficient (r_PCC_) values of the correlations between the expression levels of IFI6 and potential mRNAs in the four above GEO datasets. mRNAs for which |r_PCC_| > 0.5 and *P* < 0.05 were recommended for further analysis (Table S6).

Transcription factor-promoter binding predictions were performed with JASPAR (http://jaspar.genereg.net).

Survival analysis of ESCC patients in TCGA database were carried out with web-based analysis tool Kaplan-Meier Plotter (http://www.kmplot.com).

## Results

### IFI6 expression is elevated in ESCC and correlated with disease stages

**Fig. 1:**
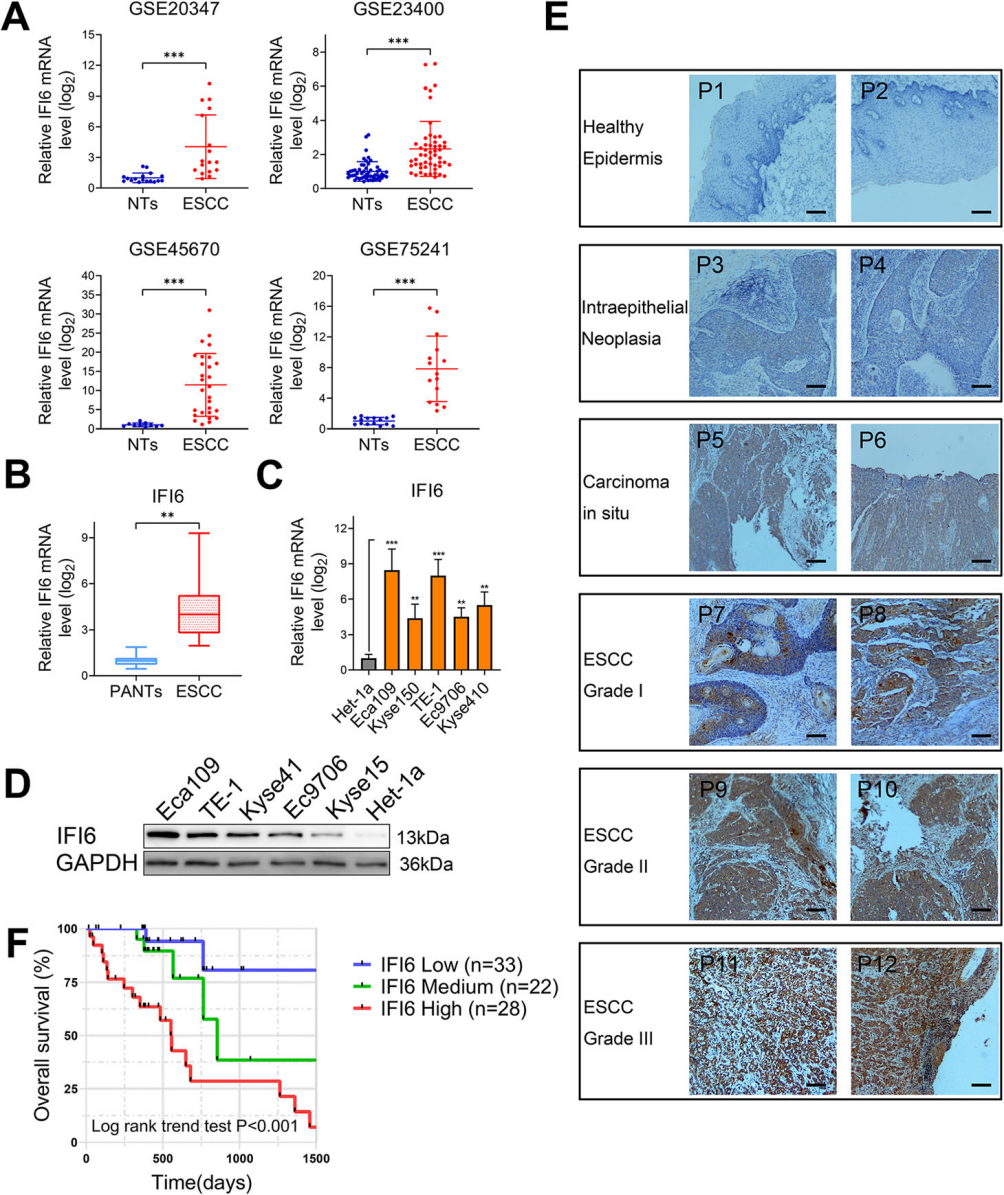
IFI6 expression is elevated in ESCC and predicts a poor prognosis. **a**. IFI6 expression levels in ESCC tissues vs. non-tumorous tissues (NTs) were analyzed using the GEO database (GEO accession no. GSE20347, no. GSE23400, no. GSE45670 and no. GSE75241). The data were normalized to IFI6 expression in NTs and are presented as the means and SDs. Statistical significance was determined by a modified t-test (implemented via the *limma* package in R). ***P < 0.005. **b**. Relative IFI6 mRNA levels in 23 ESCC tissues and paired adjacent non-tumorous tissues (PANTs) were measured via RT-PCR. The data were normalized to IFI6 expression in PANTs and are presented as the means and SDs (*n* = 3). Statistical significance was determined by a two-tailed Student’s t-test. **P < 0.01. **c**. IFI6 mRNA levels in the esophageal squamous epithelial cell line Het-1a as well as a panel of ESCC cell lines. The data were normalized to IFI6 expression in Het-1a cells and are presented as the means and SDs (*n* = 3). Statistical significance was determined by two-tailed Student’s t-test. **P < 0.01, ***P < 0.005. **d**. Representative Western blot showing the protein abundance of IFI6 in the esophageal squamous epithelial cell line Het-1a as well as a panel of ESCC cell lines. GAPDH was used as the loading control. **e**. Representative immunohistochemical images in the healthy human esophageal epidermis, esophageal hyperplasia and ESCC tissues at increasing disease stages; P1-P12 indicate the donor number. Magnification: 40×, Scale bar: 50 μm. **f**. Plots visualizing the Kaplan-Meier plot and log-rank-trend-test for ESCC patients from 83 cases of IHC cohort separated into groups of high, medium and low IFI6 expression levels. Statistical significance was determined via the log-rank test and log-rank trend test

Initially, we analyzed raw microarray data of ESCC patients from several subsets of Gene Expression Omnibus (GEO) datasets by a bioinformatic approach and found that IFI6 expression was dramatically upregulated in ESCC tissues compared with non-tumorous esophageal tissues (Fig. 1a). We also validated these data by assessing IFI6 expression in 23 ESCC samples and corresponding normal esophageal tissues from patients in our hospital (Fig. 1b). Given that ESCC is heterogenous both genetically and in its clinical manifestation, we further explored the IFI6 expression profile across a series of genetically distinct ESCC cell lines. As expected, IFI6 expression patterns were heterogeneous; however, IFI6 expression was higher in all five ESCC cell lines tested than in the normal esophageal squamous epithelial cell lines (Fig. 1c). The high levels of IFI6 expression were further confirmed at the protein level via Western blot analysis (Fig. 1d).

To explore the clinical relevance of IFI6, we assessed the IFI6 abundance in non-cancerous esophageal tissues and in samples with progressively aggressive characteristics: esophageal hyperplasia, esophageal carcinoma in situ, low-grade ESCC, moderate-grade ESCC, and high-grade ESCC using IHC. The image in Fig. 1e shows that IFI6 staining is absent in the healthy esophageal epidermis but starts to increase in esophageal hyperplasia and remains considerably high at more advanced ESCC stages. Consistent with this observation, statistical analysis revealed that aberrant IFI6 protein levels were significantly correlated with tumor grade, the depth of invasion, TNM stage (Table S1). Moreover, we categorized our 83 cases of ESCC cohort into IFI6-High, IFI6-Medium and IFI6-Low groups and then analyzed the survival probability of each group via Kaplan-Meier and log-rank analyses. As shown in Fig. 1f, high IFI6 expression predicted an unfavorable prognosis (IFI6-High vs. IFI6-Medium, P_log rank_ = 0.036; IFI6-Medium v.s IFI6-Low, P_log rank_ = 0.012; P_log rank trend_ < 0.001). The univariate and multivariate Cox regression analyses demonstrated that IFI6 expression was an independent prognostic factor in ESCC (Table S2). Ultimately, we analyzed the survival probability of patients categorized as IFI6-high versus patients categorized as IFI6-low in the TCGA-ESCA dataset through the web-based tool Kaplan-Meier Plotter (*www.kmplot.com**)*. Compared with patients categorized as IFI6-low, patients categorized as IFI6-high exhibited a substantially reduced survival probability (Figure S1A). These data support a potential functional role of IFI6 in ESCC carcinogenesis and development.

### Bioinformatic analyses indicate the potential role of IFI6 in ESCC is associated cell proliferation, apoptosis and ROS production

**Fig. 2:**
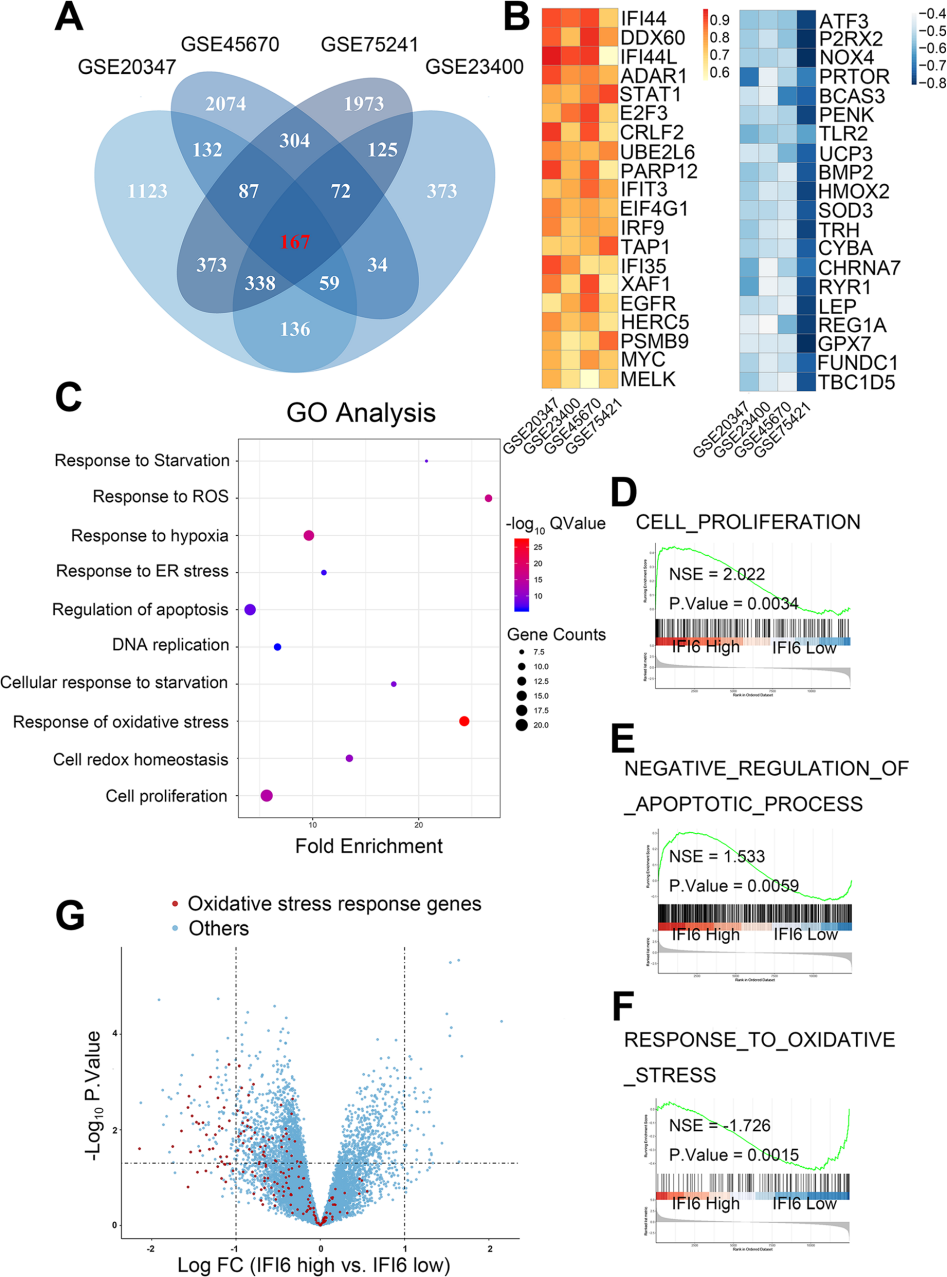
Bioinformatic analysis and functional characteristics of IFI6 in ESCC datasets from GEO and TCGA. **a**. Venn diagram showing the intersection of mRNAs coexpressed with IFI6 in four GEO ESCC datasets (GEO accession no. GSE20347, no. GSE23400, no. GSE45670 and no. GSE75241) as determined by gene coexpression analysis. **b**. Correlation heatmap presenting the 20 mRNAs most significantly positively correlated with IFI6 (left) as well as the 20 mRNAs most significantly negatively correlated with IFI6 (right) in the four indicated GEO datasets, ranked by the *P*-value. **c**. Bubble plot of the top 10 GO BP terms identified by GO enrichment analysis of the set of genes coexpressed with IFI6, ranked by the corrected P-value (Q-value). The bubble colors indicate the Q-values, and the bubble sizes represent the number of genes in the corresponding GO term. BP, Biological Process. GO, Gene Ontology. **d-f**. ESCC patients in the TCGA database were divided into a high-IFI6 group and a low-IFI6 group according to their IFI6 expression level. Transcriptome analysis and GSEA were performed to compare the two groups in the enrichment of gene sets belonging to cell proliferation (**d**), negative regulation of apoptotic process (**e**), response to oxidative stress (**f**), respectively. GSEA enrichment diagrams are shown. NES: normalized enrichment score. **g**. Volcano diagram of ESCC patient data derived from the TCGA database separated by high vs. low abundance of IFI6. The red dots represent mRNAs involved in the response to oxidative stress. The x-axis denotes the fold change (log_2_ scale), whereas the y-axis indicates statistical significance (−log_10_ format)

Given that the in-depth characterization of its function in ESCC has not been investigated, we next sought to explore the potential function of IFI6 in more detail. We initially analyzed genes coexpressed with IFI6 in the abovementioned four GEO ESCC microarray datasets. According to the screening criteria (|r_PCC_| ≥ 0.5, *P*-value< 0.05), 167 genes were coexpressed with IFI6 in all four of the above datasets (Fig. 2a, b). To broadly consider the cellular pathways in which IFI6 might play a role, we performed Gene Ontology (GO) enrichment analysis via the Database for Annotation, Visualization, and Integrated Discovery (DAVID) tool (*https://david.ncifcrf.gov*) for the 167 coexpressed genes in terms of biological process (BP). Genes whose expression correlated with IFI6 were enriched in the following BP terms: response to starvation, response to reactive oxygen species, cell proliferation, regulation of apoptotic process, response to oxidative stress, and response to ER stress (Fig. 2c). To further consolidate the above GO analysis results, we grouped ESCC patients from The Cancer Genome Atlas (TCGA) database (TCGA-ESCA dataset) based on their IFI6 expression level. Gene set enrichment analysis (GSEA) demonstrated that high IFI6 levels led to upregulation of gene sets related to cell proliferation and negative regulation of apoptosis (Fig. 2d, e). In addition, patients with higher IFI6 levels exhibited lower levels of genes related to the oxidative stress response (Fig. 2f, g).

Collectively, the results of these bioinformatic analyses led us to hypothesize that IFI6 can promote proliferation, inhibit apoptosis and ameliorate oxidative stress.

### IFI6 silencing inhibits cell growth, induces apoptosis through cellular ROS accumulation

**Fig. 3:**
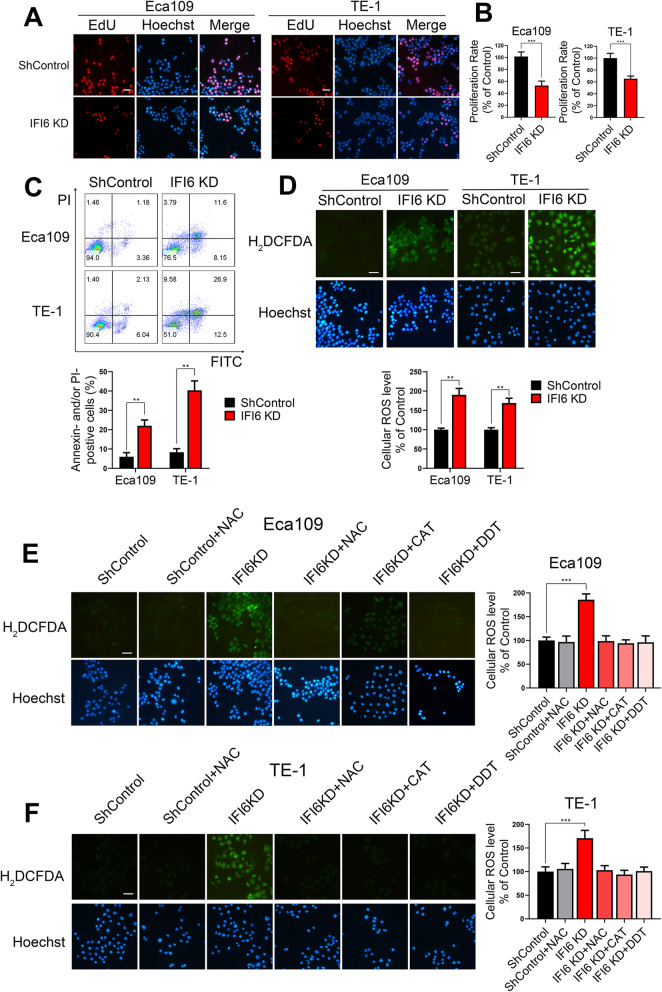
IFI6 promotes cell proliferation, inhibits apoptosis and ameliorates oxidative stress in ESCC. **a**-**b**. Representative images (**a**) and statistical quantification (**b**) of EdU staining in ESCC cell lines transfected with IFI6-shRNA or ShControl lentivirus. EdU: red, Hoechst 33342: blue. The data are presented as the means and SDs (*n* = 3). Scale bar: 20 μm. Statistical significance was determined by two-tailed Student’s t-test. ***P < 0.005. **c**. Representative images (upper) and statistical quantification (lower) of apoptotic and necrotic cell populations in ESCC cell lines, as determined by Annexin-V FITC/PI staining and flow cytometry. Cells with a FITC^−^ and PI^−^ signature were considered viable. Cells with a FITC^+^ and PI^−^ or a FITC^+^ and PI^+^ signature were considered nonviable. The data are presented as the means and SDs (*n* = 3). Statistical significance was determined by two-tailed Student’s t-test. **P < 0.01. **d**. Representative images (upper) and statistical quantification (lower) of ROS production assay results in ESCC cells. The indicated cells were stained with carboxy-H_2_DCFDA and observed under a fluorescence microscope. H_2_DCFDA: green, Hoechst 33342: blue. Scale bar: 20 μm. The data are presented as the means and SDs (n = 3). Statistical significance was determined by two-tailed Student’s t-test. **P < 0.01. **e-f**. Representative images (left) and statistical quantification (right) of ROS production assay results in Eca109 (**e**) and TE-1 (**f**). The indicated cells were preincubated with different ROS inhibitors, stained with carboxy-H_2_DCFDA and observed under a fluorescence microscope. H_2_DCFDA: green, Hoechst: blue. Scale bar: 20 μm. The data are presented as the means and SDs (*n* = 3). Statistical significance was determined by one-way ANOVA. **P < 0.01

To confirm the above bioinformatic analysis results, we chose Eca109 and TE-1 as our cellular models. IFI6-shRNA was transfected into ESCC cell lines. We also constructed stable ESCC cell lines ectopically expressing IFI6 as well as OEControl cell lines. Silencing as well as overexpression efficiencies were validated via qRT-PCR and Western blot assays (Figure S1B-E). Initially, an EdU assay was conducted, which indicated that cell growth, assessed as the percentage of EdU-positive cells, was significantly inhibited in IFI6-silenced ESCC cells compared with ShControl ESCC cells (Fig. 3a, b). To further demonstrate whether apoptosis could also play a role in the reduction in cell viability implied by the aforementioned bioinformatic analysis results (Fig. 2c, e), we next conducted annexin V-FITC/propidium iodide (PI) staining. Consistent with the EdU assay results, IFI6 inhibition significantly induced ESCC cell apoptosis (Fig. 3c). In the reciprocal experiment, cell proliferation markedly increased by IFI6 overexpression, whereas ESCC cell apoptosis was significantly inhibited following IFI6 overexpression (Figure S2A-C).

**Fig. 4:**
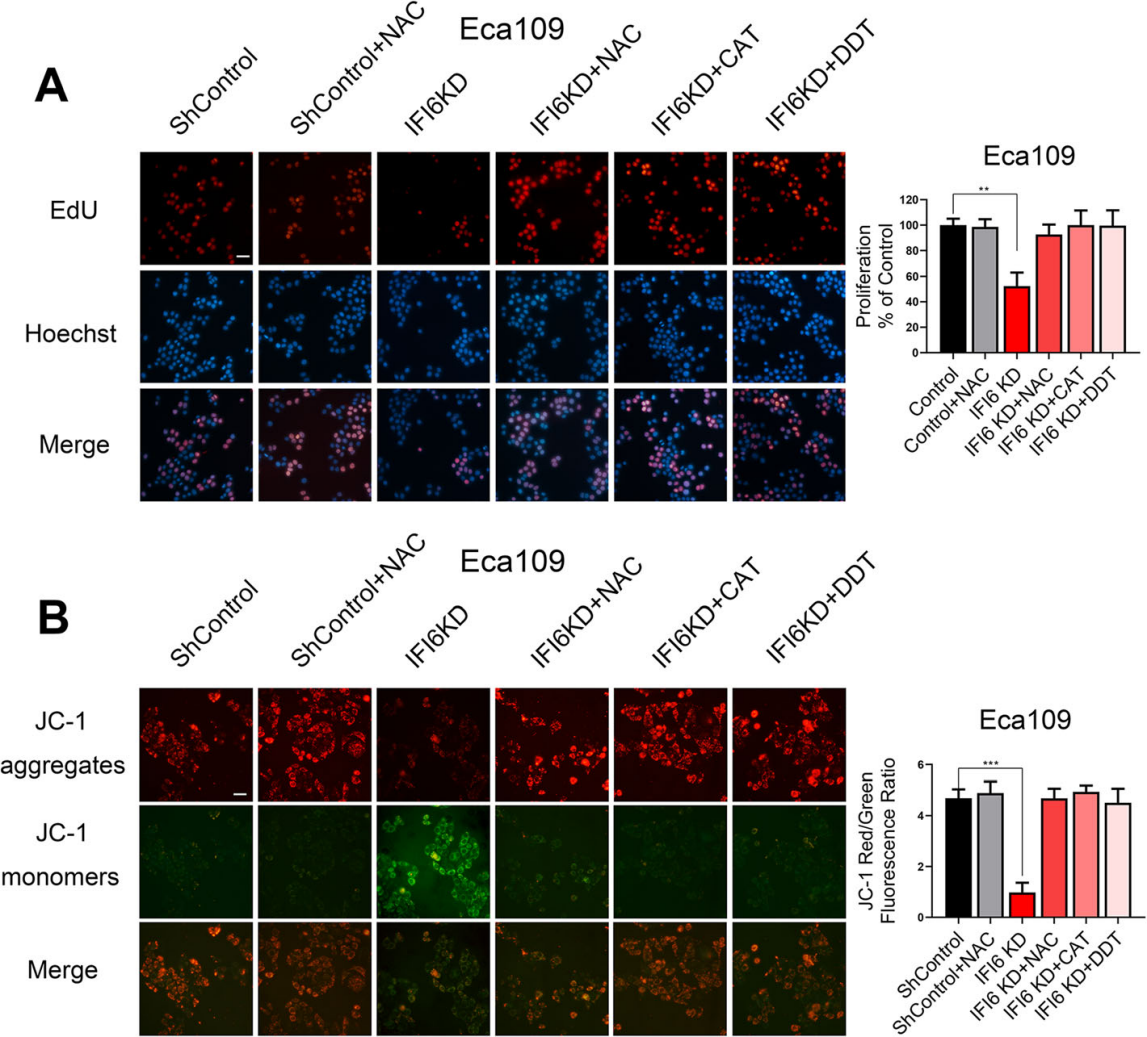
ROS accumulation is responsible for the IFI6 silencing-induced reduction in cell viability. **a**. Representative images (left) and statistical quantification (right) of EdU staining in the indicated Eca109 cells preincubated with different ROS inhibitors. EdU: red, Hoechst 33342: blue. Scale bar: 20 μm. The data are presented as the means and SDs (*n* = 3). Statistical significance was determined by one-way ANOVA. ***P < 0.005. **b**. Representative images (left) and statistical quantification (right) of the apoptosis assay results in Eca109 cells, as indicated by the mitochondrial membrane potential. The indicated cells were stained with JC-1 after preincubation with different ROS inhibitors. Cells stained with JC-1 are visible as either green (J-monomers) or red (J-aggregates) fluorescence. The apoptosis rate was calculated as the ratio of JC-1 aggregates to JC-1 monomers. Scale bar: 20 μm. The data are presented as the means and SDs (*n* = 3). Statistical significance was determined by one-way ANOVA. ***P < 0.005

We next examined the mechanism underlying the reduction in cell viability. Because IFI6 can ameliorate oxidative stress, as revealed by the above bioinformatic analysis results (Fig. 2c, f, g), we then investigated cellular ROS levels following IFI6 silencing. IFI6 inhibition significantly enhanced the generation of cellular ROS, as represented by the live cell ROS indicator carboxy-H_2_DCFDA (Fig. 3d), and IFI6 overexpression exhibited the opposite effect (Figure S2D). Accordingly, we sought to determine whether the augmented cellular oxidative environment caused by altered IFI6 expression is responsible for the reduction in cell viability. To test this hypothesis, we monitored the growth of IFI6-silenced Eca109 cells in the absence or presence of a reducing agent (dithiothreitol, DDT) (2 mM), the hydrogen peroxide scavenger (PEG-catalase, CAT) (100 U/ml) and an antioxidant (N-acetyl-L-cysteine, NAC) (200 μM). Treatment with all of these ROS-eliminating reagents indeed decreased the level of carboxy-H_2_DCFDA staining (Fig. 3e, f). Moreover, as shown in Fig. 4a, all these ROS-eliminating reagents effectively reversed the effect of IFI6 silencing on cell viability. Our findings were corroborated in TE-1 cells (Figure S3A). To further explore the effect of ROS on IFI6-mediated cell survival, we used the mitochondrial membrane potential indicator JC-1, an alternative method to detect mitochondria-mediated apoptosis as well as mitochondrial dysfunction. Consistent with the EdU assay results, treatment of ESCC cells with ROS inhibitors completely reversed IFI6 silencing-induced apoptosis (Fig. 4b, Figure S3B), which supported the role of ROS in the IFI6 silencing-induced reduction in cell viability.

### IFI6 modulates mitochondrial ROS production partially by regulating calcium influx through regulating the activity of mitochondrial Ca^2+^ uniporter

**Fig. 5:**
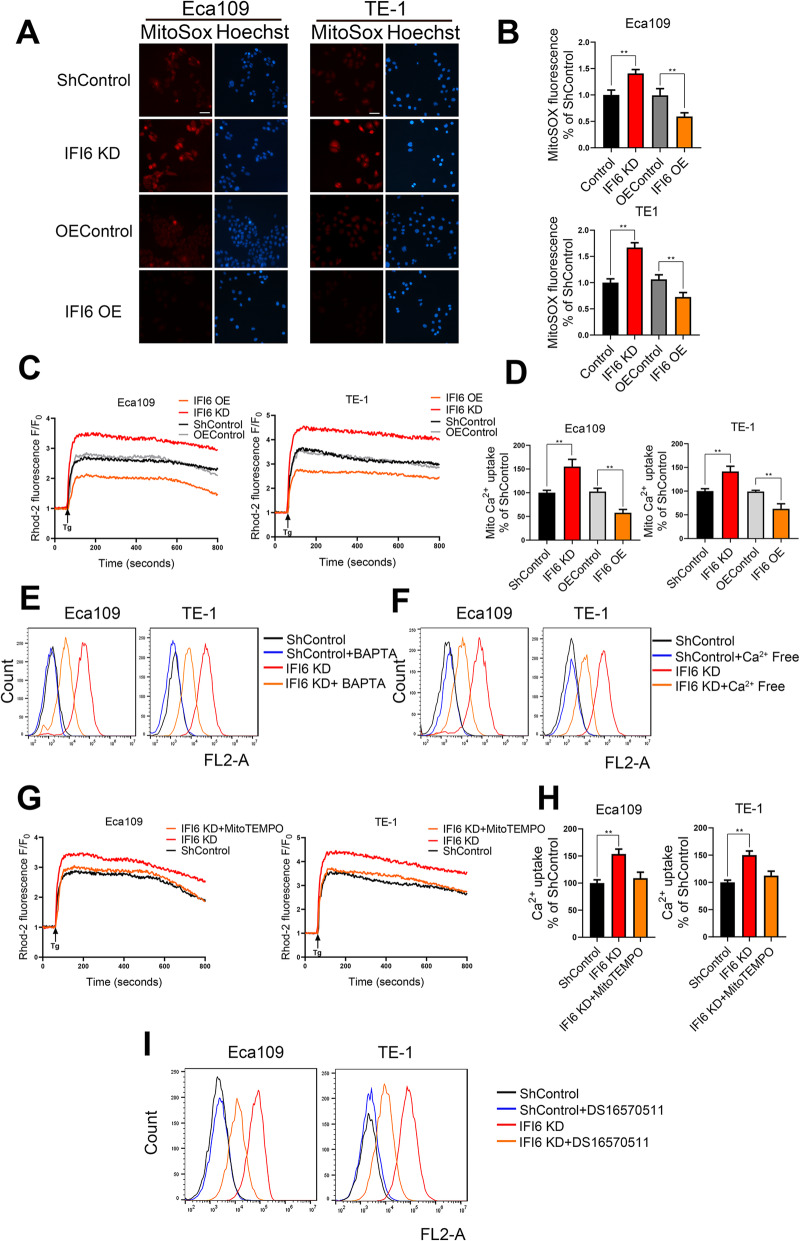
IFI6 modulates mitochondrial ROS production by regulating mitochondrial Ca^2+^ overload. **a**-**b**. Representative images (**a**) and statistical quantification (**b**) of mitochondrial ROS (mtROS) production assay results in ESCC cells. The indicated cells were stained with MitoSOX, and fluorescence was quantified under a fluorescence microscope. MitoSOX: red, Hoechst: blue. Scale bar: 20 μm. The data are presented as the means and SDs (*n* = 3). Statistical significance was determined by two-tailed Student’s t-test. **P < 0.01. **c-d**. Quantification (**c**) and statistical analysis (**d**) of the relative Rhod-2 AM fluorescence intensity time course in the indicated ESCC cells. The fluorescence intensity at each time point was recorded with an integrated spectrofluorometer at excitation and emission wavelengths of 550 nm and 580 nm, respectively. The relative fluorescence intensity was calculated as a percentage of the baseline fluorescence intensity, which was recorded during the first 60 s (F_0_). The arrow indicates the time at which Tg was added. The data are presented as the means and SDs (*n* = 3). Statistical significance was determined by two-tailed Student’s t-test. **P < 0.01. **e**. The indicated ESCC cells were incubated in the presence or absence of BAPTA-AM (2 μM) for 1 h, and mitochondrial ROS levels were then measured by MitoSOX staining followed by flow cytometry. **f**. The indicated ESCC cells were cultured in complete medium or calcium-deficient medium, and mitochondrial ROS levels were determined by MitoSOX staining followed by flow cytometry. **g-h**. Quantification (**g**) and statistical analysis (**h**) of the relative Rhod-2 AM fluorescence intensity time course in the indicated ESCC cells. The fluorescence intensity at each time point was recorded with an integrated spectrofluorometer at excitation and emission wavelengths of 550 nm and 580 nm, respectively. The relative fluorescence intensity was calculated as a percentage of the baseline fluorescence intensity, which was recorded during the first 60 s (F_0_). The arrow indicates the time at which Tg was added. The data are presented as the means and SDs (*n* = 3). Statistical significance was determined by one-way ANOVA. **P < 0.01. **i**. The indicated ESCC cells were incubated in the presence or absence of the MCU inhibitor DS16570511 (10 μM), and mitochondrial ROS levels were then measured by MitoSOX staining, followed by flow cytometry

We next sought to understand how IFI6 controls ROS production and therefore tumor cell viability. We reasoned that this regulatory mechanism is likely to be related to mitochondria, given that mitochondrial respiration is the major source of cellular ROS. To explore the role of mitochondria in ROS generation after alterations in IFI6 expression, we used the mitochondrial ROS-specific probe MitoSOX to detect mitochondrial ROS production in Eca109 and TE-1 cells. IFI6 silencing led to an elevation in mitochondrial ROS levels, whereas mitochondrial ROS production was significantly inhibited in cells overexpressing IFI6 (Fig. 5a, b). The above observations indicated that the expression level of IFI6 directly affects mitochondrial ROS production. As reported, mitochondrial Ca^2+^ signaling profoundly influences mitochondrial OXPHOS and, therefore, ROS generation [26]. We next sought to determine whether IFI6 impacts calcium dynamics and thus mitochondrial ROS production. To this end, we observed the effect of thapsigargin (Tg), a plant-derived sesquiterpene lactone that increases the cytosolic calcium concentration by inhibiting ER-resident ATP-dependent calcium pumps and activating store-operated Ca^2+^ entry (SOCE) channels [27], on mitochondrial calcium dynamics. By using the mitochondrial calcium indicator Rhod-2 AM, we showed that IFI6-silenced ESCC cells exhibited substantially increased calcium uptake following the Tg-mediated increase in the cytosolic calcium concentration (Fig. 5c, d). Previous studies showed that calcium influx into mitochondria leads to mitochondrial dysfunction and contributes to ROS generation as well as pathological induction of cell death [28, 29]. We next sought to determine whether inhibiting calcium uptake by mitochondria would reverse IFI6 silencing-induced mitochondrial ROS production. However, suppression of mitochondrial Ca^2+^ uptake by Ca^2+^ chelators with 1,2-bis(o-aminophenoxy) ethane-N,N,N′,N′-tetraacetic acid (BAPTA) only partially reversed the IFI6 silencing-induced mitochondrial ROS generation (Fig. 5e). By removing Ca^2+^ from the cell culture medium, we obtained similar results (Fig. 5f). Based on the above findings, we sought to determine whether the abundance of IFI6 might modulate the expression of mitochondrial calcium channels. For this purpose, we evaluated the expression levels of voltage-dependent anion channel type 1 (VDAC1), mitochondrial Ca^2+^ uniporter (MCU) and mitochondrial sodium calcium lithium exchanger (NCLX) by Western blotting and RT-PCR. However, as indicated in Figure S4A-B, no overt alteration of VDAC1, MCU, and NCLX expression occurred in TE-1 and Eca109 cells after the downregulation of IFI6. To investigate this phenomenon in more detail, we also sought to determine whether the redox status of the mitochondrial MCU complex plays a role in mitochondrial calcium uptake [30]. To this end, we used the mitochondria-targeted ROS scavenger MitoTEMPO (20 μM) to evaluate the effect of redox status on mitochondrial calcium uptake in parental and IFI6-silenced cells. As shown in Fig. 5g-h, treatment with this mitochondrial ROS scavenger completely reversed the IFI6 silencing-induced increase in mitochondrial Ca^2+^ uptake. Furthermore, following IFI6 ablation, pharmacological inhibition of MCU with DS16570511 partially prevented IFI6 silencing-induced mitochondrial ROS generation (Fig. 5i).

Taken together, these observations imply that after IFI6 knockdown, the increased level of mitochondrial ROS may disrupt the redox regulation of the mitochondrial MCU, which in turn leads to mitochondrial calcium overload and, subsequently, mitochondrial ROS accumulation. Given the fact that blocking mitochondrial Ca^2+^ only partially attenuated IF6 silencing-induced mitochondrial ROS production, mitochondrial Ca^2+^ influx disruption plays a role in, but may not be the only cause of, elevated mitochondrial ROS production. The driving contributor to mitochondrial ROS generation following IFI6 ablation remains to be explored.

### IFI6 regulates mitochondrial ATP production and mitochondrial oxidative phosphorylation

**Fig. 6:**
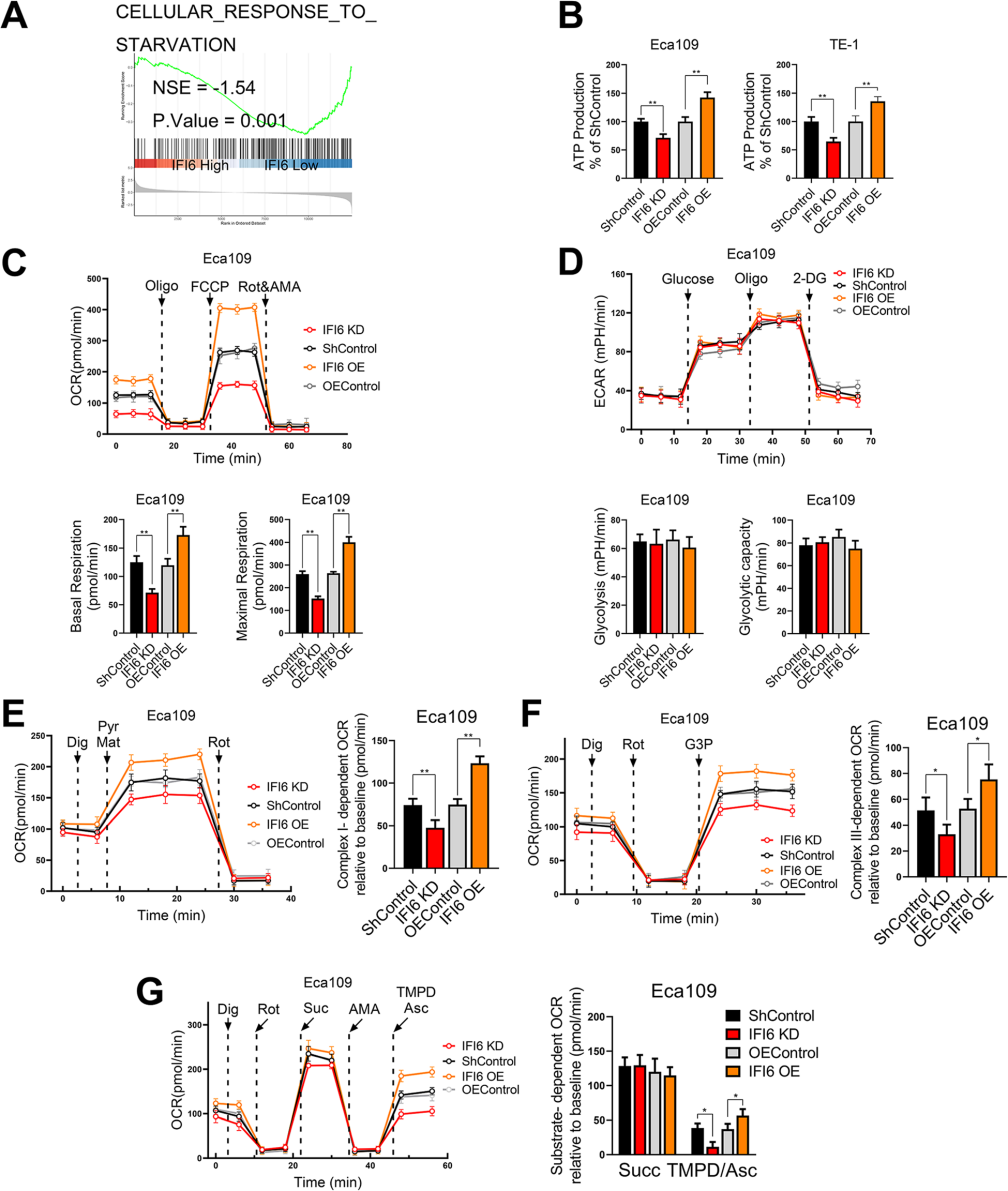
IFI6 modulates mitochondrial ATP production and the oxidative phosphorylation efficiency. **a**. ESCC patients in the TCGA database were divided into a high-IFI6 group and a low-IFI6 group according to their IFI6 expression level. GSEA was performed to compare the two groups. NES: normalized enrichment score. **b**. Quantitative results of ATP production in ESCC cells. Total ATP levels were measured in the indicated cells via a luminescence assay. Data were normalized to the ATP level in ShControl cells and are presented as the means and SDs (*n* = 3). Statistical significance was determined by a two-tailed Student’s t-test. **P < 0.01. **c**. Representative plots (upper) and quantitative results (bottom) of the cellular OCR, basal and maximal respiration rates in the different groups. The indicated ESCC cells were subjected to extracellular flux analysis in the Seahorse XF instrument. The arrows and dotted lines indicate the addition of Oligo (oligomycin) (1 μM), FCCP (Carbonyl cyanide 4-(trifluoromethoxy) phenylhydrazone) (0.5 μM) and Rot&AMA (Rotenone and Antimycin A) (0.5 μM each). The data are presented as the means and SDs (*n* = 3). Statistical significance was determined by two-tailed Student’s t-test. **P < 0.01. **d**. Representative plots (upper) and quantitative results (bottom) of the real-time ECAR, glycolysis and glycolytic capacity assays in the indicated ESCC cells. The ECAR was determined following sequential addition of glucose (10 mM), oligomycin (1 μM) and 2-DG (100 mM). Glycolysis was measured by subtracting the ECAR after glucose addition from the ECAR before glucose addition. The glycolytic capacity was calculated by subtracting the ECAR after oligomycin treatment from the ECAR before glucose addition. The data are presented as the means and SDs (n = 3). Statistical significance was determined by a two-tailed Student’s t-test. **e**. Representative plots (left) and quantitative results (right) of the complex I-dependent OCR in the different groups. Pyruvate (Pyr) (5 mM) and malate (Mat) (5 mM) were added to digitonin (Dig)-permeabilized cells, and the OCR was monitored. The data are presented as the means and SDs (*n* = 3). Statistical significance was determined by two-tailed Student’s t-test. **P < 0.01. **f**. Representative plots (left) and quantitative results (right) of the complex III-dependent OCR in the different groups. Rotenone was added to digitonin-permeabilized cells to inhibit complex I, after which G3P (5 mM) was added, and the OCR was monitored. The data are presented as the means and SDs (*n* = 3). Statistical significance was determined by two-tailed Student’s t-test. **P < 0.01. **g**. Representative plots (left) and quantitative results (right) of the complex II-, and complex IV-dependent OCRs in the different groups. Rotenone (1 μM) was added to inhibit complex I; succinate (Suc) (5 mM), Antimycin (AMA) (1 μM) and TMPD/ascorbate (500 μM and 5 mM, respectively) were then added to digitonin-permeabilized cells, and the OCR was monitored. The data are presented as the means and SDs (*n* = 3). Statistical significance was determined by two-tailed Student’s t-test. **P < 0.01

Several lines of evidence have demonstrated that the ETC during OXPHOS constitutes the principal source of mitochondrial ROS production and that mitochondrial supercomplex formation can modulate this process [31]. Consistent with these observations, the results of our GSEA of TCGA data implied a state of energy deprivation (Fig. 6a). To clarify the alternative reason why mitochondrial ROS production was increased following IFI6 knockdown, we measured ATP levels in ESCC cell lines after IFI6 silencing or overexpression and compared them with those in the corresponding parental cell lines. Eca109 and TE-1 cells ectopically expressing IFI6 exhibited higher ATP levels than the corresponding control cells, while cells with IFI6 silencing had lower ATP levels than the corresponding control cells (Fig. 6b).

To further assess the effect of IFI6 on cellular bioenergetics, we conducted extracellular flux analysis to assess the extracellular acidification rate (ECAR) as well as the oxygen consumption rate (OCR), which are assumed to be representative indicators of lactic acid generation and mitochondrial OXPHOS, respectively. Accordingly, we measured the OCR of ESCC cells in a Seahorse Bioanalyzer with the sequential addition of several mitochondrial stressors, such as Oligo, FCCP, Rot and AMA. As shown in Fig. 6c, the basal cellular OCR and maximum respiration rate were significantly elevated in IFI6-overexpressing Eca109 cells compared with parental Eca109 cells. On the other hand, IFI6 silencing via shRNA decreased both the resting OCR and the maximum OCR in Eca109 cells (Fig. 6c). Similar results were also observed in TE-1 cells (Figure S5A). However, there was no overt difference in ECAR between ESCC cells ectopically expressing IFI6 and parental ESCC cells (Fig. 6d, Figure S5B).

To further understand the mechanism underlying these alterations in mitochondrial energy and ROS generation, we measured the OCR in the presence of substrates for complex I (pyruvate, malate), complex II (succinate), complex III (glyceraldehyde 3-phosphate, G3P) or complex IV (N,N,N′,N′-tetramethyl-1,4-phenylenediamine (TMPD)/ascorbate). The complex I-specific OCR in IFI6-silenced cells was substantially reduced compared with that in parental cells; in contrast, IFI6 overexpression significantly increased the complex I-specific OCR in both Eca109 and TE-1 cells (Fig. 6e, Figure S5C). In addition, the OCRs specific to complexes III and IV exhibited similar patterns (Fig. 6f-g, Figure S5D-E). However, in Eca109 and TE-1 cells, the OCR supported by complex II remained unchanged (Fig. 6g, Figure S5E), which implied that IFI6 might alter respiratory complex function at multiple sites (namely, complex I, complex III and complex IV).

These observations confirmed that altered IFI6 expression levels might play a role in energy handling and mitochondrial dysfunction.

### IFI6 regulates the mitochondrial redox status by modulating respiration supercomplex assembly

**Fig. 7:**
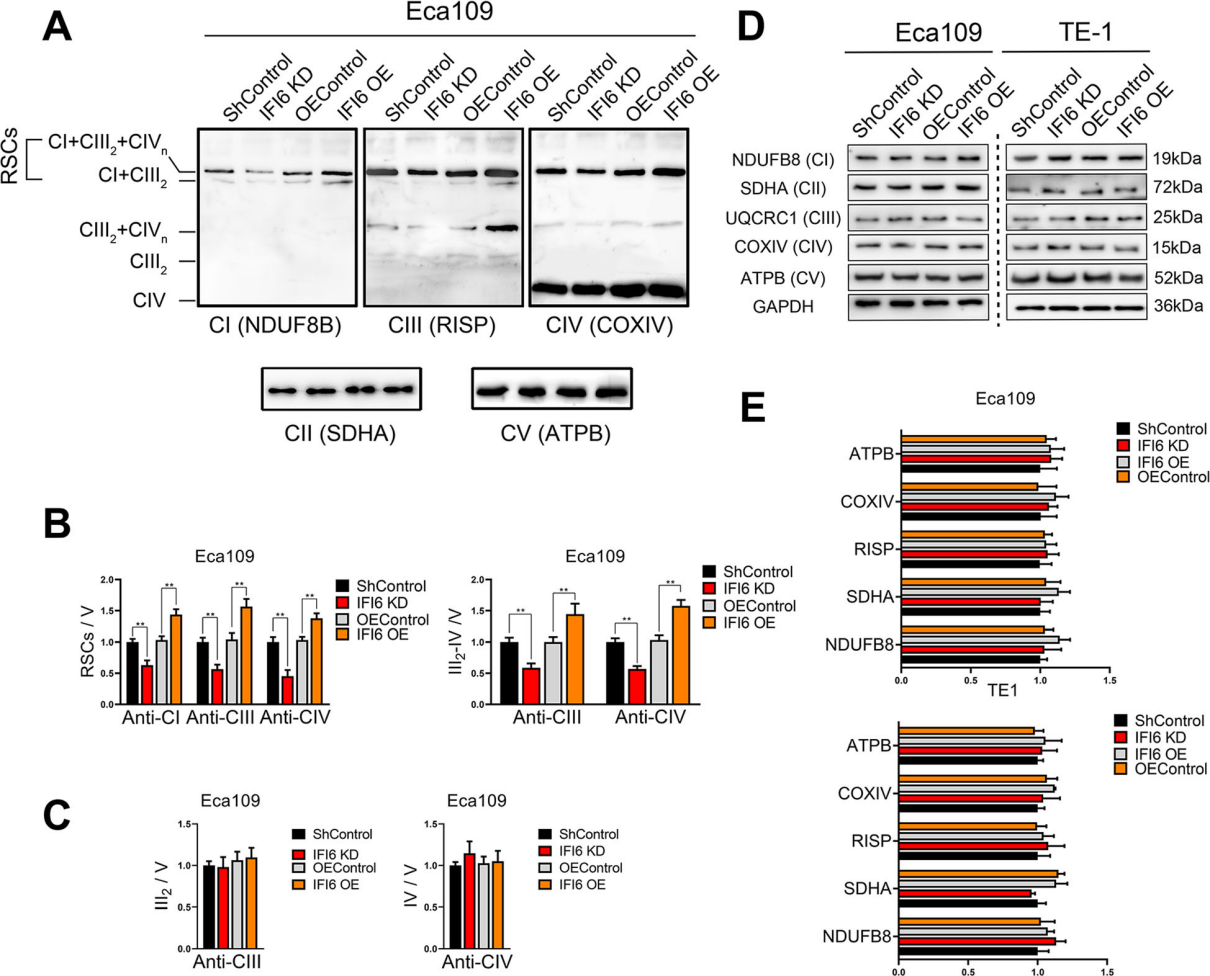
IFI6 modulates mitochondrial ROS production and OXPHOS efficiency by regulating mitochondrial supercomplex assembly. **a**. Mitochondrial proteins extracted from the indicated ESCC cells were solubilized with digitonin and subjected to BN-PAGE followed by immunoblotting. Mitochondrial supercomplexes were first visualized by incubation with antibodies against complex I (CI, NDUFB8), and the membrane was then stripped and reprobed with antibodies against complex III (CIII, RISP). The membrane was then sequentially probed with antibodies against complex IV (CIV, COXIV), complex II (CII, SDHA) and complex V (CV, ATPB). **b-c**. Quantitative results for experiments shown in **a**. Data are presented as mean and SD (*n* = 3). Statistical significance was determined by two-tailed student’s t-test. **P < 0.01. **d-e**. Representative image (**d**) and quantitative results (**e**) of immunoblotting with antibodies against the indicated individual ETC complexes (I through V). GAPDH was used as the loading control. The data are presented as the means and SDs (*n* = 3). Statistical significance was determined by a two-tailed Student’s t-test

Previous studies reported that mitochondrial ROS production is closely interlinked with OXPHOS, whereas impaired respiratory complex function and respirasome formation enhance ROS production and impair ATP generation [32, 33]. We further assessed the contribution of IFI6 to OXPHOS complex assembly via blue native polyacrylamide gel electrophoresis (BN-PAGE) followed by immunoblotting. We used antibodies against NDUFB8 (complex I), SDHA (complex II), RISP (complex III), COXIV (complex IV) and ATPB (complex V) to detect respiratory complexes as well as respiratory supercomplexes (RSCs) in mitochondrial preparations from Eca109 and TE-1 cells. Consistent with previous studies, in all groups tested, complex II and complex V existed mainly as individual entities, whereas complexes I, III and IV were assembled into supercomplexes (Fig. 7a, Figure S6A). Moreover, silencing IFI6 decreased the signal of NDUFB8, RISP or COXIV at the CI + CIII_2_ + CIV_n_ and CI + CIII_2_ positions compared with those in parental ESCC cells. The signals of CI + CIII_2_ + CIV_n_ and CI + CIII_2_ RSCs were elevated in ESCC cells ectopically expressing IFI6 compared to parental cells (Fig. 7b, Figure S6B). In addition, IFI6 ablation resulted in a decreased signal at the CIII_2_ + CIV_n_ position, while overexpressing IFI6 had the opposite effect, as detected by immunoblotting with anti-RISP and anti-COXIV antibodies (Fig. 7b, Figure S6B). However, no consistent changes were observed in the levels of complex III dimers or complex IV monomers following alteration of IFI6 expression in either Eca109 or TE-1 cells (Fig. 7c, Figure S6C). These observations were consistent with those of the aforementioned extracellular flux analysis. We further assessed the abundance of individual respiratory complexes and showed that the expression level of IFI6 did not affect the expression of individual respiratory complexes, as evaluated by NDUFB8 (complex I), SDHA (complex II), RISP (complex III), COXIV (complex IV) and ATPB (complex V) expression (Fig. 7d, e), indicating that the changes in mitochondrial supercomplex formation are not due to alterations in the expression of individual OXPHOS complex subunits. Taken together, these observations indicate the relevance of IFI6 in the assembly of RSCs as well as CIII_2_ + CIV_n_ in ESCC.

Collectively, our data suggested two causes of elevated mitochondrial ROS following IFI6 silencing: first and the driving cause, impaired respiratory complex function and supercomplex assembly, which decreases the efficiency of OXPHOS and in its turn accumulates mitochondrial ROS; Secondly, the altered redox state of MCU induces mitochondrial Ca^2+^ overload and subsequently adds to mitochondrial ROS production.

### IFI6 depletion-mediated ATP shortage activates ER stress and subsequent cellular ROS generation through the ATF3-NOX4 axis

**Fig. 8:**
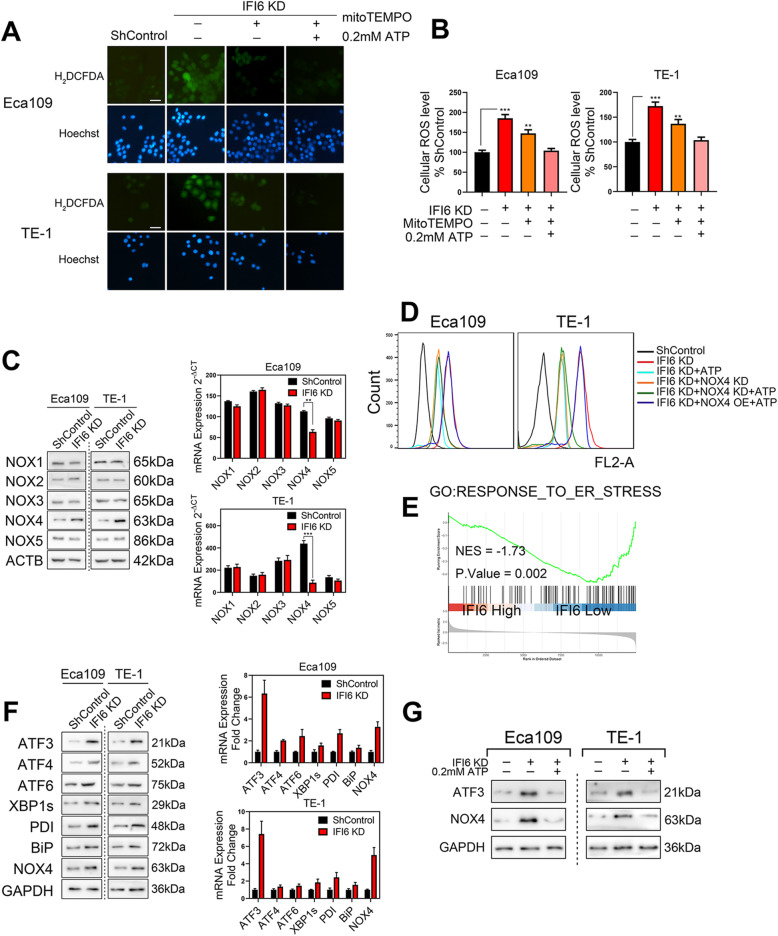
IFI6 silencing elevates ROS levels via the ATF3-NOX4 axis. **a**-**b**. Representative images (**a**) and statistical quantification (**b**) of ROS production assay results in ESCC cells. The indicated cells were treated with MitoTEMPO (20 μM) or exogenous ATP (0.2 mM), stained with carboxy-H_2_DCFDA and observed under a fluorescence microscope. H_2_DCFDA: green, Hoechst: blue. Scale bar: 20 μm. The data are presented as the means and SDs (n = 3). Statistical significance was determined by two-tailed Student’s t-test. **P < 0.01, ***P < 0.005. **c**. Immunoblot image (left) and RT-PCR results (right) for a panel of NOX isoforms in ESCC cells with stable IFI6 knockdown. GAPDH was used as the internal control. The data are presented as the means and SDs (n = 3). Statistical significance was determined by two-tailed Student’s t-test. **P < 0.01, ***P < 0.005. **d**. The indicated Eca109 and TE-1 cells were treated in the absence or presence of 0.2 mM exogenous ATP, and the cellular ROS level was measured by carboxy-H_2_DCFDA staining followed by flow cytometry. **e**. ESCC patients in the TCGA database were divided into a high-IFI6 group and a low-IFI6 group according to their IFI6 expression level. GSEA was performed to compare the two groups. NES: normalized enrichment score. **f**. Immunoblot image (left) and RT-PCR results (right) of a series of ER stress markers in ESCC cells with stable IFI6 knockdown. GAPDH was used as the internal control. **g**. The indicated Eca109 and TE-1 cells were treated in the absence or presence of 0.2 mM exogenous ATP. Then, protein lysates were collected and subjected to immunoblotting to assess the expression of IFI6, ATF3 and NOX4. GAPDH was used as the loading control

We next sought to rule out a role for alternative mechanisms in the increase in cellular ROS accumulation in addition to mitochondrial ROS production following IFI6 silencing in ESCC cells. For this purpose, we used carboxy-H_2_DCFDA to measure cellular ROS levels in control and IFI6-silenced cells. As expected, IFI6 depletion led to a dramatic increase in cellular ROS levels. Intriguingly, IFI6-KD ESCC cells treated with MitoTEMPO (20 μM), a mitochondria-specific antioxidant, exhibited only a partial reversal of IFI6 silencing-induced cellular ROS generation compared with vector-treated ESCC cells (Fig. 8a, b). However, the combination treatment with MitoTEMPO and exogenous ATP led to a significantly greater decrease in cellular ROS levels than each compound alone (Fig. 8a, b), implying that in addition to mitochondria, IFI6 silencing induces ROS production via another source and that supplying cells with exogenous ATP could block this pathway.

In most cells, mitochondria and the activities of NADPH oxidases (NOXs), which are located mainly in membranes such as the ER membrane, are the two primary sources of cellular ROS generation [34, 35]. We next analyzed the expression levels of the five different NOX isoforms in ESCC cells after IFI6 knockdown. Among a panel of NOX isoforms, NOX4 was substantially upregulated following IFI6 silencing in Eca109 and TE-1 cells (Fig. 8c). To validate these observations via functional analysis, we examined the role of NOX4 in the production of cellular ROS in IFI6-silenced ESCC cells using carboxy-H_2_DCFDA. As shown previously, cellular ROS levels increased after IFI6 ablation, while this increase was mitigated after treatment with NOX4 shRNA. Intriguingly, treatment of ESCC cells with either NOX4 shRNA alone or exogenous ATP substantially suppressed ROS production, while the combination of these two factors did not further decrease cellular ROS levels in IFI6-silenced ESCC cells. Furthermore, the exogenous ATP-induced decrease in cellular ROS was reversed by ectopic NOX4 expression in IFI6-silenced ESCC cells (Fig. 8d). These patterns suggest that mitochondrial dysfunction and an ATP shortage might contribute to cellular ROS production mediated by induction of NOX4 overexpression.

To explain the mechanism by which ATP deprivation leads to this substantial increase in NOX4 levels, we reasoned that this increase is likely to be correlated with induction of ER stress. Previous studies showed that ER stress can be induced by various means, including ATP deprivation [36]. Consistent with this observation, our aforementioned GSEA of TCGA data also revealed a state of energy deprivation as well ER stress induction (Figs. 8e, 6a). Therefore, we tested this hypothesis by measuring the expression levels of several ER stress markers, such as the transcription factors ATF3, ATF4, ATF6, and X-box binding protein 1 (XBP1s), as well as protein disulfide isomerase (PDI) binding immunoglobulin protein (BiP), following IFI6 silencing via PCR and Western blotting. We demonstrated consistent activation of ER stress in IFI6-silenced cells and concomitant elevation of NOX4 mRNA levels in Eca109 cells. This phenomenon was corroborated in TE-1 cells (Fig. 8f). Among these ER stress mediators, the transcription factor ATF3 exhibited the most dramatic elevation following IFI6 knockdown, implying that ATF3 might play a pivotal role in IFI6 silencing-mediated ER stress. To further characterize the molecular mechanism underlying overexpression of NOX4 elicited by ER stress following energy shortage, we treated IFI6-silenced ESCC cells with or without exogenous ATP and found that ATP fully rescued the increases in ATF3 and NOX4 expression induced by IFI6 silencing (Fig. 8f). These results indicate that IFI6 silencing is the initial event and is followed by ATP shortage and ER stress induction with subsequent elevation of NOX4 expression, which finally leads to increased cellular ROS levels independent of the mitochondrial source.

**Fig. 9:**
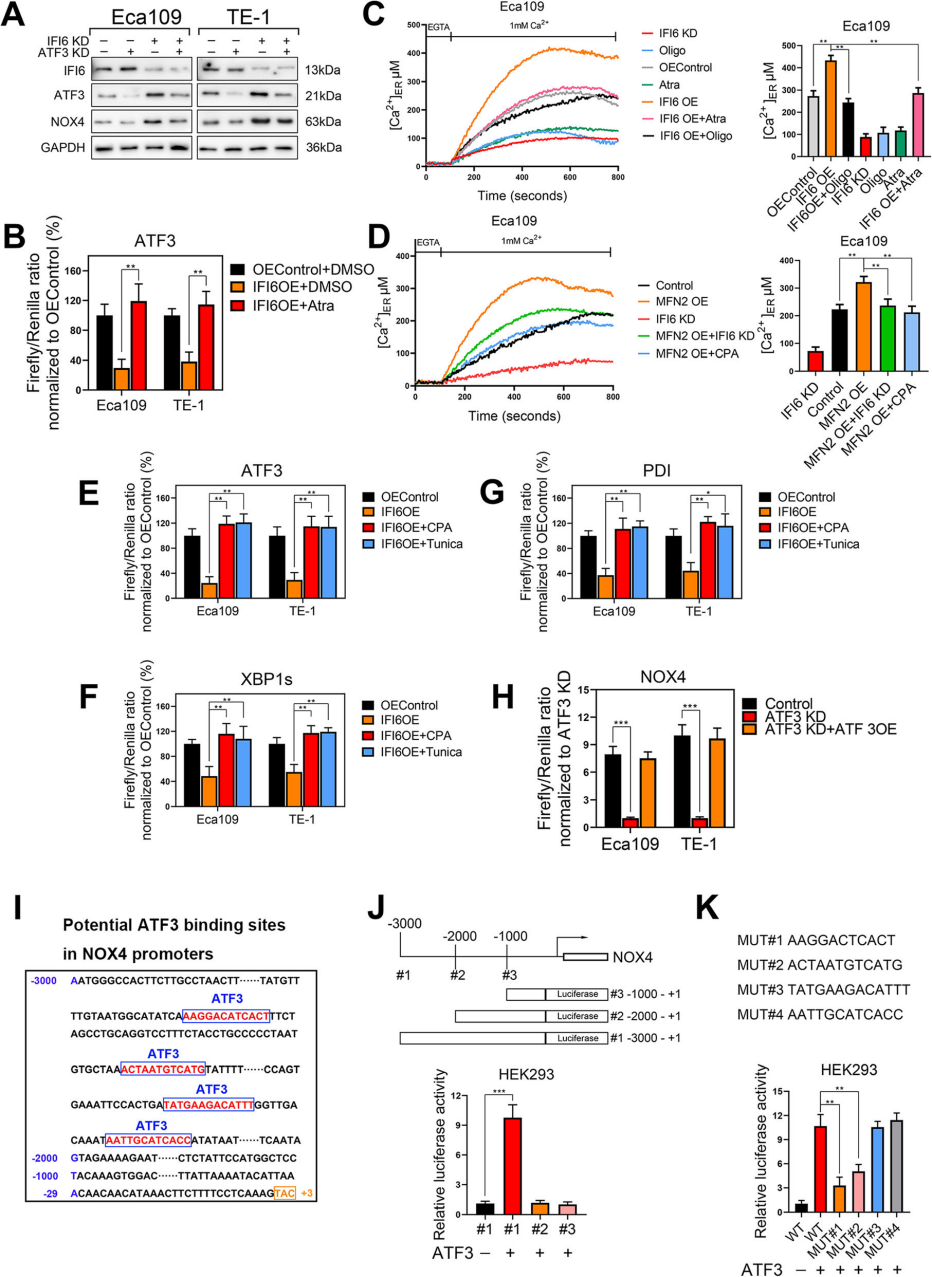
IFI6 silencing-induced ATP shortage activates ER stress by disrupting Ca^2+^ storage of ER and subsequently induces NOX4 up-regulation in an ATF3-dependent manner. **a**. Protein lysates were collected from the indicated Eca109 and TE-1 cells and subjected to immunoblotting to assess the expression of IFI6, ATF3 and NOX4. GAPDH was used as the loading control. **b.** ATF3 transcriptional activity was measured via a dual luciferase reporter assay in indicated ESCC cells. The data are presented as the means and SDs (n = 3). Statistical significance was determined by two-tailed Student’s t-test. ***P < 0.005. **c-d**. Representation (left) and statistical analysis (right) of the Endoplasmic reticulum Ca^2+^ in the indicated ESCC cells. Endoplasmic reticulum-targeted aequorin was exploited to monitor dynamic changes in free Ca^2+^ concentration in ER. The fluorescence intensity at each time point was recorded with an integrated spectrofluorometer. The data are presented as the means and SDs (n = 3). Statistical significance was determined by two-tailed Student’s t-test. **P < 0.01. **e-g**. Transcriptional activity of ATF3 (**e**), XBP1s (**f**) and PDI (**g**) was measured via a dual luciferase reporter assay in indicated ESCC cells. The data are presented as the means and SDs (n = 3). Statistical significance was determined by two-tailed Student’s t-test. ***P < 0.005. **h**. A dual luciferase reporter assay was used to determine NOX4 transcriptional activity. The relative luciferase activity was normalized to that in ATF3 KD ESCC cells. The data are presented as the means and SDs (n = 3). Statistical significance was determined by two-tailed Student’s t-test. ***P < 0.005. **i**. Schematic of potential ATF3 binding sites in the NOX4 promoter, as predicted by JASPAR. **j**. HEK293T cells were co-transfected with plasmids containing different NOX4 promoter constructs with or without the ATF3 expression plasmid. NOX4 transcriptional activity was measured via a dual luciferase reporter assay. The data are presented as the means and SDs (n = 3). Statistical significance was determined by two-tailed Student’s t-test. ***P < 0.005. **k**. A dual luciferase reporter assay was used to assess NOX4 transcriptional activity in HEK293T cells cotransfected with pGL3-NOX4-WT, pGL3-NOX4-Mut #1 (AAGGACTCACT), pGL3-NOX4-Mut #2 (ACTAATGTCATG), pGL3-NOX4-Mut #3 (TATGAAGACATTT) or pGL3-NOX4-Mut #4 (AATTGCATCACC) constructs with or without the ATF3 expression plasmid. The data are presented as the means and SDs (n = 3). Statistical significance was determined by two-tailed Student’s t-test. **P < 0.01

IFI6 silencing induces ER stress and ATF3 activation by disrupting Ca^2+^ storage of ER through ER Ca^2+^-ATPase pump.-To connect our observations related to ER stress and NOX4 elevation, we specifically downregulated ATF3 expression via shRNA in Eca109 and TE-1 cells. Subsequently, cell lysates were subjected to immunoblotting to assess the expression of ATF3 and NOX4 after IFI6 silencing. As predicted, ATF3 silencing inhibited the increase in NOX4 expression induced by IFI6 silencing, indicating that ATF3 may be the predominant transcriptional regulator of NOX4 upon alteration of IFI6 expression (Fig. 9a).

We next explored the molecular mechanism could be responsible for the regulation of ER stress and subsequently ATF3 activation following IFI6 alteration. For this, we constructed plasmids containing ATF3 promoter fragments and transfected them into ESCC cells with or without IFI6 overexpression. As expected, upregulation of IFI6 significantly inhibited ATF3 expression. However, atractyloside (Atra, 2 μM), an inhibitor of the adenine nucleotide transporter (ANT) that exports synthetized ATP from the mitochondrial matrix, fully reversed the effect of IFI6 overexpression on ATF3 transcriptional suppression even when OXPHOS was greatly enhanced following IFI6 upregulation (Fig. 9b). As reported, mitochondrial ATP pools is crucial for the function of the sarcoplasmic/endoplasmic reticulum Ca^2+^-ATPase (SERCA), the activity of which was shown to be inhibited following decreased mitochondrial ATP located in the interstitial spaces of mitochondria-associated membranes (MAMs) [37, 38]. Consistent with previous study, IFI6 downregulation caused a reduced ER Ca^2+^ levels, whereas IFI6 overexpression in ESCC cells significantly elevated Ca^2+^ levels in ER, which was reversed by treatment with compounds able to reduce mitochondrial ATP production (Oligomycin, 1 μM) or its export to the cytosol (Atra, 1 μM) (Fig. 9c, Figure S7A). Considering the crucial inter-talk between mitochondria and ER in Ca^2+^ signaling and the fact that decreased Ca^2+^ storage in ER may lead to ER stress [39–41], we further explored the implication of MAMs in IFI6-mediated, ATP deprivation-induced Ca^2+^ dynamic change of ER. We overexpressed Mitofusin2 (MFN2) in ESCC cells as MFN2 was known to enhance the number of endoplasmic reticulum-mitochondria contact sites [42]. As shown in Fig. 9d and S7B, IFI6 knockdown decreased ER Ca^2+^ uptake, which was reversed by MFN2 upregulation. Remarkably, ER Ca^2+^ concentration was consistently upregulated by MFN2 overexpression while cyclopiazonic acid (CPA, 10 μM), a specific SERCA inhibitor [43], was able to attenuate the effect of MFN2 overexpression on ER Ca^2+^ levels. These data indicated that IFI6 regulated ER Ca^2+^ stores through regulating SERCA pump activity possibly by affecting mitochondrial ATP abundance in MAMs. Ultimately, the dual-luciferase assays demonstrated that the classical endoplasmic reticulum stress-related events as upregulation of ATF3, XBP1s and PDI transcriptional activity following IFI6 overexpression were all blocked either by addition of CPA or supplement of Tunicamycin (Tunica, 0.5 mg/mL), an ER stress inducer (Fig. 9e-g). Taken together, we identified an IFI6-ATF3 signaling pathway whereby a reduced mitochondrial ATP levels subsequently suppressed Ca^2+^ uptake into ER, resulting in ER stress after IFI6 ablation.

### IFI6 silencing promotes the expression of NOX4 through transcriptional activation by ER stress effector ATF3

Finally, to decipher the molecular mechanism underlying the regulation of NOX4 by ATF3, we used a dual luciferase assay to investigate whether ATF3 regulated NOX4 transcriptional activity. As shown in Fig. 9h, knockdown of ATF3 in ESCC cells inhibited NOX4 promoter activity, while ectopic expression of ATF3 restored the transcriptional activity of NOX4.

Moreover, we performed a screen through JASPAR (http://jaspardev.genereg.net) to determine whether ATF3 could bind the NOX4 promoter and found that the NOX4 promoter region contains 4 potential ATF3 binding sites (Fig. 9i). To further validate whether ATF3 directly targets the NOX4 promoter, we constructed plasmids containing NOX4 promoter fragments of different lengths and transfected them into HEK293T cells with or without the ATF3 plasmid. As shown in Fig. 9j, NOX4 transcriptional activity was low in the absence of ATF3 expression. However, after ATF3 expression, the NOX4 promoter activity was substantially increased in HEK293T cells transfected with the full-length NOX4 promoter but not in cells transfected with the vectors containing bp − 2000 to bp + 1 or bp − 1000 to bp + 1, implying that the bp − 3000 to bp − 2000 region of the NOX4 promoter is responsible for the ATF3-induced transcriptional activity. Consistent with the above bioinformatic analysis results, this region contained all potential ATF3 binding sites, as illustrated in Fig. 8k. Moreover, mutation of either region #1 (AAGGACTCACT) or region #2 (ACTAATGTCATG) markedly suppressed ATF3-induced transcriptional activation of NOX4 (Fig. 9k).

Collectively, all these data indicate that interference with OXPHOS and the ETC through IFI6 downregulation leads to energy deprivation and subsequent ER stress, which in turn induces cellular ROS elevation through transcriptional activation of NOX4 via ATF3.

### IFI6 promotes ESCC growth in a xenograft model

**Fig. 10:**
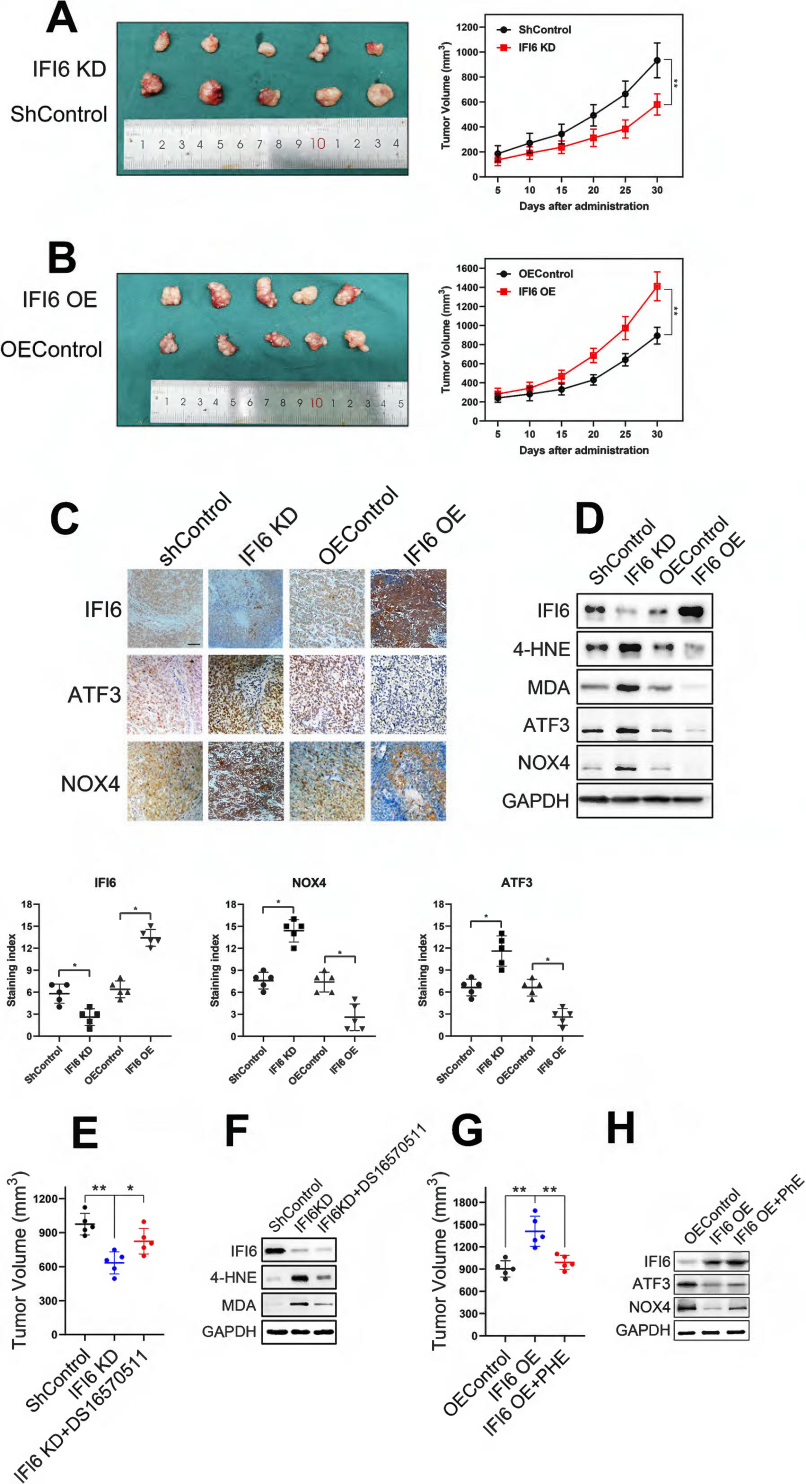
IFI6 promotes the growth of xenograft tumors from Eca109 cells. **a**-**b**. Representative image of fresh tumor tissues and tumor size quantification results in the xenograft model. Athymic nude mice were inoculated with the indicated Eca109 cells, and the tumor volumes (mm^3^) were calculated every 5 days during implantation. The data are presented as the means and SDs (*n* = 5). Statistical significance was determined by two-tailed Student’s t-test. **P < 0.01. **c**. Representative image (upper) and quantitative results (bottom) of the IHC measuring the abundance of IFI6, ATF3 and NOX4 in indicated tumor tissues derived from xenograft model. Magnification: 40×. Scale bar: 50 μm. Statistical significance was determined by Mann-Whitney test. *P < 0.5. **d**. Representative immunoblot showing IFI6, ATF3, NOX4 and ROS markers in indicated tumor tissues derived from xenograft model. GAPDH was used as the loading control. **e.** Tumor size quantification results in the xenograft model. The data are presented as the means and SDs (n = 5). Statistical significance was determined by two-tailed Student’s t-test. **P < 0.01. **f**. Representative immunoblot showing IFI6 and ROS markers in indicated tumor tissues derived from xenograft model. GAPDH was used as the loading control. **g**. Tumor size quantification results in the xenograft model. The data are presented as the means and SDs (n = 5). Statistical significance was determined by two-tailed Student’s t-test. **P < 0.01. **h**. Representative immunoblot showing IFI6, ATF3 and NOX4 in indicated tumor tissues derived from xenograft model. GAPDH was used as the loading control

Given that IFI6 elevation was implicated in the acceleration of ESCC cell growth in vitro, we further sought to determine whether altered IFI6 expression was sufficient to influence the proliferation of ESCC cells in vivo. For this purpose, we implanted parental Eca109 cells, IFI6-expressing Eca109 cells and IFI6KD Eca109 cells into the flanks of female nude mice. Notably, tumor growth was significantly inhibited following IFI6 knockdown, whereas upregulation of IFI6 produced the opposite effect (Fig. 10a, b). IHC was used to validate the abundance of IFI6, ATF3 and NOX4 in tumor tissues derived from xenograft model(Fig. 10c). Furthermore, we detected the protein levels of ROS markers (Malondialdehyde, MDA; 4-hydroxynonenal, 4-HNE), ATF3 and NOX4 in the primary tumors by Western blotting. As shown in Fig. 10d, ROS production and ATF3/NOX4 axis was upregulated in primary tumors derived from IFI6-knockdown cells than tumors derived from control cells. Conversely, IFI6 overexpression markedly decreased oxidative stress and suppressed ATF3/NOX4 signaling pathway, further confirmed IFI6-mediated ER stress in an in vivo model.

We further explored whether inhibition of MCU in xenograft model produced similar result as in vitro study. We observed that IFI6 knockdown compromised the growth of primary tumors in xenograft model, and this effect was partially rescued by DS16570511 administration (Fig. 10e). Moreover, the elevation of ROS markers in primary tumors derived from IFI6-knockdown was partially reversed after MCU inhibition.

Finally, phenformin (PHE), a well-known oxidative phosphorylation inhibitor, was administered to mice intraperitoneally every other day to evaluate the effect of OXPHOS inhibition after IFI6 overexpression. As expected, IFI6 overexpression promoted ESCC cells proliferation, while this effect was completely reversed after PHE treatment (Fig. 10g). Importantly, immunoblot from tumor tissues demonstrated that the suppression of ATF3/NOX4 axis in IFI6-overexpressed cells while this effect was reversed by phenformin treatment (Fig. 10h), validating the effect of IFI6-mediated OXPHOS efficiency on ATF3/NOX signaling pathway.

Thus, these xenograft models demonstrated that IFI6 promotes ESCC tumor growth in vivo, and again demonstrated a critical role of IFI6 in mitochondrial Ca^2+^, OXPHOS efficiency and ATF3/NOX4 axis.

## Discussion

The current study initially aimed to unambiguously determine the role of IFI6 in ESCC. Here, we identified that IFI6 was overexpressed in clinical ESCC samples and cell lines. Cancer cell-based experiments demonstrated that the activity of IFI6 controls the growth and survival of ESCC cells through the modulation of cellular ROS production. Mechanistically, we showed that IFI6 ablation inhibited OXPHOS efficiency and mitochondrial supercomplex assembly, which appeared to contribute to the decreased mitochondrial ROS generation in ESCC cells. Furthermore, the oxidative stress induced after IFI6 downregulation enhanced Tg-induced mitochondrial Ca^2+^ uptake, which in turn led to mitochondrial calcium overload and partially promoted the accumulation of mitochondrial ROS. In addition, our rescue experiments showed that IFI6 silencing elevated ER-derived ROS accumulation by driving ER stress accompanied by a substantial increase in ATF3 expression and subsequent transcriptional activation of NOX4. We also confirmed that the function of SERCA, an ER located ATP-dependent Ca^2+^ pump, was modulated by ATP production and mitochondrial OXPHOS efficiency resulting from altered expression of IFI6. The induction of ER stress and concomitant upregulation of ATF3 following IFI6 silencing is possibly mediated by the decreased ER Ca^2+^ pools due to the reduced activity of SERCA. Finally, via knockdown and overexpression experiments, we validated the antiproliferative effect of IFI6 depletion in a nude mouse model of ESCC. Collectively, these observations imply the potential therapeutic value of IFI6 inhibition in ESCC.

Our analysis of IFI6 expression in a panel of ESCC cell lines and patient samples indicated that a high abundance of IFI6 might be correlated with biological aggressiveness in ESCC. While ESCC is notorious for its heterogeneity, our research indicated the existence of an IFI6-positive patient subgroup, which was notably predominant among the cohort with ESCC at more advanced stages. These findings were supported by analysis of data in public databases, including TCGA and GEO. Furthermore, consistent with our results, IFI6 overexpression is implicated in multiple malignant diseases [22, 44, 45], and the increase in ATF3 expression was associated with a favorable prognosis in patients with ESCC [46, 47].

IFI6 silencing promoted ROS production, consistent with previous findings by another group. However, our functional study did not show an overt IFI6-mediated change in metastasis potential, as previously shown for breast cancer [24]. In addition, the molecular mechanism underlying IFI6-mediated mitochondrial ROS production remains elusive, prompting us to further investigate the exact role of IFI6 in ESCC.

Previous reports indicate that mitochondrial supercomplex assembly modulates the generation of mitochondrial ROS, which is produced principally by OXPHOS [31]. As reported, the substantial accumulation of mitochondrial ROS can be induced by the loss of supercomplex organization [48, 49]. Jang and Javadov recently showed that complex I and II subunit depletion elevated mitochondrial ROS production in the heart but impaired respirasome formation and ATP production [32]. A As altered IFI6 expression can alter cellular ATP production and OXPHOS efficiency, we hypothesized that the decreased mitochondrial supercomplex assembly following IFI6 silencing may enhance mitochondrial ROS production, reflecting the efficiency of the OXPHOS complex. Although IFI6 is not a component of the ETC, our research provides evidence that IFI6 may act as a bona fide regulator of mitochondrial supercomplex formation. Consistent with this hypothesis, IFI6 depletion decreased the content of complexes I, III and IV in the mitochondrial supercomplex, while the expression of the individual respiratory complexes did not change. In contrast, overexpression of IFI6 effectively enhanced mitochondrial supercomplex formation. Although the exact mechanism underlying IFI6-mediated mitochondrial supercomplex assembly remains to be explored, we hypothesized that IFI6 might facilitate mitochondrial supercomplex assembly directly by interacting with respiratory complex subunits or indirectly by enhancing weak interactions between mitochondrial complexes.

Previous studies have shown that mitochondria can take up calcium from the cytosol (through ORAI channels) or via the ER (through channels ranging from IP_3_R to ER calcium leak channels) [50, 51]. As calcium influx into mitochondria leads to the induction of programmed cell death [52], we assumed that dysregulated mitochondrial calcium dynamics could lead to mitochondrial dysfunction, contribute to ROS production, and ultimately induce cell apoptosis following IFI6 depletion. Indeed, we observed a significant increase in the Tg-induced mitochondrial Ca^2+^ uptake after IFI6 depletion. Furthermore, addition of the calcium chelator BAPTA or removal of calcium from the culture medium reduced mitochondrial ROS generation upon IFI6 silencing. Calcium released from the ER and that present in the cytosol is transported into mitochondria via VDAC1, MCU, and NCLX [53–56]; however, our expression analysis revealed no overt alterations in these components of the mitochondrial calcium uptake machinery upon IFI6 knockdown. Mitochondrial oxidative stress and calcium signaling are two functional entities that often coexist and are interconnected to maintain appropriate cellular physiology [57, 58]. Moreover, Dong et al. showed that the redox regulation of MCU plays a role in mitochondrial calcium dynamics and oxidation; oxidation of MCU facilitated higher-order MCU oligomer formation and increased the MCU calcium uptake rate, mitochondrial ROS accumulation, and calcium overload-induced cell death [59]. Consistent with this observation, pharmacological inhibition of MCU with DS16570511 partially prevented IFI6 silencing-induced mitochondrial ROS elevation. Taken together, these data suggest that the insufficient OXPHOS resulting from IFI6 silencing causes the accumulation of excess mitochondrial ROS, which promotes the oxidation of MCU, which in turns leads to increased MCU Ca^2+^ uptake and concomitant mitochondrial Ca^2+^ overload, disruption of mitochondrial Ca^2+^ dynamics further adds to ROS production. In this process, disrupted respiratory complexes formation and OXPHOS insufficiency are the leading and primary causes, while concomitant mitochondrial Ca^2+^ overload is the secondary cause.

We could not rule out the possibility that other sources of ROS contribute to the oxidative stress generated upon IFI6 downregulation. The ER is a large membrane-like organelle with various important cellular functions, ranging from maintaining proper protein folding to modulating calcium homeostasis to hosting components of intracellular signaling pathways [60, 61]. The unfolded protein response (UPR), specifically activated in response to ER stress, can be induced by multiple factors, including nutrient deficiency, and produces endogenous or exogeneous damage to cellular functions, leading to impaired intracellular calcium dynamics and redox homeostasis [62, 63]. Here, we demonstrated that IFI6 depletion led to induction of ER stress possibly mediated by an ATP shortage through interference with mitochondrial OXPHOS. Moreover, a reduction in the activity of SERCA upon decreased ATP supplied by mitochondria leads to ER stress, which is consistent with previous study [64]. In our experiment, we found that ER Ca^2+^ was significantly decreased even when IFI6 was upregulated, but ATP export from mitochondrial was blocked using Atra. On the other hand, overexpressing MFN2 promoted ER Ca^2+^ uptake by increasing mitochondrial-ER contacting sites. Our rescue experiments demonstrated that pharmaceutical inhibition of SERCA could block the effect of IFI6 overexpression upon ER stress suppression, this phenomenon was also mimicked by ER stress inducer tunica, which further corroborate our hypothesis. However, additional studies were necessary to decipher how ER Ca^2+^ depletion mediated by IFI6 silencing contributes to ER stress and especially, the transcriptional activation of ATF3. We also revealed that NOX4, which was identified as a key player in cellular ROS production following IFI6 knockdown, was regulated by ER stress and the associated transcription factor ATF3. These findings ultimately suggest that IFI6 may serve as a cellular antioxidant in ESCC by suppressing NOX4.

Finally, we demonstrated that IFI6 exerts pro-carcinogenic activity in vivo in an ESCC xenograft model in nude mice. These observations further confirm the in vitro findings and suggest that the specific inhibition of IFI6 activity might have implications for patients with ESCC. Several studies report that antioxidants can promote aggressive behavior and drug resistance in ESCC [65–67]. Our findings further highlight the importance of better understanding redox homeostasis in malignant diseases and the possible clinical applicability of IFI6.

**Fig. 11:**
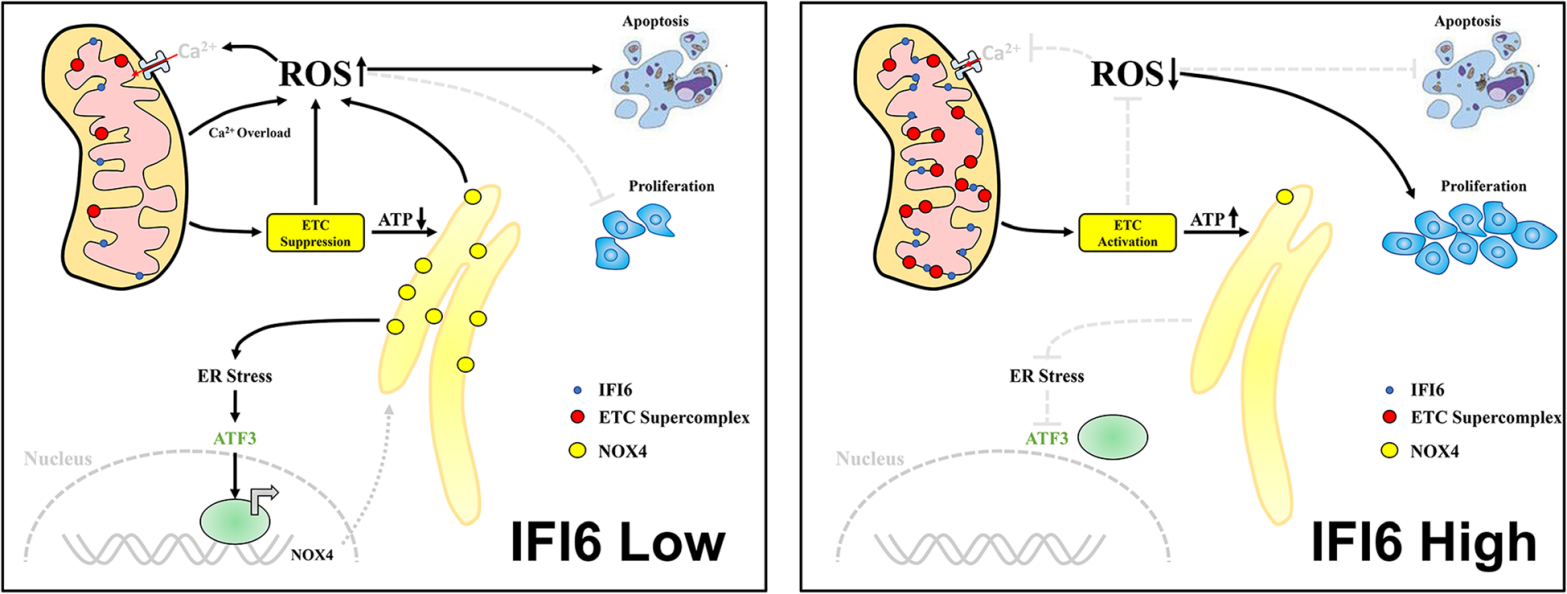
Schematic of IFI6-modulated ROS generation in ESCC cells. (Left panel) In IFI6_low_ ESCC cells, mitochondrial supercomplex formation is suppressed, leading to OXPHOS deficiency and subsequent mitochondrial ROS overproduction, which in turn causes mitochondrial calcium overload. The ATP shortage resulting from insufficient OXPHOS induces ER stress, leading to the upregulation of ATF3 expression and the transcriptional activation of NOX4, which enhances ER-mediated ROS production. All of these mechanisms ultimately cause oxidative stress. Elevated ROS levels suppress tumor cell proliferation and induce apoptosis. (Right panel) In IFI6^high^ ESCC cells, undisturbed mitochondrial supercomplex assembly allows for stable mitochondrial calcium influx, optimal mitochondrial function, and inhibition of ER stress. Under these conditions, mitochondria and NOX4 produce low or moderate levels of ROS, which can be eliminated by cellular antioxidants, ultimately promoting cancer cell proliferation and suppressing cell apoptosis

In summary, we identify what we believe to be a new IFI6-mediated pathway, manifest its hyperactivation in ESCC. Mechanically, inhibition of IFI6 suppresses mitochondrial supercomplex formation, which leads to OXPHOS deficiency and concomitant mitochondrial ROS overproduction. Excess ROS causes mitochondrial calcium overload, which in its turn further adds to mitochondrial ROS generation. The ATP shortage resulting from insufficient OXPHOS induces ER stress due to a decrease in SERCA function, leading to the upregulation of ATF3 expression and the transcriptional activation of NOX4, which enhances ER-mediated ROS production. All of these mechanisms ultimately cause oxidative stress. This circuit is illustrated in Fig. 11.

## Conclusions

Taken together, our data indicated the carcinogenesis of IFI6 in ESCC and revealed that knockdown of IFI6 suppressed proliferation and induced apoptosis by increasing ROS accumulation. We further found that mitochondrial ROS accumulation is induced by the suppression of mitochondrial supercomplex assembly and mitochondrial calcium overload; IFI6 inhibition also upregulated NOX4-derived ROS production in an ATF3-dependent manner through ER stress induction. All these observations would implicate a novel therapeutic avenue for the treatment of ESCC.

## Supplementary information

**Additional file 1: Figure S1**. IFI6 expression in ESCC cells transfected with IFI6 construct or IFI6-ShRNA and its prognostic value in ESCC. A. Plots visualizing the Kaplan-Meier analysis and log-rank test for ESCC patients from the TCGA database separated into groups with high or low expression levels of IFI6. Statistical significance was determined via the log-rank test. B. mRNA levels of IFI6, as measured by qRT-PCR, in two ESCC cell lines after shRNA-mediated depletion of IFI6. Data were normalized to the expression of IFI6 in ShControl cells and are presented as the means and SDs (n=3). Statistical significance was determined by a two-tailed Student’s t-test. ***P<0.005. C. Representative immunoblot showing IFI6 protein levels in Eca109 and TE-1 cells after shRNA-mediated depletion of IFI6. GAPDH was used as the loading control. D-E. Eca109 or TE-1 cells were transfected with IFI6 or empty vector and selected in medium containing G418. qRT-PCR (D) and immunoblotting (E) were performed to validate the overexpression efficiency. GAPDH was used as the loading control. Data were normalized to the expression of IFI6 in OEControl cells and are presented as the means and SDs (n=3). Statistical significance was determined by a two-tailed Student’s t-test. ***P<0.005.

**Additional file 2: Figure S2**. IFI6 overexpression promotes cell proliferation, inhibits apoptosis and ameliorates oxidative stress in ESCC. A-B. Representative images (A) and statistical quantification (B) of EdU staining in ESCC cell lines transfected with IFI6-plasmic (IFI6OE) or empty vector (OEControl). EdU: red, Hoechst 33342: blue. The data are presented as the means and SDs (n=3). Scale bar: 20 μm. Statistical significance was determined by two-tailed Student’s t-test. ***P<0.005. C. Representative images (upper) and statistical quantification (lower) of apoptotic and necrotic cell populations in ESCC cell lines, as determined by Annexin-V FITC/PI staining and flow cytometry. Cells with a FITC^-^ and PI^-^ signature were considered viable. Cells with a FITC^+^ and PI^-^ or a FITC^+^ and PI^+^ signature were considered nonviable. The data are presented as the means and SDs (n=3). Statistical significance was determined by two-tailed Student’s t-test. **P<0.01. D. Representative images (upper) and statistical quantification (lower) of ROS production assay results in ESCC cells. The indicated cells were stained with carboxy-H_2_DCFDA and observed under a fluorescence microscope. H_2_DCFDA: green, Hoechst 33342: blue. Scale bar: 20 μm. The data are presented as the means and SDs (n=3). Statistical significance was determined by two-tailed Student’s t-test. **P<0.01.

**Additional file 3: Figure S3**. ROS accumulation is responsible for the IFI6 silencing-induced reduction in cell viability. A. Representative images (left) and statistical quantification (right) of EdU staining in the indicated TE-1 cells preincubated with different ROS inhibitors. EdU: red, Hoechst 33342: blue. Scale bar: 20 μm. The data are presented as the means and SDs (n=3). Statistical significance was determined by one-way ANOVA. ***P<0.005. B. Representative images (left) and statistical quantification (right) of the apoptosis assay results in TE-1 cells, as indicated by the mitochondrial membrane potential. The indicated cells were stained with JC-1 after preincubation with different ROS inhibitors. Cells stained with JC-1 are visible as either green (J-monomers) or red (J-aggregates) fluorescence. The apoptosis rate was calculated as the ratio of JC-1 aggregates to JC-1 monomers. Scale bar: 20 μm. The data are presented as the means and SDs (n=3). Statistical significance was determined by one-way ANOVA. ***P<0.005.

**Additional file 4: Figure S4**. The expression level of IFI6 does not affect the expression of individual respiratory complexes. A. Immunoblot of NCLX, VDAC1, MCU and GAPDH expression in ESCC cells with stable IFI6 knockdown. B. mRNA levels of NCLX, VDAC1 and MCU in the indicated ESCC cells as measured via qRT-PCR. The data are presented as the means and SDs (n=3).

**Additional file 5: Figure S5**. IFI6 modulates mitochondrial ATP production and the oxidative phosphorylation efficiency. A. Representative plots (upper) and quantitative results (bottom) of the cellular OCR, basal and maximal respiration rates in the different groups. The indicated ESCC cells were subjected to extracellular flux analysis in the Seahorse XF instrument. The arrows and dotted lines indicate the addition of Oligo (oligomycin) (1 μM), FCCP (Carbonyl cyanide 4-(trifluoromethoxy) phenylhydrazone) (0.5 μM) and Rot&AMA (Rotenone and Antimycin A) (0.5 μM each). The data are presented as the means and SDs (n=3). Statistical significance was determined by two-tailed Student’s t-test. **P<0.01. B. Representative plots (upper) and quantitative results (bottom) of the real-time ECAR, glycolysis and glycolytic capacity assays in the indicated ESCC cells. The ECAR was determined following sequential addition of glucose (10 mM), oligomycin (1 μM) and 2-DG (100 mM). Glycolysis was measured by subtracting the ECAR after glucose addition from the ECAR before glucose addition. The glycolytic capacity was calculated by subtracting the ECAR after oligomycin treatment from the ECAR before glucose addition. The data are presented as the means and SDs (n=3). Statistical significance was determined by a two-tailed Student’s t-test. C. Representative plots (upper) and quantitative results (bottom) of the complex I-dependent OCR in the different groups. Pyruvate (Pyr) (5 mM) and malate (Mat) (5 mM) were added to digitonin (Dig)-permeabilized cells, and the OCR was monitored. The data are presented as the means and SDs (n=3). Statistical significance was determined by two-tailed Student’s t-test. **P<0.01. D. Representative plots (upper) and quantitative results (bottom) of the complex III-dependent OCR in the different groups. Rotenone was added to digitonin-permeabilized cells to inhibit complex I, after which G3P (5 mM) was added, and the OCR was monitored. The data are presented as the means and SDs (n=3). Statistical significance was determined by two-tailed Student’s t-test. **P<0.01. E. Representative plots (upper) and quantitative results (bottom) of the complex II-, and complex IV-dependent OCRs in the different groups. Rotenone (1 μM) was added to inhibit complex I; succinate (Suc) (5 mM), Antimycin (AMA) (1 μM) and TMPD/ascorbate (500 μM and 5 mM, respectively) were then added to digitonin-permeabilized cells, and the OCR was monitored. The data are presented as the means and SDs (n=3). Statistical significance was determined by two-tailed Student’s t-test. **P<0.01.

**Additional file 6: Figure S6**. IFI6 modulates mitochondrial ROS production and OXPHOS efficiency by regulating mitochondrial supercomplex assembly. A. Mitochondrial proteins extracted from the indicated ESCC cells were solubilized with digitonin and subjected to BN-PAGE followed by immunoblotting. Mitochondrial supercomplexes were first visualized by incubation with antibodies against complex I (CI, NDUFB8), and the membrane was then stripped and reprobed with antibodies against complex III (CIII, RISP). The membrane was then sequentially probed with antibodies against complex IV (CIV, COXIV), complex II (CII, SDHA) and complex V (CV, ATPB). B-C. Quantitative results for experiments shown in A. Data are presented as mean and SD (n=3). Statistical significance was determined by two-tailed student’s t-test. **P<0.01.

**Additional file 7: Figure S7**. IFI6 modulates mitochondrial ROS production and OXPHOS efficiency by regulating mitochondrial supercomplex assembly. A. Representation (left) and statistical analysis (right) of the Endoplasmic reticulum Ca^2+^ in the indicated ESCC cells. Endoplasmic reticulum-targeted aequorin was exploited to monitor dynamic changes in free Ca^2+^ concentration in ER. The fluorescence intensity at each time point was recorded with an integrated spectrofluoromete. The data are presented as the means and SDs (n=3). Statistical significance was determined by two-tailed Student’s t-test. **P<0.01. B. Representation (left) and statistical analysis (right) of the Endoplasmic reticulum Ca^2+^ in the indicated ESCC cells. Endoplasmic reticulum-targeted aequorin was exploited to monitor dynamic changes in free Ca^2+^ concentration in ER. The fluorescence intensity at each time point was recorded with an integrated spectrofluoromete. The data are presented as the means and SDs (n=3). Statistical significance was determined by two-tailed Student’s t-test. **P<0.01.

**Additional file 8: Table S1**. Association between IFI6 protein levels and several clinical characteristics in 83 cases of ESCC in the immunohistochemistry cohort.

**Additional file 9: Table S2**. Univariate and Multivariate Cox regression analysis for overall survival in 83 ESCC patients from immunohistochemistry cohort.

**Additional file 10: Table S3.** Clinicopathological features of ESCC patients in the qRT-PCR cohort.

**Additional file 11: Table S4.** Primers used for quantitative real-time PCR.

**Additional file 12: Table S5**. Oligonucleotides used for silencing the expression of target genes.

**Additional file 13: Table S6.** The 167 mRNAs predicted to be coexpressed with IFI6 in all four GEO datasets.

## Data Availability

All data from this study can be requested directly from the corresponding author upon reasonable request.

## Abbreviations

ESCC: Esophageal squamous cell carcinoma
IFI6: Interferon alpha-inducible protein 6
ROS: Reactive oxygen species
OXPHOS: Oxidative phosphorylation
ER: Endoplasmic reticulum
GEO: Gene Expression Omnibus
IHC: Immunohistochemistry
TCGA: The Cancer Genome Atlas
GO: Gene Ontology
BP: Biological Process
GSEA: Gene set enrichment analysis
DDT: Dithiothreitol
CAT: PEG-catalase
NAC: N-acetyl-L-cysteine
mtROS: Mitochondrial ROS
Tg: Thapsigargin
SOCE: Store-operated Ca^2+^ entry
VDAC1: Voltage-dependent anion channel type 1
MCU: Mitochondrial Ca^2+^ uniporter
NCLX: Mitochondrial sodium calcium lithium exchanger
Oligo: Oligomycin
FCCP: Carbonyl cyanide 4-(trifluoromethoxy) phenylhydrazone
Rot: Rotenone
AMA: Antimycin A
G3P: Glyceraldehyde 3-phosphate
TMPD: N,N,N′,N′-Tetramethyl-p-phenylenediamine
2-DG: 2-deoxy-D-glucose
BN-PAGE: Blue native polyacrylamide gel electrophoresis
NOXs: NADPH oxidases
XBP1s: X-box binding protein 1
PDI: Protein disulfide isomerase
BiP: Binding immunoglobulin protein
RSCs: Respiratory supercomplexes
Atra: Atractyloside
SERCA: Sarcoplasmic/endoplasmic reticulum Ca^2+^-ATPase
PHE: Phenformin
CPA: Cyclopiazonic acid
Tunica: Tunicamycin
MDA: Malondialdehyde
4-HNE: 4-hydroxynonenal
