# Correction: IFI6 depletion inhibits esophageal squamous cell carcinoma progression through reactive oxygen species accumulation via mitochondrial dysfunction and endoplasmic reticulum stress

**DOI:** 10.1186/s13046-024-03256-9

**Published:** 2025-01-04

**Authors:** Zhenchuan Liu, Shaorui Gu, Tiancheng Lu, Kaiqing Wu, Lei Li, Chenglai Dong, Yongxin Zhou

**Affiliations:** Department of Thoracic Surgery, Shanghai Tongji Hospital Affiliated With Tongji University, Shanghai, 200065 P. R. China

**Correction** **:** **J Exp Clin Cancer Res 39, 144 (2020)**

**https://doi.org/10.1186/s13046-020-01646-3**

Following the publication of the original article [1], the authors identified errors in the images of Fig. 3, Fig. 5, Fig. 8, Fig. 10 and Supplementary Material Table S4, specifically:

- Fig.3D - incorrect image for Eca109 with IFI6-KD
- Fig.5G - incorrect fluorescence data for TE-1 cells
- Fig.8A - incorrect fluorescence image for Eca109
- Fig.8G - incorrect band of GAPDH for TE-1 cells
- Fig.10C - incorrect representative image for IFI6 staining in the OEControl and IFI6-OE groups were included
- Table S4 - Incorrect primer sequences for MCU, ATF3 and NCLX were uploaded

The corrected figures are provided below:

The corrections do not affect the overall results, discussion, or conclusion of the article.

**Incorrect Figure 3**

**Fig. 3:**
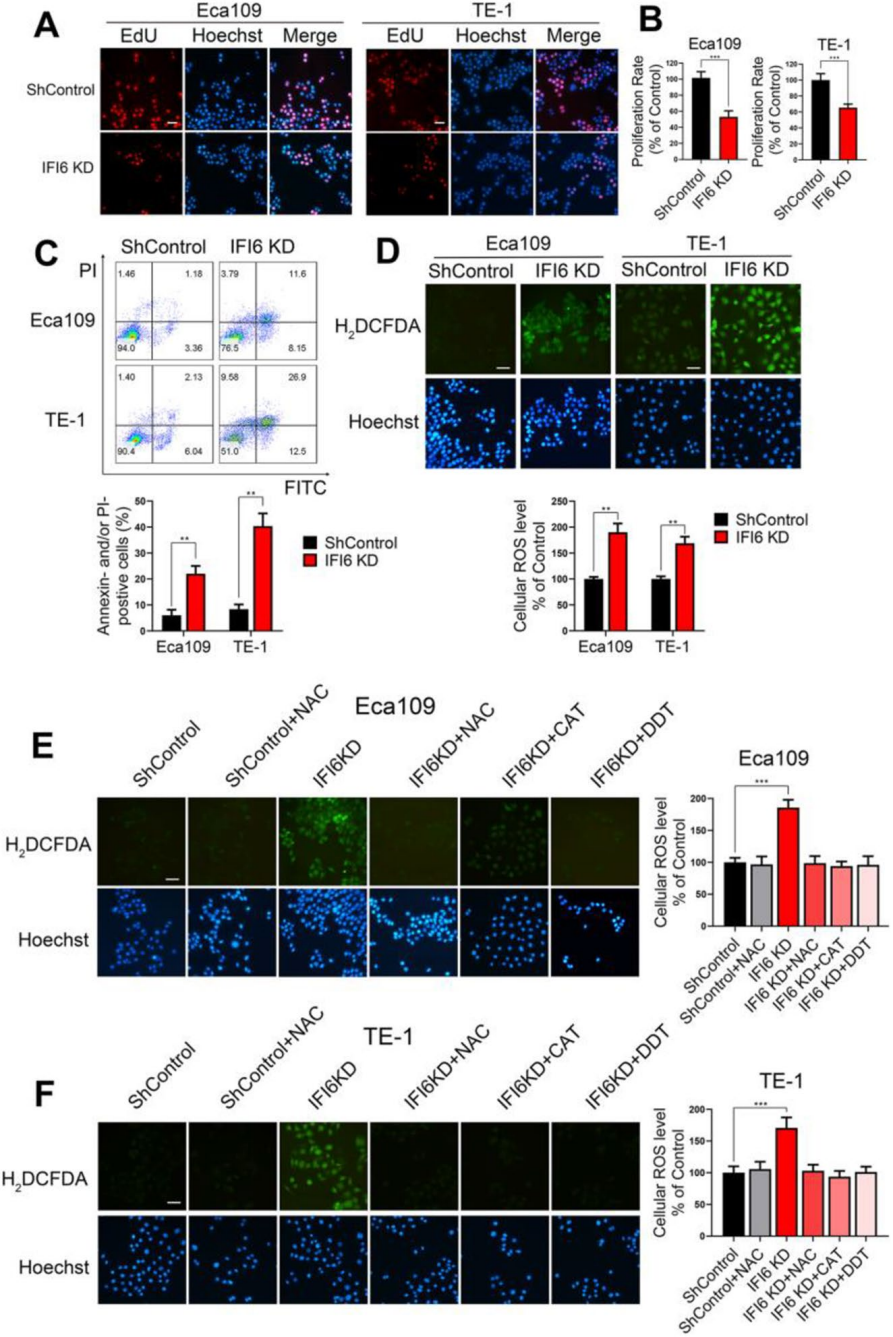
IFI6 promotes cell proliferation, inhibits apoptosis and ameliorates oxidative stress in ESCC. **a-b**. Representative images (**a**) and statistical quantification (**b**) of EdU staining in ESCC cell lines transfected with IFI6-shRNA or ShControl lentivirus. EdU: red, Hoechst 33342: blue. The data are presented as the means and SDs (*n* = 3). Scale bar: 20 μm. Statistical significance was determined by two-tailed Student’s t-test. ***P < 0.005. **c**. Representative images (upper) and statistical quantification (lower) of apoptotic and necrotic cell populations in ESCC cell lines, as determined by Annexin-V FITC/PI staining and flow cytometry. Cells with a FITC− and PI− signature were considered viable. Cells with a FITC+ and PI− or a FITC+ and PI+ signature were considered nonviable. The data are presented as the means and SDs (*n* = 3). Statistical significance was determined by two-tailed Student’s t-test. **P < 0.01. **d**. Representative images (upper) and statistical quantification (lower) of ROS production assay results in ESCC cells. The indicated cells were stained with carboxy-H2DCFDA and observed under a fluorescence microscope. H2DCFDA: green, Hoechst 33342: blue. Scale bar: 20 μm. The data are presented as the means and SDs (*n* = 3). Statistical significance was determined by two-tailed Student’s t-test. **P < 0.01. **e-f**. Representative images (left) and statistical quantification (right) of ROS production assay results in Eca109 (**e**) and TE-1 (**f**). The indicated cells were preincubated with different ROS inhibitors, stained with carboxy-H2DCFDA and observed under a fluorescence microscope. H2DCFDA: green, Hoechst: blue. Scale bar: 20 μm. The data are presented as the means and SDs (*n* = 3). Statistical significance was determined by one-way ANOVA. **P < 0.01

**Correct Figure 3**

**Fig. 3:**
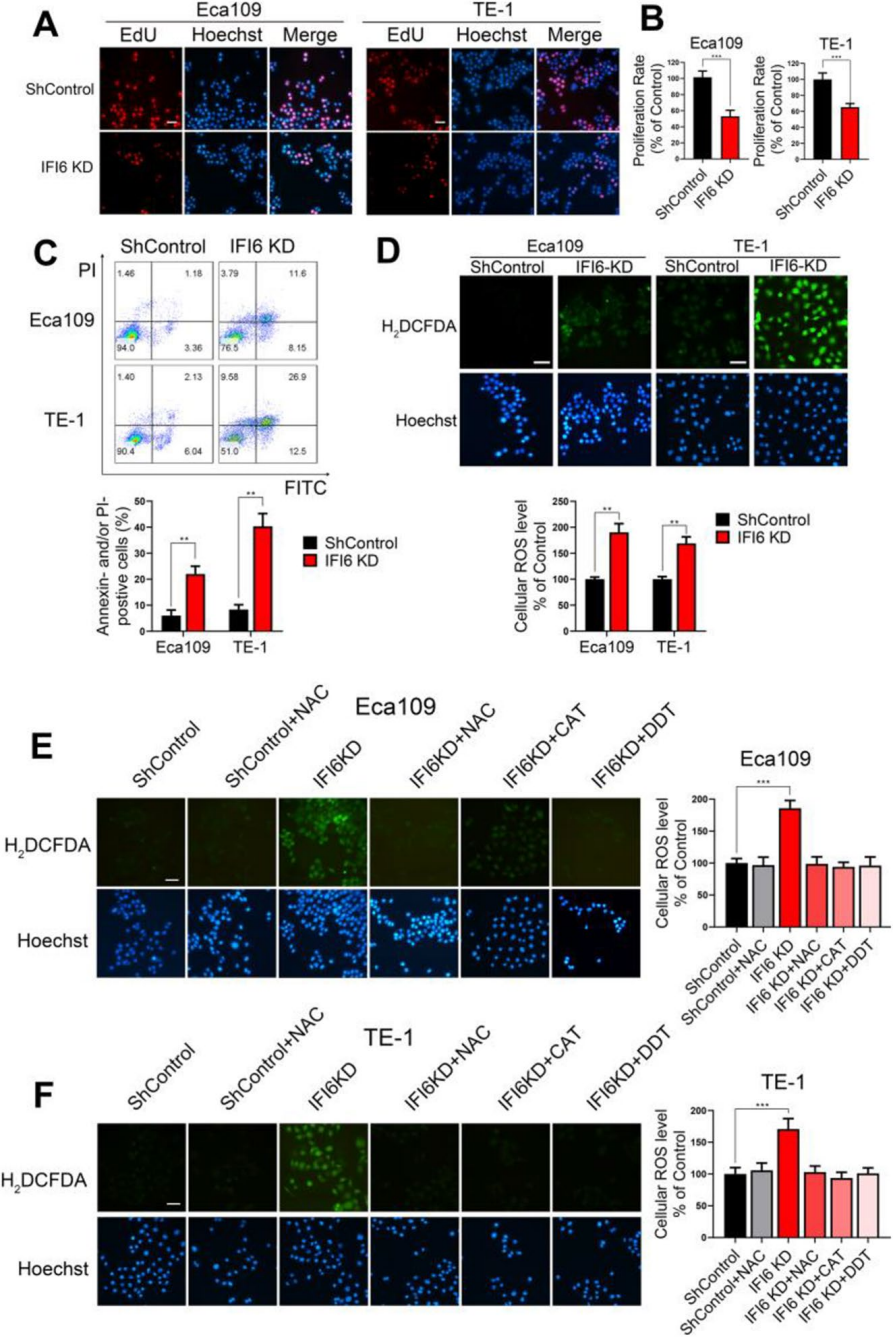
IFI6 promotes cell proliferation, inhibits apoptosis and ameliorates oxidative stress in ESCC. **a-b**. Representative images (**a**) and statistical quantification (**b**) of EdU staining in ESCC cell lines transfected with IFI6-shRNA or ShControl lentivirus. EdU: red, Hoechst 33342: blue. The data are presented as the means and SDs (*n* = 3). Scale bar: 20 μm. Statistical significance was determined by two-tailed Student’s t-test. ***P < 0.005. **c**. Representative images (upper) and statistical quantification (lower) of apoptotic and necrotic cell populations in ESCC cell lines, as determined by Annexin-V FITC/PI staining and flow cytometry. Cells with a FITC− and PI− signature were considered viable. Cells with a FITC+ and PI− or a FITC+ and PI+ signature were considered nonviable. The data are presented as the means and SDs (*n* = 3). Statistical significance was determined by two-tailed Student’s t-test. **P < 0.01. **d**. Representative images (upper) and statistical quantification (lower) of ROS production assay results in ESCC cells. The indicated cells were stained with carboxy-H2DCFDA and observed under a fluorescence microscope. H2DCFDA: green, Hoechst 33342: blue. Scale bar: 20 μm. The data are presented as the means and SDs (*n* = 3). Statistical significance was determined by two-tailed Student’s t-test. **P < 0.01. **e-f**. Representative images (left) and statistical quantification (right) of ROS production assay results in Eca109 (**e**) and TE-1 (**f**). The indicated cells were preincubated with different ROS inhibitors, stained with carboxy-H2DCFDA and observed under a fluorescence microscope. H2DCFDA: green, Hoechst: blue. Scale bar: 20 μm. The data are presented as the means and SDs (*n* = 3). Statistical significance was determined by one-way ANOVA. **P < 0.01

**Incorrect Figure 5**

**Fig. 5:**
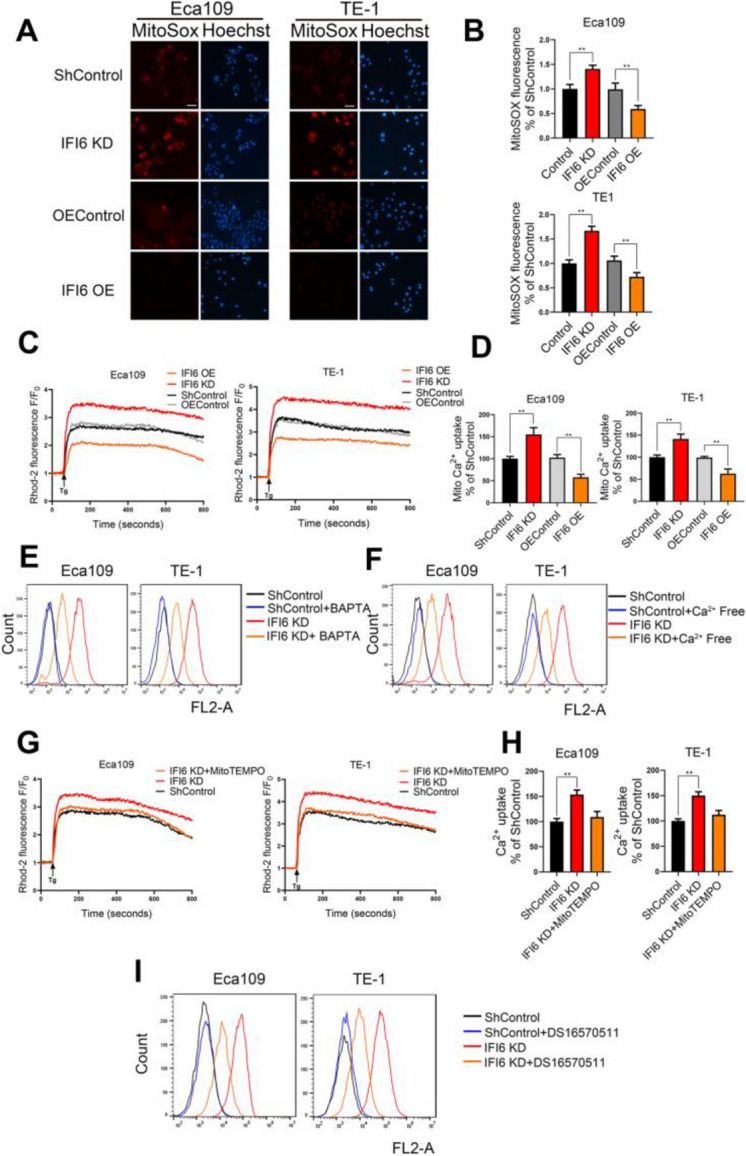
IFI6 modulates mitochondrial ROS production by regulating mitochondrial Ca^2+^ overload. **a-b**. Representative images (**a**) and statistical quantification (**b**) of mitochondrial ROS (mtROS) production assay results in ESCC cells. The indicated cells were stained with MitoSOX, and fluorescence was quantified under a fluorescence microscope. MitoSOX: red, Hoechst: blue. Scale bar: 20 μm. The data are presented as the means and SDs (*n* = 3). Statistical significance was determined by two-tailed Student’s t-test. **P < 0.01. c-d. Quantification (**c**) and statistical analysis (**d**) of the relative Rhod-2 AM fluorescence intensity time course in the indicated ESCC cells. The fluorescence intensity at each time point was recorded with an integrated spectrofluorometer at excitation and emission wavelengths of 550 nm and 580 nm, respectively. The relative fluorescence intensity was calculated as a percentage of the baseline fluorescence intensity, which was recorded during the first 60 s (F0). The arrow indicates the time at which Tg was added. The data are presented as the means and SDs (*n* = 3). Statistical significance was determined by two-tailed Student’s t-test. **P < 0.01. **e**. The indicated ESCC cells were incubated in the presence or absence of BAPTA-AM (2 μM) for 1 h, and mitochondrial ROS levels were then measured by MitoSOX staining followed by flow cytometry. **f**. The indicated ESCC cells were cultured in complete medium or calcium-deficient medium, and mitochondrial ROS levels were determined by MitoSOX staining followed by flow cytometry. **g-h**. Quantification (**g**) and statistical analysis (**h**) of the relative Rhod-2 AM fluorescence intensity time course in the indicated ESCC cells. The fluorescence intensity at each time point was recorded with an integrated spectrofluorometer at excitation and emission wavelengths of 550 nm and 580 nm, respectively. The relative fluorescence intensity was calculated as a percentage of the baseline fluorescence intensity, which was recorded during the first 60 s (F0). The arrow indicates the time at which Tg was added. The data are presented as the means and SDs (*n* = 3). Statistical significance was determined by one-way ANOVA. **P < 0.01. i. The indicated ESCC cells were incubated in the presence or absence of the MCU inhibitor DS16570511 (10 μM), and mitochondrial ROS levels were then measured by MitoSOX staining, followed by flow cytometry

**Correct Figure 5**

**Fig. 5:**
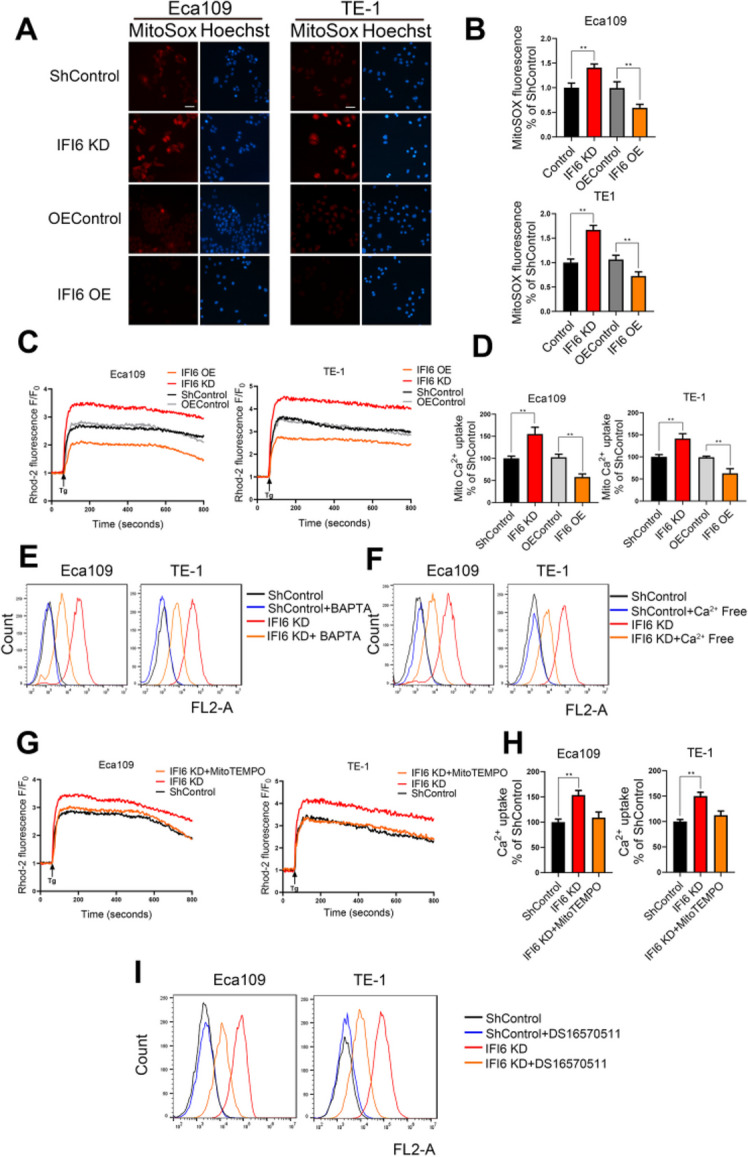
IFI6 modulates mitochondrial ROS production by regulating mitochondrial Ca^2+^ overload. **a-b**. Representative images (**a**) and statistical quantification (**b**) of mitochondrial ROS (mtROS) production assay results in ESCC cells. The indicated cells were stained with MitoSOX, and fluorescence was quantified under a fluorescence microscope. MitoSOX: red, Hoechst: blue. Scale bar: 20 μm. The data are presented as the means and SDs (*n* = 3). Statistical significance was determined by two-tailed Student’s t-test. **P < 0.01. c-d. Quantification (**c**) and statistical analysis (**d**) of the relative Rhod-2 AM fluorescence intensity time course in the indicated ESCC cells. The fluorescence intensity at each time point was recorded with an integrated spectrofluorometer at excitation and emission wavelengths of 550 nm and 580 nm, respectively. The relative fluorescence intensity was calculated as a percentage of the baseline fluorescence intensity, which was recorded during the first 60 s (F0). The arrow indicates the time at which Tg was added. The data are presented as the means and SDs (*n* = 3). Statistical significance was determined by two-tailed Student’s t-test. **P < 0.01. **e**. The indicated ESCC cells were incubated in the presence or absence of BAPTA-AM (2 μM) for 1 h, and mitochondrial ROS levels were then measured by MitoSOX staining followed by flow cytometry. **f**. The indicated ESCC cells were cultured in complete medium or calcium-deficient medium, and mitochondrial ROS levels were determined by MitoSOX staining followed by flow cytometry. **g-h**. Quantification (**g**) and statistical analysis (**h**) of the relative Rhod-2 AM fluorescence intensity time course in the indicated ESCC cells. The fluorescence intensity at each time point was recorded with an integrated spectrofluorometer at excitation and emission wavelengths of 550 nm and 580 nm, respectively. The relative fluorescence intensity was calculated as a percentage of the baseline fluorescence intensity, which was recorded during the first 60 s (F0). The arrow indicates the time at which Tg was added. The data are presented as the means and SDs (*n* = 3). Statistical significance was determined by one-way ANOVA. **P < 0.01. i. The indicated ESCC cells were incubated in the presence or absence of the MCU inhibitor DS16570511 (10 μM), and mitochondrial ROS levels were then measured by MitoSOX staining, followed by flow cytometry

**Incorrect Figure 8**

**Fig. 8:**
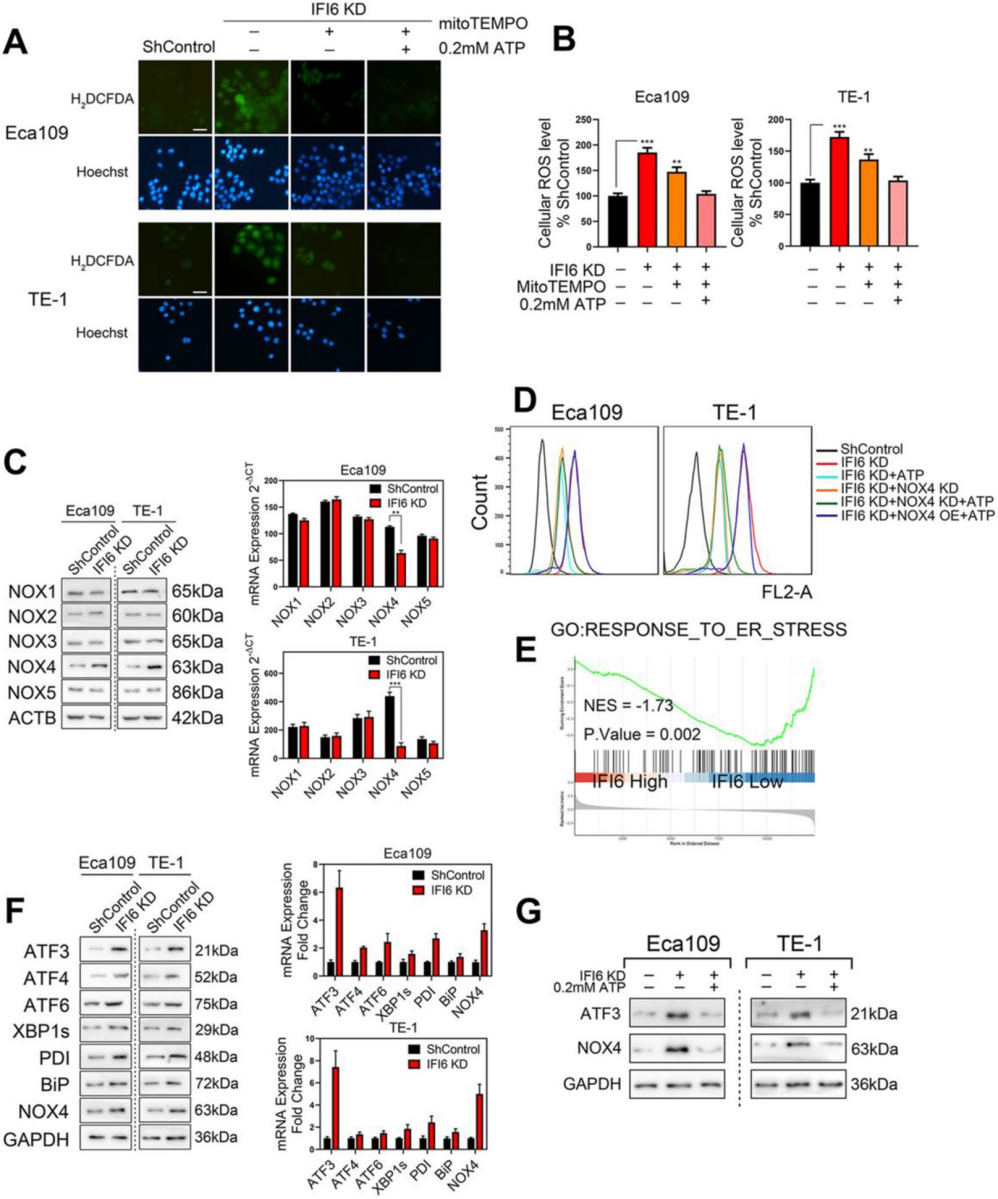
IFI6 silencing elevates ROS levels via the ATF3-NOX4 axis. **a-b**. Representative images (**a**) and statistical quantification (**b**) of ROS production assay results in ESCC cells. The indicated cells were treated with MitoTEMPO (20 μM) or exogenous ATP (0.2 mM), stained with carboxy-H2DCFDA and observed under a fluorescence microscope. H2DCFDA: green, Hoechst: blue. Scale bar: 20 μm. The data are presented as the means and SDs (*n* = 3). Statistical significance was determined by two-tailed Student’s t-test. **P < 0.01, ***P < 0.005. **c**. Immunoblot image (left) and RT-PCR results (right) for a panel of NOX isoforms in ESCC cells with stable IFI6 knockdown. GAPDH was used as the internal control. The data are presented as the means and SDs (*n* = 3). Statistical significance was determined by two-tailed Student’s t-test. **P < 0.01, ***P < 0.005. **d**. The indicated Eca109 and TE-1 cells were treated in the absence or presence of 0.2 mM exogenous ATP, and the cellular ROS level was measured by carboxy-H2DCFDA staining followed by flow cytometry. **e**. ESCC patients in the TCGA database were divided into a high-IFI6 group and a low-IFI6 group according to their IFI6 expression level. GSEA was performed to compare the two groups. NES: normalized enrichment score. **f**. Immunoblot image (left) and RT-PCR results (right) of a series of ER stress markers in ESCC cells with stable IFI6 knockdown. GAPDH was used as the internal control. **g**. The indicated Eca109 and TE-1 cells were treated in the absence or presence of 0.2 mM exogenous ATP. Then, protein lysates were collected and subjected to immunoblotting to assess the expression of IFI6, ATF3 and NOX4. GAPDH was used as the loading control

**Correct Figure 8**

**Fig. 8:**
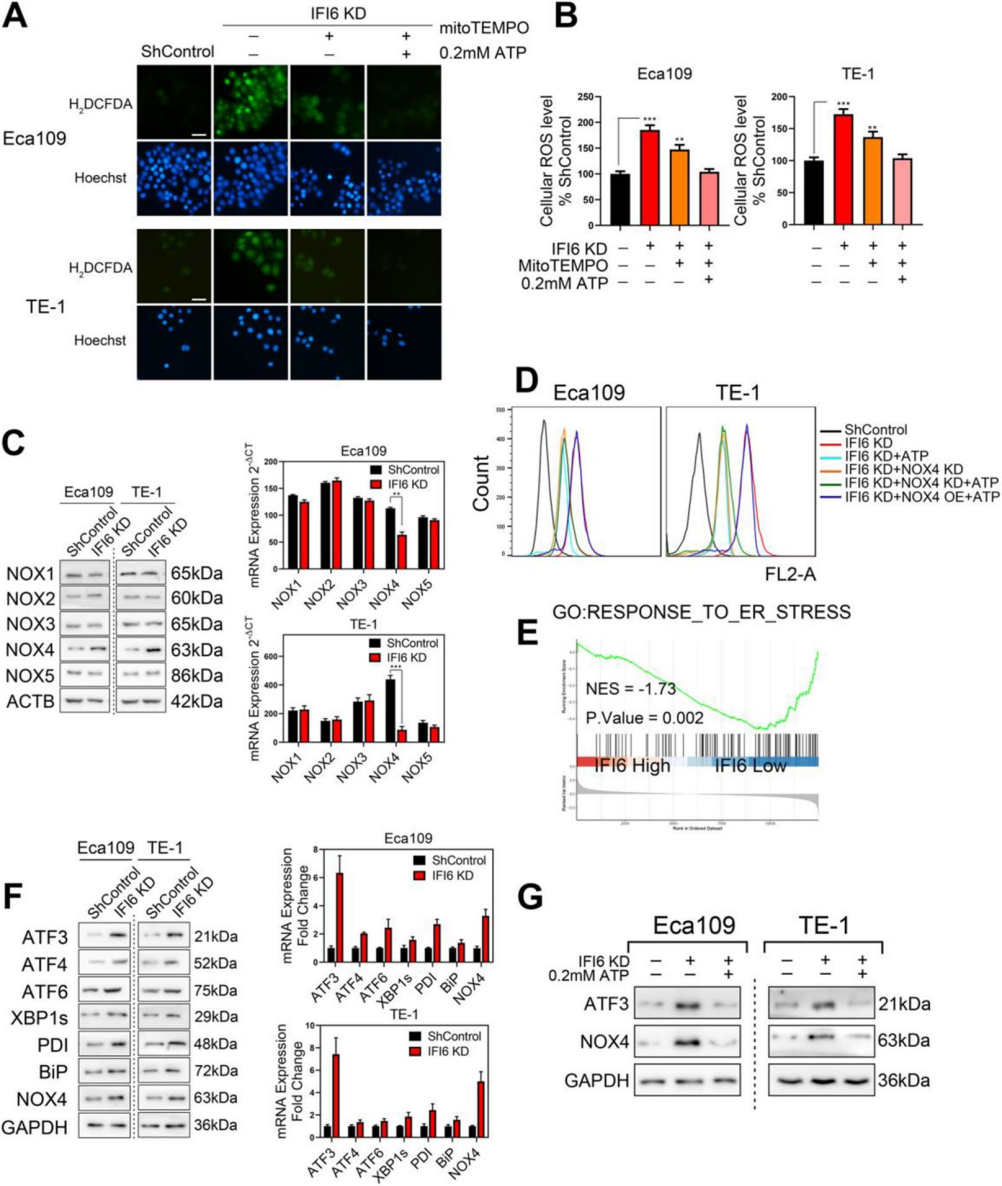
IFI6 silencing elevates ROS levels via the ATF3-NOX4 axis. **a-b**. Representative images (**a**) and statistical quantification (**b**) of ROS production assay results in ESCC cells. The indicated cells were treated with MitoTEMPO (20 μM) or exogenous ATP (0.2 mM), stained with carboxy-H2DCFDA and observed under a fluorescence microscope. H2DCFDA: green, Hoechst: blue. Scale bar: 20 μm. The data are presented as the means and SDs (*n* = 3). Statistical significance was determined by two-tailed Student’s t-test. **P < 0.01, ***P < 0.005. **c**. Immunoblot image (left) and RT-PCR results (right) for a panel of NOX isoforms in ESCC cells with stable IFI6 knockdown. GAPDH was used as the internal control. The data are presented as the means and SDs (*n* = 3). Statistical significance was determined by two-tailed Student’s t-test. **P < 0.01, ***P < 0.005. **d**. The indicated Eca109 and TE-1 cells were treated in the absence or presence of 0.2 mM exogenous ATP, and the cellular ROS level was measured by carboxy-H2DCFDA staining followed by flow cytometry. **e**. ESCC patients in the TCGA database were divided into a high-IFI6 group and a low-IFI6 group according to their IFI6 expression level. GSEA was performed to compare the two groups. NES: normalized enrichment score. **f**. Immunoblot image (left) and RT-PCR results (right) of a series of ER stress markers in ESCC cells with stable IFI6 knockdown. GAPDH was used as the internal control. **g**. The indicated Eca109 and TE-1 cells were treated in the absence or presence of 0.2 mM exogenous ATP. Then, protein lysates were collected and subjected to immunoblotting to assess the expression of IFI6, ATF3 and NOX4. GAPDH was used as the loading control

**Incorrect Figure 10**

**Fig. 10:**
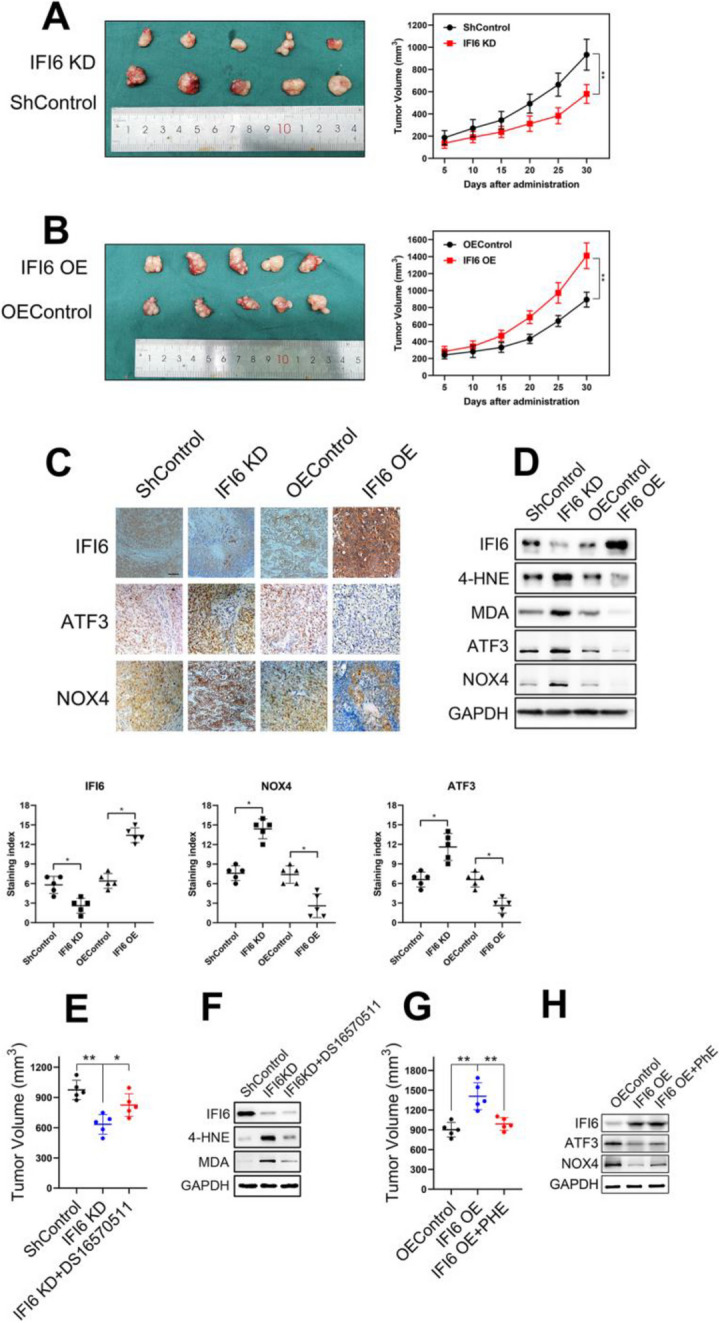
IFI6 promotes the growth of xenograft tumors from Eca109 cells. **a-b**. Representative image of fresh tumor tissues and tumor size quantification results in the xenograft model. Athymic nude mice were inoculated with the indicated Eca109 cells, and the tumor volumes (mm^3^ ) were calculated every 5 days during implantation. The data are presented as the means and SDs (*n* = 5). Statistical significance was determined by two-tailed Student’s t-test. **P < 0.01. **c**. Representative image (upper) and quantitative results (bottom) of the IHC measuring the abundance of IFI6, ATF3 and NOX4 in indicated tumor tissues derived from xenograft model. Magnification: 40×. Scale bar: 50 μm. Statistical significance was determined by Mann-Whitney test. *P < 0.5. d. Representative immunoblot showing IFI6, ATF3, NOX4 and ROS markers in indicated tumor tissues derived from xenograft model. GAPDH was used as the loading control. e. Tumor size quantification results in the xenograft model. The data are presented as the means and SDs (*n* = 5). Statistical significance was determined by two-tailed Student’s t-test. **P < 0.01. **f**. Representative immunoblot showing IFI6 and ROS markers in indicated tumor tissues derived from xenograft model. GAPDH was used as the loading control. **g**. Tumor size quantification results in the xenograft model. The data are presented as the means and SDs (*n* = 5). Statistical significance was determined by two-tailed Student’s t-test. **P < 0.01. h. Representative immunoblot showing IFI6, ATF3 and NOX4 in indicated tumor tissues derived from xenograft model. GAPDH was used as the loading control

**Correct Figure 10**

**Fig. 10:**
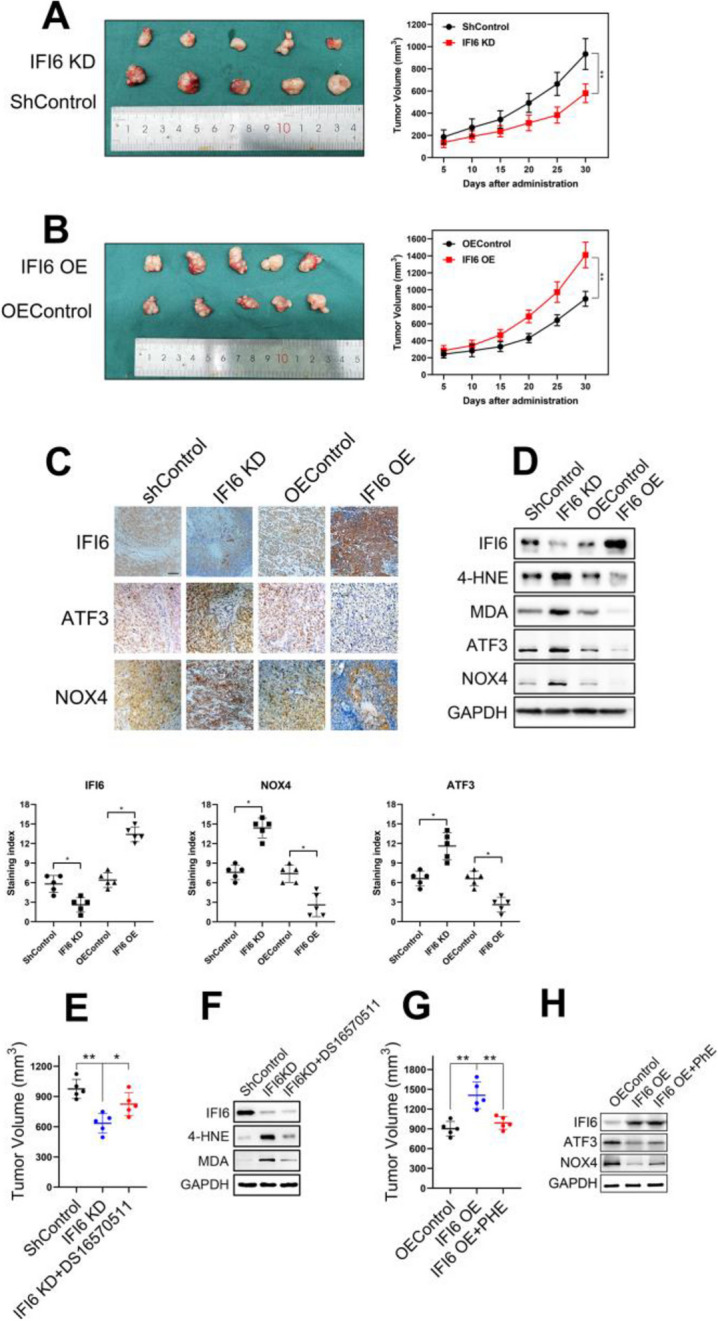
IFI6 promotes the growth of xenograft tumors from Eca109 cells. **a-b**. Representative image of fresh tumor tissues and tumor size quantification results in the xenograft model. Athymic nude mice were inoculated with the indicated Eca109 cells, and the tumor volumes (mm^3^ ) were calculated every 5 days during implantation. The data are presented as the means and SDs (*n* = 5). Statistical significance was determined by two-tailed Student’s t-test. **P < 0.01. **c**. Representative image (upper) and quantitative results (bottom) of the IHC measuring the abundance of IFI6, ATF3 and NOX4 in indicated tumor tissues derived from xenograft model. Magnification: 40×. Scale bar: 50 μm. Statistical significance was determined by Mann-Whitney test. *P < 0.5. d. Representative immunoblot showing IFI6, ATF3, NOX4 and ROS markers in indicated tumor tissues derived from xenograft model. GAPDH was used as the loading control. e. Tumor size quantification results in the xenograft model. The data are presented as the means and SDs (*n* = 5). Statistical significance was determined by two-tailed Student’s t-test. **P < 0.01. **f**. Representative immunoblot showing IFI6 and ROS markers in indicated tumor tissues derived from xenograft model. GAPDH was used as the loading control. **g**. Tumor size quantification results in the xenograft model. The data are presented as the means and SDs (*n* = 5). Statistical significance was determined by two-tailed Student’s t-test. **P < 0.01. h. Representative immunoblot showing IFI6, ATF3 and NOX4 in indicated tumor tissues derived from xenograft model. GAPDH was used as the loading control

## Supplementary Information

** Additional file 1: Table S4.** Primers used for quantitative real-time PCR.
