# Supplementary figures and images for: IFI6 depletion inhibits esophageal squamous cell carcinoma progression through reactive oxygen species accumulation via mitochondrial dysfunction and endoplasmic reticulum stress
Source: J Exp Clin Cancer Res. 2020 Jul 29;39:144. doi: 10.1186/s13046-020-01646-3 (PMC7388476; doi:10.1186/s13046-020-01646-3)

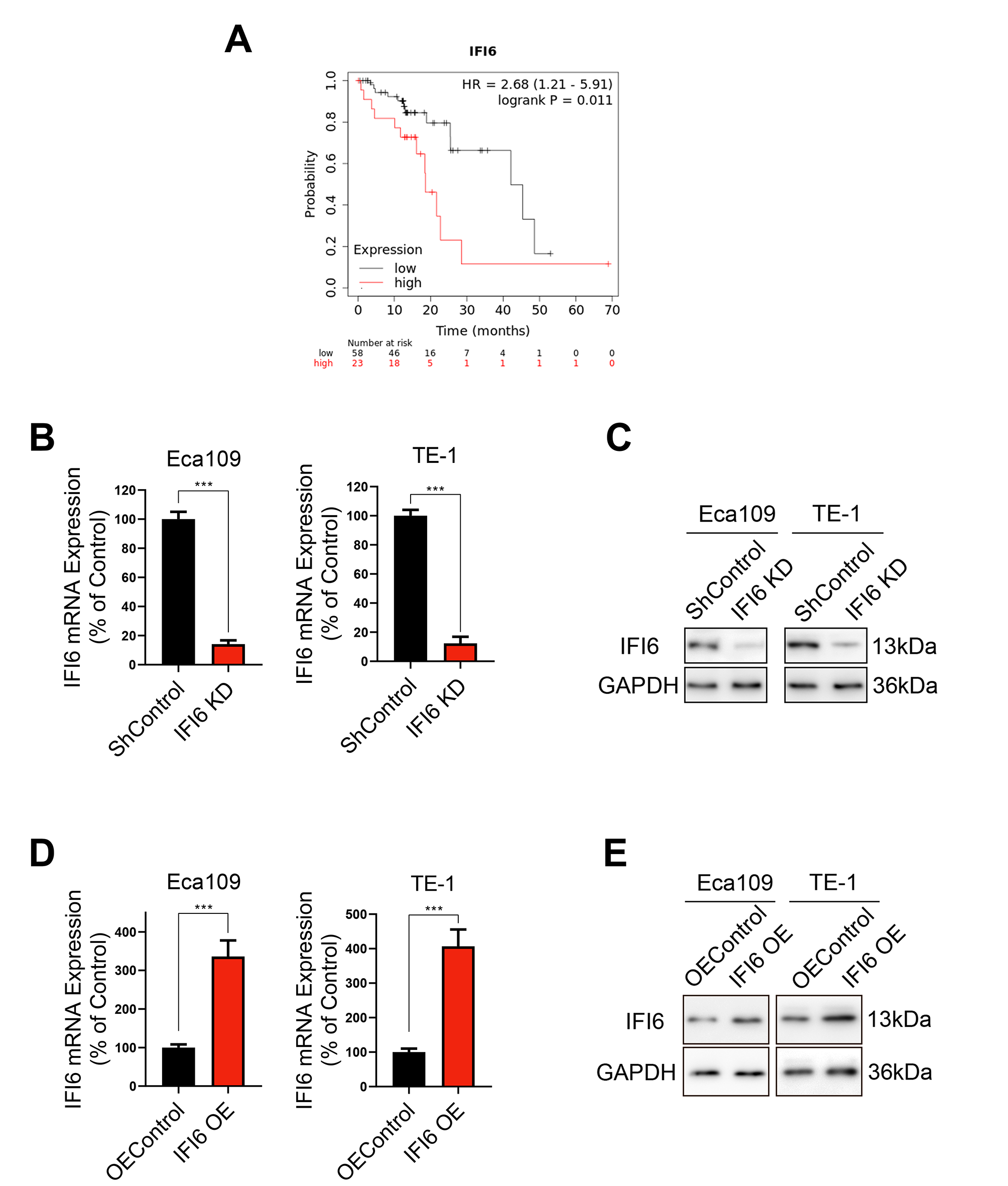

Supplement: Supplementary file 1 — Additional file 1: Figure S1. IFI6 expression in ESCC cells transfected with IFI6 construct or IFI6-ShRNA and its prognostic value in ESCC. A. Plots visualizing the Kaplan-Meier analysis and log-rank test for ESCC patients from the TCGA database separated into groups with high or low expression levels of IFI6. Statistical significance was determined via the log-rank test. B. mRNA levels of IFI6, as measured by qRT-PCR, in two ESCC cell lines after shRNA-mediated depletion of IFI6. Data were normalized to the expression of IFI6 in ShControl cells and are presented as the means and SDs (n=3). Statistical significance was determined by a two-tailed Student’s t-test. ***P<0.005. C. Representative immunoblot showing IFI6 protein levels in Eca109 and TE-1 cells after shRNA-mediated depletion of IFI6. GAPDH was used as the loading control. D-E. Eca109 or TE-1 cells were transfected with IFI6 or empty vector and selected in medium containing G418. qRT-PCR (D) and immunoblotting (E) were performed to validate the overexpression efficiency. GAPDH was used as the loading control. Data were normalized to the expression of IFI6 in OEControl cells and are presented as the means and SDs (n=3). Statistical significance was determined by a two-tailed Student’s t-test. ***P<0.005. [file 13046_2020_1646_MOESM1_ESM.tif]

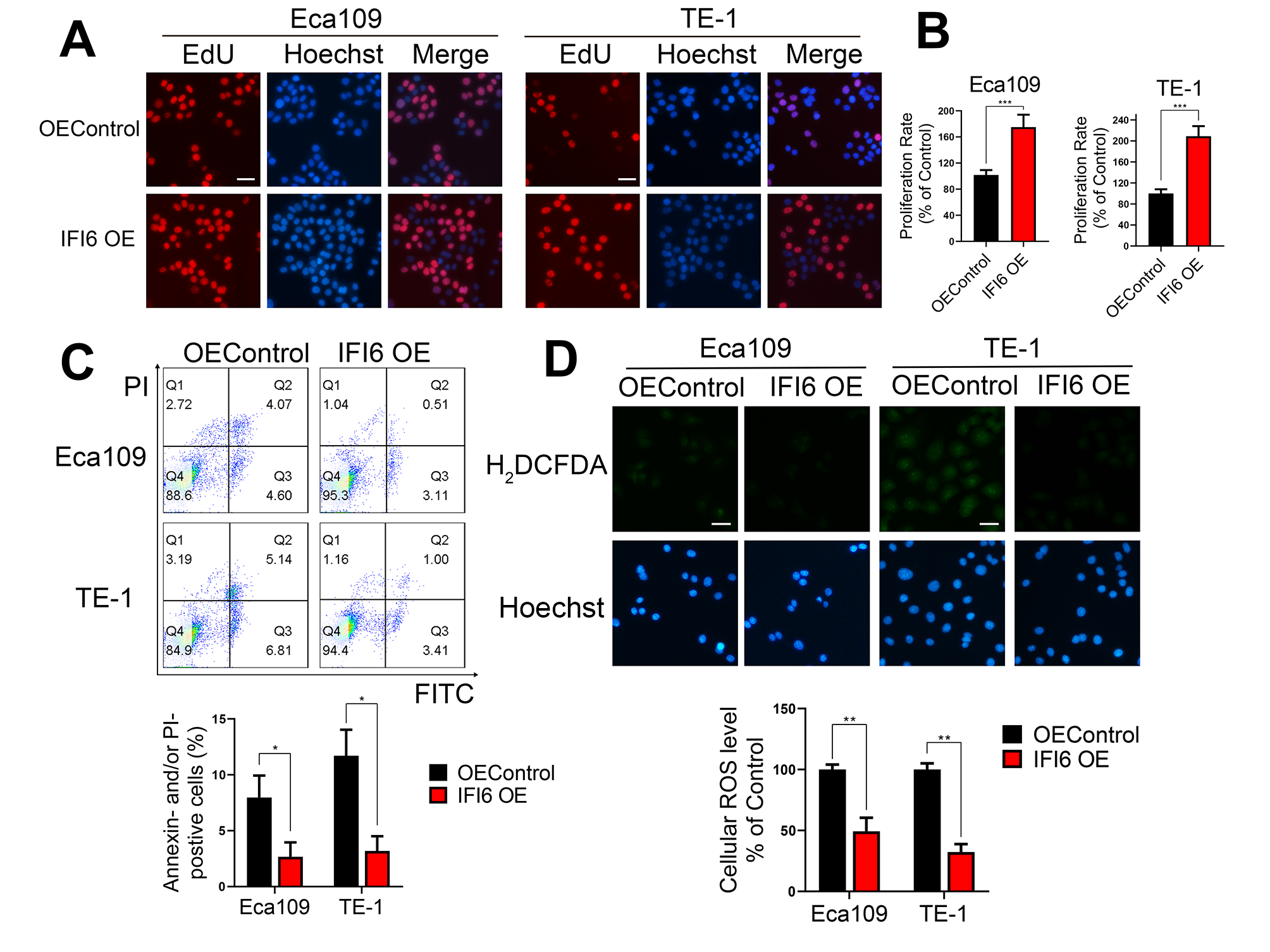

Supplement: Supplementary file 2 — Additional file 2: Figure S2. IFI6 overexpression promotes cell proliferation, inhibits apoptosis and ameliorates oxidative stress in ESCC. A-B. Representative images (A) and statistical quantification (B) of EdU staining in ESCC cell lines transfected with IFI6-plasmic (IFI6OE) or empty vector (OEControl). EdU: red, Hoechst 33342: blue. The data are presented as the means and SDs (n=3). Scale bar: 20 μm. Statistical significance was determined by two-tailed Student’s t-test. ***P<0.005. C. Representative images (upper) and statistical quantification (lower) of apoptotic and necrotic cell populations in ESCC cell lines, as determined by Annexin-V FITC/PI staining and flow cytometry. Cells with a FITC- and PI- signature were considered viable. Cells with a FITC+ and PI- or a FITC+ and PI+ signature were considered nonviable. The data are presented as the means and SDs (n=3). Statistical significance was determined by two-tailed Student’s t-test. **P<0.01. D. Representative images (upper) and statistical quantification (lower) of ROS production assay results in ESCC cells. The indicated cells were stained with carboxy-H2DCFDA and observed under a fluorescence microscope. H2DCFDA: green, Hoechst 33342: blue. Scale bar: 20 μm. The data are presented as the means and SDs (n=3). Statistical significance was determined by two-tailed Student’s t-test. **P<0.01. [file 13046_2020_1646_MOESM2_ESM.tif]

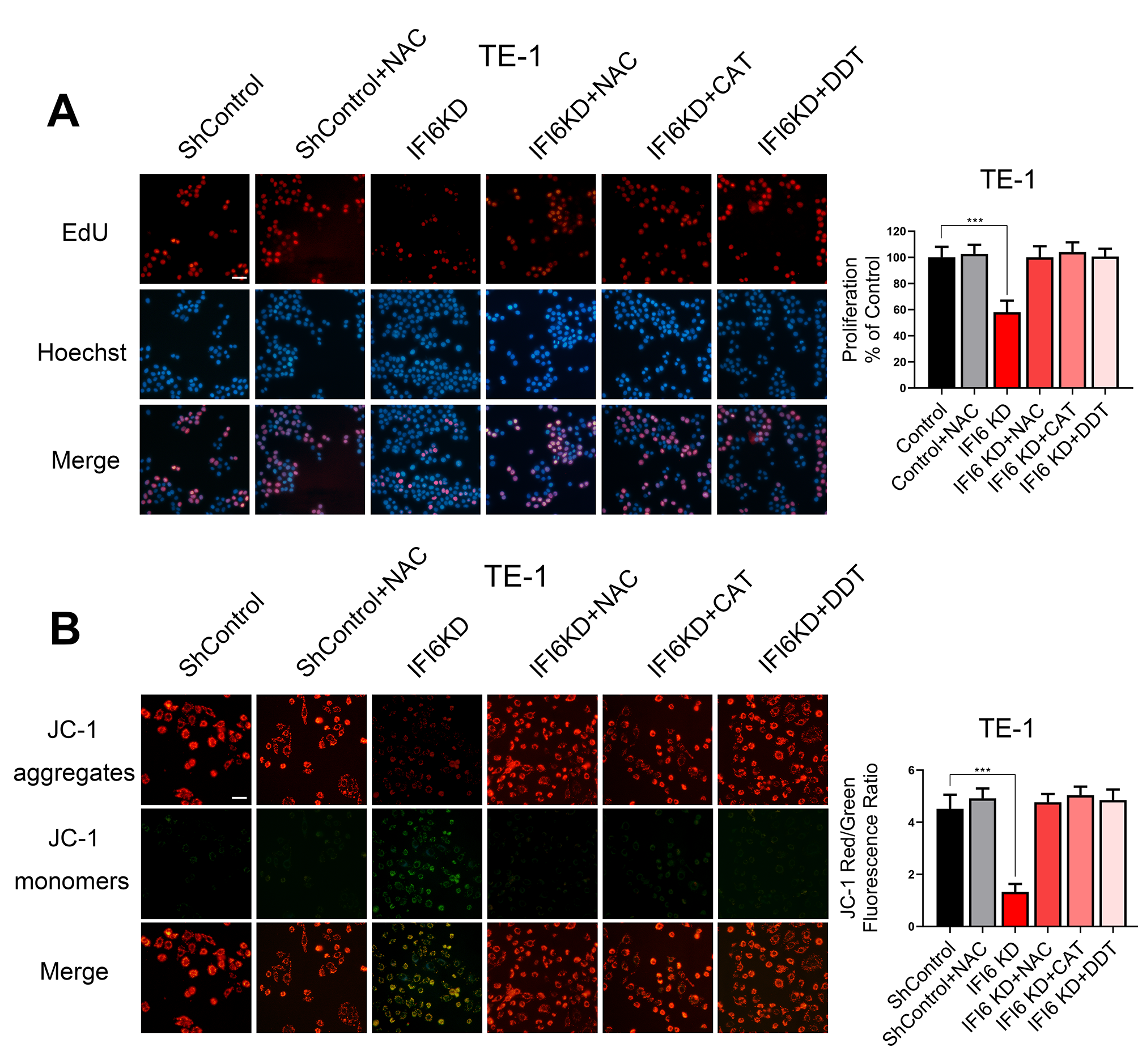

Supplement: Supplementary file 3 — Additional file 3: Figure S3. ROS accumulation is responsible for the IFI6 silencing-induced reduction in cell viability. A. Representative images (left) and statistical quantification (right) of EdU staining in the indicated TE-1 cells preincubated with different ROS inhibitors. EdU: red, Hoechst 33342: blue. Scale bar: 20 μm. The data are presented as the means and SDs (n=3). Statistical significance was determined by one-way ANOVA. ***P<0.005. B. Representative images (left) and statistical quantification (right) of the apoptosis assay results in TE-1 cells, as indicated by the mitochondrial membrane potential. The indicated cells were stained with JC-1 after preincubation with different ROS inhibitors. Cells stained with JC-1 are visible as either green (J-monomers) or red (J-aggregates) fluorescence. The apoptosis rate was calculated as the ratio of JC-1 aggregates to JC-1 monomers. Scale bar: 20 μm. The data are presented as the means and SDs (n=3). Statistical significance was determined by one-way ANOVA. ***P<0.005. [file 13046_2020_1646_MOESM3_ESM.tif]

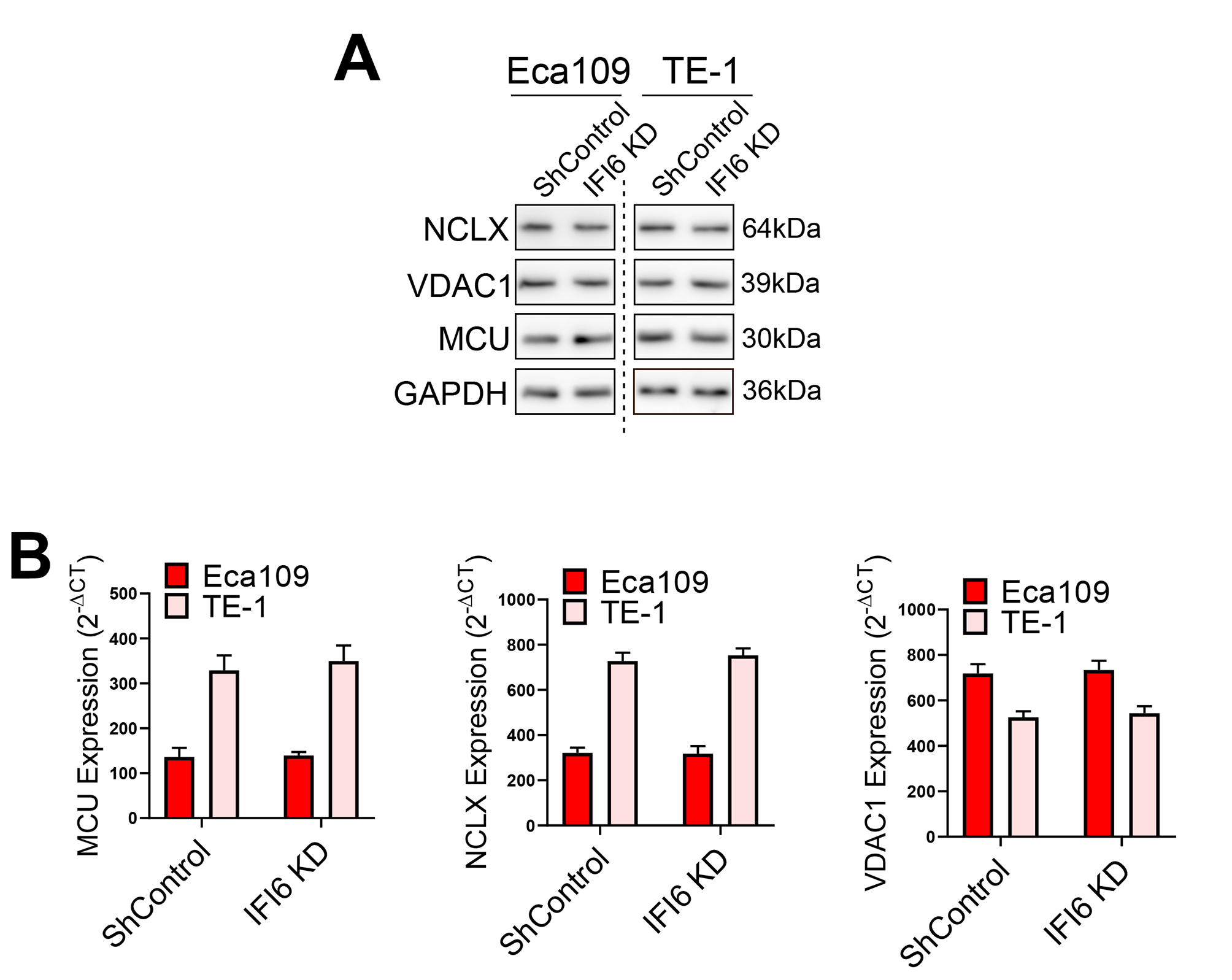

Supplement: Supplementary file 4 — Additional file 4: Figure S4. The expression level of IFI6 does not affect the expression of individual respiratory complexes. A. Immunoblot of NCLX, VDAC1, MCU and GAPDH expression in ESCC cells with stable IFI6 knockdown. B. mRNA levels of NCLX, VDAC1 and MCU in the indicated ESCC cells as measured via qRT-PCR. The data are presented as the means and SDs (n=3). [file 13046_2020_1646_MOESM4_ESM.tif]

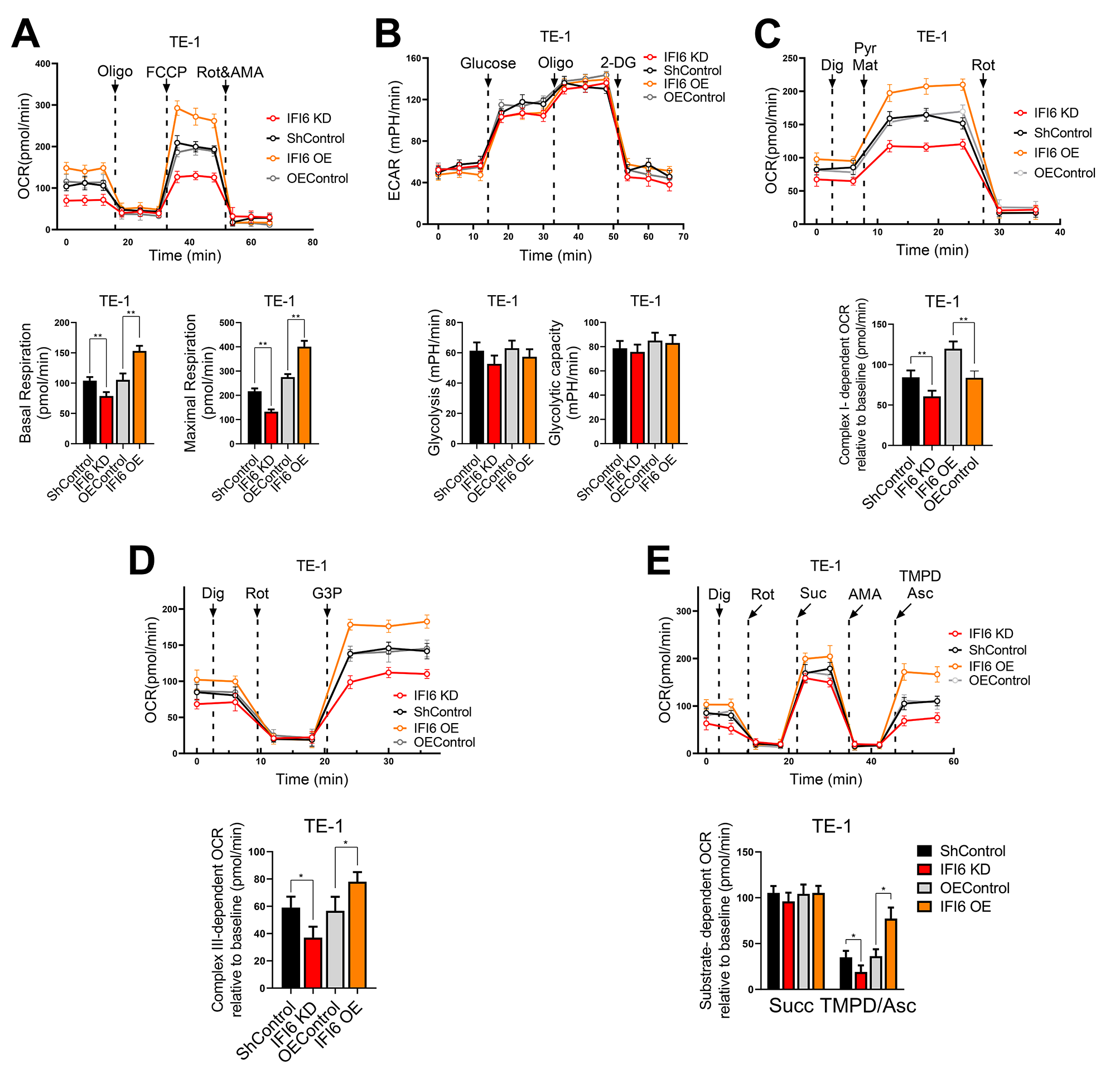

Supplement: Supplementary file 5 — Additional file 5: Figure S5. IFI6 modulates mitochondrial ATP production and the oxidative phosphorylation efficiency. A. Representative plots (upper) and quantitative results (bottom) of the cellular OCR, basal and maximal respiration rates in the different groups. The indicated ESCC cells were subjected to extracellular flux analysis in the Seahorse XF instrument. The arrows and dotted lines indicate the addition of Oligo (oligomycin) (1 μM), FCCP (Carbonyl cyanide 4-(trifluoromethoxy) phenylhydrazone) (0.5 μM) and Rot&AMA (Rotenone and Antimycin A) (0.5 μM each). The data are presented as the means and SDs (n=3). Statistical significance was determined by two-tailed Student’s t-test. **P<0.01. B. Representative plots (upper) and quantitative results (bottom) of the real-time ECAR, glycolysis and glycolytic capacity assays in the indicated ESCC cells. The ECAR was determined following sequential addition of glucose (10 mM), oligomycin (1 μM) and 2-DG (100 mM). Glycolysis was measured by subtracting the ECAR after glucose addition from the ECAR before glucose addition. The glycolytic capacity was calculated by subtracting the ECAR after oligomycin treatment from the ECAR before glucose addition. The data are presented as the means and SDs (n=3). Statistical significance was determined by a two-tailed Student’s t-test. C. Representative plots (upper) and quantitative results (bottom) of the complex I-dependent OCR in the different groups. Pyruvate (Pyr) (5 mM) and malate (Mat) (5 mM) were added to digitonin (Dig)-permeabilized cells, and the OCR was monitored. The data are presented as the means and SDs (n=3). Statistical significance was determined by two-tailed Student’s t-test. **P<0.01. D. Representative plots (upper) and quantitative results (bottom) of the complex III-dependent OCR in the different groups. Rotenone was added to digitonin-permeabilized cells to inhibit complex I, after which G3P (5 mM) was added, and the OCR was monitored. The data are present [file 13046_2020_1646_MOESM5_ESM.tif]

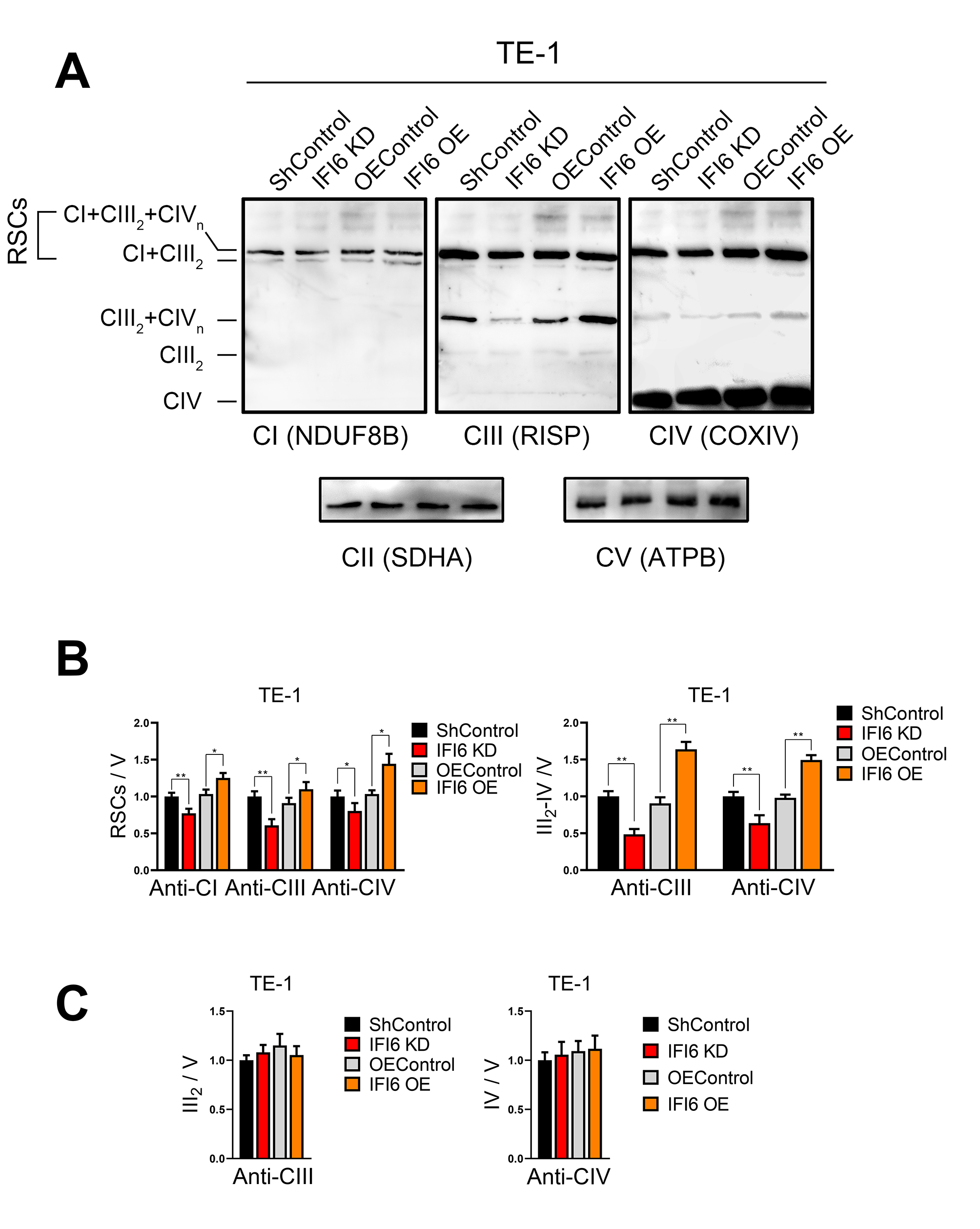

Supplement: Supplementary file 6 — Additional file 6: Figure S6. IFI6 modulates mitochondrial ROS production and OXPHOS efficiency by regulating mitochondrial supercomplex assembly. A. Mitochondrial proteins extracted from the indicated ESCC cells were solubilized with digitonin and subjected to BN-PAGE followed by immunoblotting. Mitochondrial supercomplexes were first visualized by incubation with antibodies against complex I (CI, NDUFB8), and the membrane was then stripped and reprobed with antibodies against complex III (CIII, RISP). The membrane was then sequentially probed with antibodies against complex IV (CIV, COXIV), complex II (CII, SDHA) and complex V (CV, ATPB). B-C. Quantitative results for experiments shown in A. Data are presented as mean and SD (n=3). Statistical significance was determined by two-tailed student’s t-test. **P<0.01. [file 13046_2020_1646_MOESM6_ESM.tif]

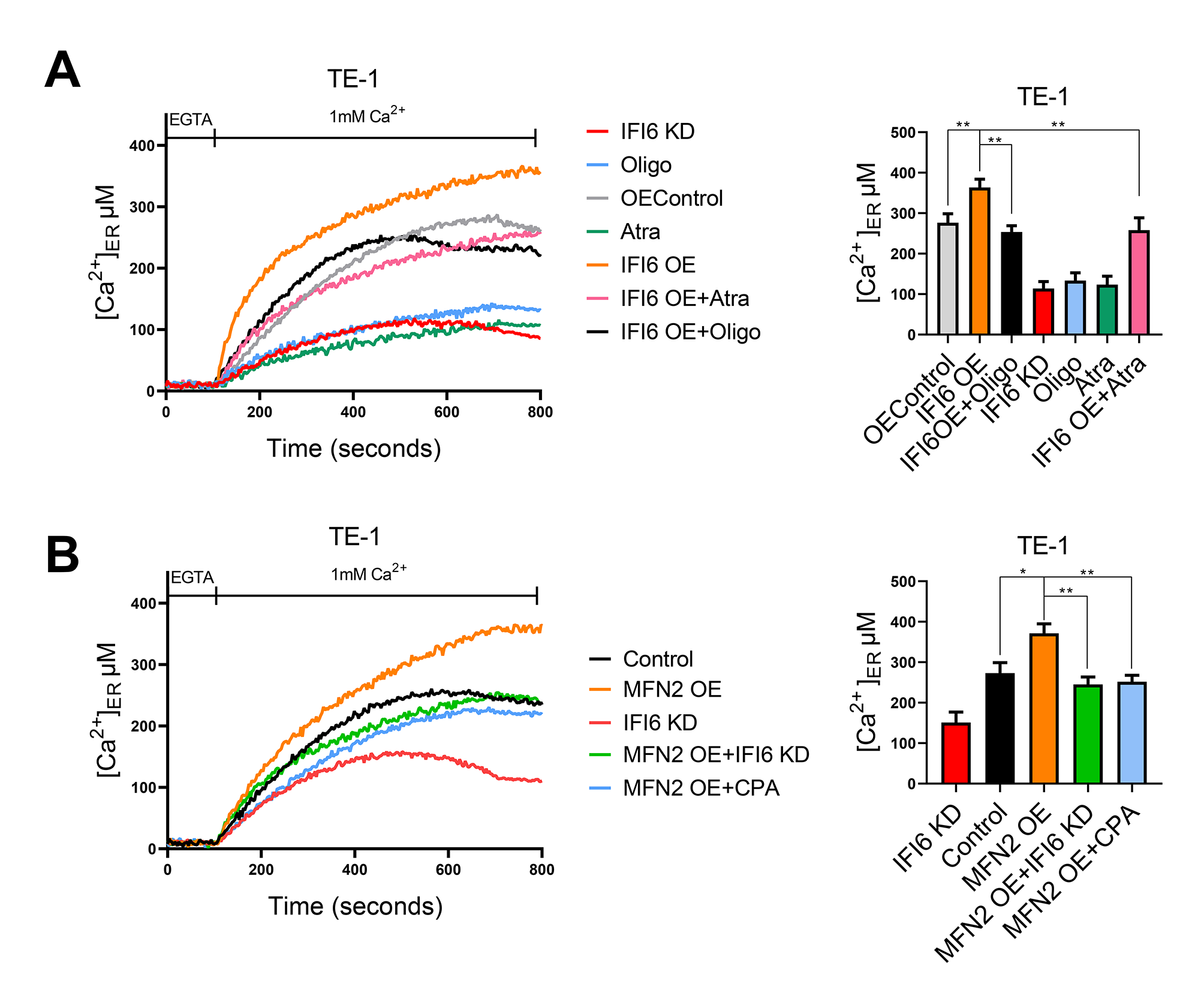

Supplement: Supplementary file 7 — Additional file 7: Figure S7. IFI6 modulates mitochondrial ROS production and OXPHOS efficiency by regulating mitochondrial supercomplex assembly. A. Representation (left) and statistical analysis (right) of the Endoplasmic reticulum Ca2+ in the indicated ESCC cells. Endoplasmic reticulum-targeted aequorin was exploited to monitor dynamic changes in free Ca2+ concentration in ER. The fluorescence intensity at each time point was recorded with an integrated spectrofluoromete. The data are presented as the means and SDs (n=3). Statistical significance was determined by two-tailed Student’s t-test. **P<0.01. B. Representation (left) and statistical analysis (right) of the Endoplasmic reticulum Ca2+ in the indicated ESCC cells. Endoplasmic reticulum-targeted aequorin was exploited to monitor dynamic changes in free Ca2+ concentration in ER. The fluorescence intensity at each time point was recorded with an integrated spectrofluoromete. The data are presented as the means and SDs (n=3). Statistical significance was determined by two-tailed Student’s t-test. **P<0.01. [file 13046_2020_1646_MOESM7_ESM.tif]
