# Supplementary material for: IFI6 depletion inhibits esophageal squamous cell carcinoma progression through reactive oxygen species accumulation via mitochondrial dysfunction and endoplasmic reticulum stress
Source: J Exp Clin Cancer Res. 2020 Jul 29;39:144. doi: 10.1186/s13046-020-01646-3 (PMC7388476; doi:10.1186/s13046-020-01646-3)
Supplement: Supplementary file 8 — Additional file 8: Table S1. Association between IFI6 protein levels and several clinical characteristics in 83 cases of ESCC in the immunohistochemistry cohort. [file 13046_2020_1646_MOESM8_ESM.docx]

**Supplementary Table S1.** Association between IFI6 protein levels and several clinical characteristics in 83 cases of ESCC in the immunohistochemistry cohort.

| **Demographic and Clinical Parameters** | **IFI6 Expression** | | **P-value** |
| --- | --- | --- | --- |
|  | **Low** | **High** |  |
| Sex |  |  |  |
| Male | 22 | 32 | 0.1667 |
| Female | 17 | 12 |  |
| Age |  |  |  |
| ≤60 | 13 | 15 | 0.9999 |
| >60 | 26 | 29 |  |
| Differentiation |  |  |  |
| G1 | 21 | 12 | **0.0211** |
| G2 | 13 | 17 |  |
| G3 | 5 | 15 |  |
| T stage |  |  |  |
| T1 | 15 | 8 | **0.0141** |
| T2 | 16 | 14 |  |
| T3/T4 | 8 | 22 |  |
| N stage |  |  |  |
| N0 | 19 | 11 | 0.0805 |
| N1 | 11 | 18 |  |
| N2/3 | 9 | 15 |  |
| TNM Stage |  |  |  |
| I | 18 | 8 | **0.0189** |
| II | 13 | 19 |  |
| III/IV | 8 | 17 |  |

Statistical analysis was performed using Chi-squared test. P-values<0.05 were considered significant.
