# Supplementary material for: IFI6 depletion inhibits esophageal squamous cell carcinoma progression through reactive oxygen species accumulation via mitochondrial dysfunction and endoplasmic reticulum stress
Source: J Exp Clin Cancer Res. 2020 Jul 29;39:144. doi: 10.1186/s13046-020-01646-3 (PMC7388476; doi:10.1186/s13046-020-01646-3)
Supplement: Supplementary file 9 — Additional file 9: Table S2. Univariate and Multivariate Cox regression analysis for overall survival in 83 ESCC patients from immunohistochemistry cohort. [file 13046_2020_1646_MOESM9_ESM.docx]

**Supplementary Table S2**. Univariate and Multivariate Cox regression analysis for overall survival in 83 ESCC patients from immunohistochemistry cohort.

| **Variables** | **Overall survival** | | | | |
| --- | --- | --- | --- | --- | --- |
|  | **Univariate analysis Multivariate analysis** | | | | |
|  | **HR (95%CI)** | | **P-value** | **HR (95%CI)** | **P-value** |
| T classification |  |  | |  |  |
| T1-T2; T3-T4 | 2.643(1.529-3.927) | | **0.001** | 1.894(1.103-3.442) | **0.022** |
| G classification |  | |  |  |  |
| G1; G2-G3 | 1.902(1.351-2.892) | | **0.003** | 1.143 (0.846-1.695) | 0.511 |
| TNM stage |  | |  |  |  |
| Ⅰ-Ⅱ;Ⅲ-Ⅳ | 2.131(1.246-3.072) | | **0.001** | 2.216(1.127-4.322) | **0.023** |
| IFI6 level |  | |  |  |  |
| High;low | 2.491(1.806-4.145) | | **<0.001** | 2.264(1.395-3.406) | **0.001** |

P-values<0.05 were considered significant.
