# Supplementary material for: IFI6 depletion inhibits esophageal squamous cell carcinoma progression through reactive oxygen species accumulation via mitochondrial dysfunction and endoplasmic reticulum stress
Source: J Exp Clin Cancer Res. 2020 Jul 29;39:144. doi: 10.1186/s13046-020-01646-3 (PMC7388476; doi:10.1186/s13046-020-01646-3)
Supplement: Supplementary file 10 — Additional file 10: Table S3. Clinicopathological features of ESCC patients in the qRT-PCR cohort. [file 13046_2020_1646_MOESM10_ESM.docx]

**Supplementary Table S3.** Clinicopathological features of ESCC patients in the qRT-PCR cohort.

|  | **n = 23**  **n (%)** |
| --- | --- |
| **Age**  Median (range) | 63 (53-77) |
| **Sex**  Male  Female | 18 (78.3)  5 (21.7) |
| **Tumor Location**  Upper  Middle  Lower | 5 (21.7)  8 (34.8)  10 (43.5) |
| **pT**  1  2  3  4 | 8 (34.5)  12 (52.2)  3 (13.1)  0 (0) |
| **pN**  0  1  2  3 | 10 (43.5)  7 (30.4)  5 (21.7)  1 (4.3) |
| **pM**  0  1 | 23 (100)  0 (40) |
| **TNM Stage**  I  II  III  IV | 7 (30.4)  10 (43.5)  4 (17.4)  2 (8.7) |
