# Supplementary material for: IFI6 depletion inhibits esophageal squamous cell carcinoma progression through reactive oxygen species accumulation via mitochondrial dysfunction and endoplasmic reticulum stress
Source: J Exp Clin Cancer Res. 2020 Jul 29;39:144. doi: 10.1186/s13046-020-01646-3 (PMC7388476; doi:10.1186/s13046-020-01646-3)
Supplement: Supplementary file 11 — Additional file 11: Table S4. Primers used for quantitative real-time PCR. [file 13046_2020_1646_MOESM11_ESM.docx]

**Supplementary Table S4.** Primers used for quantitative real-time PCR.

| **Primer** | **Sequence (From 5′ to 3′)** |
| --- | --- |
| IFI6-plus  IFI6-minus | CTCTTCACTTGCAGTGGGGT  TGCTGGCTACTCCTCATCCT |
| GAPDH-plus  GAPDH-minus | GAAAGCCTGCCGGTGACTAA  TTCCCGTTCTCAGCCTTGAC |
| VDAC1-plus  VDAC1-minus  NCLX-plus  NCLX-minus | CAGTGGTAGACTCGGGGAGA  AGACAACAGAAGAAGGATGAGGTT  GGCACTGAGTGTGCTTTGTG  AGGTAACCAAACAGTGCCCC |
| MCU-plus  MCU-minus  ATF3-plus  ATF3-minus | AGTTGAGAGATGGCGGCCGC  AGGTCCATTTCTGCCTGAGC  TCAGCACCTTGCCCCAAAAT  GGATGGCAAACCTCAGCTCT |
| ATF4-plus  ATF4-minus  ATF6-plus  ATF6-minus  XBP1s-plus  XBP1s-minus  PDI-plus  PDI-minus | GTTTTGGATTGGTGGGGTGC  GTATTTGCCCCTCCCTGCTT  CAGCAGGAACTCAGGGAGTG  AATGTGTCTCCCCTTCTGCG  CTGAGTCCGCAGCAGGTG  TCTGCTATCCTCCAGGCAGT  ACGTGCTGCAGTACTTTGGA  GGTCTTAACTGGCCGCTGAT |
| BiP-plus  BiP-minus  NOX1-plus  NOX1-minus | GAACGTCTGATTGGCGATGC  GAGTCGAGCCACCAACAAGA  GAACTCTTGGGGTAGGTGTGTGTT  CGGCTGCAAAACCCAAGGAT |
| NOX2-plus  NOX2-minus  NOX3-plus  NOX3-minus  NOX4-plus  NOX4-minus  NOX5-plus  NOX5-minus | TGTCAAGTGCCCAAAGGTGT  CCCAACGATGCGGATATGGA  TCGGATTGTTCGAGGCCAAA  CTGCTGCTGGGGTGATTGTA  CAGTCTTTGACCCTCGGTCC  TGATCCTCGGAGGTAAGCCA  CTGAAGGCTGTAGAGGCACC  AGTCGAAGTTGAGGCACTGG |
