# Supplementary material for: IFI6 depletion inhibits esophageal squamous cell carcinoma progression through reactive oxygen species accumulation via mitochondrial dysfunction and endoplasmic reticulum stress
Source: J Exp Clin Cancer Res. 2020 Jul 29;39:144. doi: 10.1186/s13046-020-01646-3 (PMC7388476; doi:10.1186/s13046-020-01646-3)
Supplement: Supplementary file 12 — Additional file 12: Table S5. Oligonucleotides used for silencing the expression of target genes. [file 13046_2020_1646_MOESM12_ESM.docx]

**Supplementary Table S5**. Oligonucleotides used for silencing the expression of target genes.

| **Target Gene** | **Sequence (From 5′ to 3′)** |
| --- | --- |
| **IFI6 #1**  **IFI6 #2**  **ATF3 #1**  **ATF3 #2**  **NOX4 #1** | TTCTTCCTTCTTGGCCTAACT  GCTATTCACAGATGCGAACAT  GTTGTGCTTTCTAGCAAATAT  CTTCATCGGCCCACGTGTATT  GAGCCTCAGCATCTGTTCTTA |
| **NOX4 #2** | CAGAGTTTACCCAGCACAAAT |
