# Supplementary material for: IFI6 depletion inhibits esophageal squamous cell carcinoma progression through reactive oxygen species accumulation via mitochondrial dysfunction and endoplasmic reticulum stress
Source: J Exp Clin Cancer Res. 2020 Jul 29;39:144. doi: 10.1186/s13046-020-01646-3 (PMC7388476; doi:10.1186/s13046-020-01646-3)
Supplement: Supplementary file 13 — Additional file 13: Table S6. The 167 mRNAs predicted to be coexpressed with IFI6 in all four GEO datasets. [file 13046_2020_1646_MOESM13_ESM.docx]

**Supplementary Table S6.** The 167 mRNAs predicted to be coexpressed with IFI6 in all four GEO datasets.

| **Gene Symbol** | **GSE20347** | **GSE23400** | **GSE45670** | **GSE75241** | **Mean** |
| --- | --- | --- | --- | --- | --- |
| **ATF3** | -0.563 | -0.542 | -0.560 | -0.780 | -0.611 |
| **P2RX2** | -0.545 | -0.490 | -0.566 | -0.804 | -0.601 |
| **NOX4** | -0.536 | -0.530 | -0.504 | -0.807 | -0.594 |
| **RPTOR** | -0.701 | -0.435 | -0.525 | -0.679 | -0.585 |
| **BCAS3** | -0.491 | -0.437 | -0.677 | -0.730 | -0.584 |
| **PENK** | -0.529 | -0.504 | -0.527 | -0.774 | -0.583 |
| **TLR2** | -0.596 | -0.554 | -0.529 | -0.619 | -0.575 |
| **UCP3** | -0.480 | -0.488 | -0.591 | -0.735 | -0.574 |
| **BMP2** | -0.561 | -0.521 | -0.458 | -0.749 | -0.572 |
| **GAS2L1** | -0.543 | -0.469 | -0.487 | -0.774 | -0.569 |
| **SOD3** | -0.525 | -0.515 | -0.479 | -0.748 | -0.567 |
| **TRH** | -0.556 | -0.484 | -0.449 | -0.778 | -0.567 |
| **CYBA** | -0.536 | -0.501 | -0.480 | -0.741 | -0.564 |
| **CHRNA7** | -0.601 | -0.417 | -0.535 | -0.701 | -0.564 |
| **MARS1** | -0.619 | -0.422 | -0.451 | -0.756 | -0.562 |
| **FADS1** | -0.513 | -0.492 | -0.436 | -0.781 | -0.555 |
| **RRAGA** | -0.441 | -0.399 | -0.590 | -0.783 | -0.553 |
| **GPX7** | -0.463 | -0.467 | -0.472 | -0.811 | -0.553 |
| **FUNDC1** | -0.527 | -0.453 | -0.497 | -0.734 | -0.553 |
| **TBC1D5** | -0.557 | -0.466 | -0.430 | -0.753 | -0.552 |
| **PRDX2** | -0.510 | -0.470 | -0.565 | -0.660 | -0.551 |
| **GPX1** | -0.522 | -0.468 | -0.445 | -0.769 | -0.551 |
| **PCK2** | -0.519 | -0.479 | -0.500 | -0.702 | -0.550 |
| **GBA** | -0.577 | -0.494 | -0.580 | -0.548 | -0.550 |
| **ERP27** | -0.544 | -0.487 | -0.488 | -0.669 | -0.547 |
| **NFE2L1** | -0.528 | -0.411 | -0.501 | -0.745 | -0.546 |
| **GCGR** | -0.473 | -0.463 | -0.467 | -0.774 | -0.544 |
| **EIF2AK3** | -0.508 | -0.545 | -0.515 | -0.606 | -0.543 |
| **PNPT1** | -0.504 | -0.435 | -0.598 | -0.626 | -0.541 |
| **DDIT3** | -0.494 | -0.481 | -0.406 | -0.783 | -0.541 |
| **FSTL1** | -0.461 | -0.496 | -0.561 | -0.626 | -0.536 |
| **NPPA** | -0.517 | -0.475 | -0.416 | -0.732 | -0.535 |
| **MYB** | -0.461 | -0.478 | -0.535 | -0.667 | -0.535 |
| **PYCR1** | -0.431 | -0.415 | -0.592 | -0.695 | -0.533 |
| **PRDX1** | -0.475 | -0.523 | -0.410 | -0.713 | -0.530 |
| **DSC2** | -0.518 | -0.463 | -0.466 | -0.663 | -0.528 |
| **FANCD2** | -0.504 | -0.488 | -0.519 | -0.595 | -0.526 |
| **MSRB3** | -0.613 | -0.502 | -0.466 | -0.517 | -0.524 |
| **SELENOS** | -0.451 | -0.469 | -0.432 | -0.739 | -0.523 |
| **SOD2** | -0.483 | -0.430 | -0.477 | -0.701 | -0.523 |
| **NFE2L2** | -0.525 | -0.419 | -0.499 | -0.644 | -0.522 |
| **GNPAT** | -0.479 | -0.477 | -0.407 | -0.718 | -0.520 |
| **PDIA2** | -0.464 | -0.455 | -0.424 | -0.725 | -0.517 |
| **GPX5** | -0.592 | -0.396 | -0.412 | -0.668 | -0.517 |
| **SNCA** | -0.506 | -0.409 | -0.466 | -0.682 | -0.516 |
| **STK24** | -0.583 | -0.393 | -0.467 | -0.585 | -0.515 |
| **ATP2A2** | -0.518 | -0.466 | -0.459 | -0.609 | -0.513 |
| **TXNRD1** | -0.447 | -0.447 | -0.429 | -0.729 | -0.513 |
| **PPP1R15A** | -0.521 | -0.393 | -0.48 | -0.654 | -0.512 |
| **TMEM161A** | -0.486 | -0.4 | -0.512 | -0.646 | -0.511 |
| **PARK7** | -0.477 | -0.395 | -0.455 | -0.709 | -0.509 |
| **CTSV** | -0.541 | -0.463 | -0.464 | -0.564 | -0.508 |
| **SIRT2** | -0.514 | -0.434 | -0.474 | -0.61 | -0.508 |
| **BCL2L11** | -0.541 | -0.526 | -0.449 | -0.512 | -0.507 |
| **BMPR2** | -0.598 | -0.358 | -0.479 | -0.589 | -0.506 |
| **PDIA3** | -0.51 | -0.448 | -0.467 | -0.595 | -0.505 |
| **ALB** | -0.63 | -0.441 | -0.426 | -0.519 | -0.504 |
| **TGFB2** | -0.652 | -0.458 | -0.402 | -0.5 | -0.503 |
| **KIAA1324** | -0.666 | -0.396 | -0.401 | -0.545 | -0.502 |
| **PPARG** | -0.547 | -0.383 | -0.544 | -0.53 | -0.501 |
| **ENY2** | 0.469 | 0.458 | 0.438 | 0.635 | 0.500 |
| **DDX18** | 0.593 | 0.389 | 0.425 | 0.597 | 0.501 |
| **IVNS1ABP** | 0.614 | 0.419 | 0.457 | 0.518 | 0.502 |
| **DPP3** | 0.519 | 0.401 | 0.484 | 0.608 | 0.503 |
| **ISG20L2** | 0.506 | 0.429 | 0.490 | 0.587 | 0.503 |
| **NASP** | 0.552 | 0.419 | 0.522 | 0.523 | 0.504 |
| **GTF2E1** | 0.601 | 0.433 | 0.404 | 0.580 | 0.504 |
| **ATP2C1** | 0.584 | 0.408 | 0.456 | 0.572 | 0.505 |
| **CCNA2** | 0.502 | 0.379 | 0.451 | 0.687 | 0.505 |
| **CXCL1** | 0.586 | 0.433 | 0.413 | 0.592 | 0.506 |
| **HJURP** | 0.537 | 0.371 | 0.436 | 0.679 | 0.506 |
| **ASPM** | 0.661 | 0.436 | 0.409 | 0.522 | 0.507 |
| **EFHD2** | 0.551 | 0.397 | 0.458 | 0.622 | 0.507 |
| **TGS1** | 0.605 | 0.466 | 0.447 | 0.514 | 0.508 |
| **PANX1** | 0.535 | 0.381 | 0.412 | 0.704 | 0.508 |
| **LSM5** | 0.545 | 0.460 | 0.408 | 0.623 | 0.509 |
| **SMC2** | 0.582 | 0.365 | 0.454 | 0.638 | 0.510 |
| **COL1A1** | 0.554 | 0.442 | 0.408 | 0.640 | 0.511 |
| **BIRC5** | 0.546 | 0.370 | 0.420 | 0.708 | 0.511 |
| **NF1** | 0.427 | 0.487 | 0.474 | 0.655 | 0.511 |
| **POLA2** | 0.619 | 0.422 | 0.446 | 0.561 | 0.512 |
| **CDC25C** | 0.612 | 0.413 | 0.473 | 0.558 | 0.514 |
| **CDC6** | 0.559 | 0.422 | 0.421 | 0.654 | 0.514 |
| **CXCL11** | 0.549 | 0.494 | 0.430 | 0.585 | 0.515 |
| **ZWINT** | 0.517 | 0.406 | 0.459 | 0.678 | 0.515 |
| **CBX3** | 0.472 | 0.501 | 0.410 | 0.679 | 0.516 |
| **MCM10** | 0.461 | 0.535 | 0.429 | 0.637 | 0.516 |
| **RRP15** | 0.532 | 0.502 | 0.407 | 0.631 | 0.518 |
| **KPNA2** | 0.603 | 0.443 | 0.416 | 0.615 | 0.519 |
| **FGFR2** | 0.682 | 0.397 | 0.510 | 0.508 | 0.524 |
| **FUS** | 0.548 | 0.390 | 0.470 | 0.691 | 0.525 |
| **MTHFD2** | 0.508 | 0.468 | 0.436 | 0.695 | 0.527 |
| **SNX10** | 0.611 | 0.452 | 0.411 | 0.634 | 0.527 |
| **APOL1** | 0.500 | 0.513 | 0.424 | 0.673 | 0.528 |
| **MKI67** | 0.600 | 0.422 | 0.459 | 0.633 | 0.528 |
| **NCAPG** | 0.478 | 0.372 | 0.537 | 0.730 | 0.529 |
| **C1orf112** | 0.542 | 0.402 | 0.620 | 0.562 | 0.531 |
| **RRM2** | 0.657 | 0.398 | 0.468 | 0.611 | 0.533 |
| **DNAJC9** | 0.479 | 0.400 | 0.558 | 0.700 | 0.534 |
| **RPN1** | 0.529 | 0.498 | 0.402 | 0.708 | 0.534 |
| **DTL** | 0.492 | 0.459 | 0.506 | 0.685 | 0.536 |
| **TPX2** | 0.534 | 0.436 | 0.434 | 0.739 | 0.536 |
| **PCLAF** | 0.585 | 0.462 | 0.556 | 0.549 | 0.538 |
| **DAB2** | 0.553 | 0.373 | 0.485 | 0.749 | 0.540 |
| **ADAM10** | 0.559 | 0.401 | 0.462 | 0.738 | 0.540 |
| **CHST11** | 0.587 | 0.417 | 0.428 | 0.732 | 0.541 |
| **EPHA8** | 0.726 | 0.477 | 0.420 | 0.548 | 0.543 |
| **IRS1** | 0.442 | 0.560 | 0.448 | 0.721 | 0.543 |
| **CDK1** | 0.634 | 0.433 | 0.448 | 0.667 | 0.545 |
| **MMP1** | 0.511 | 0.529 | 0.505 | 0.640 | 0.546 |
| **FSCN1** | 0.442 | 0.451 | 0.544 | 0.749 | 0.546 |
| **TGFBI** | 0.523 | 0.508 | 0.404 | 0.752 | 0.547 |
| **SMAD6** | 0.599 | 0.468 | 0.509 | 0.619 | 0.548 |
| **LPCAT1** | 0.460 | 0.571 | 0.488 | 0.680 | 0.550 |
| **CKAP2** | 0.759 | 0.371 | 0.470 | 0.599 | 0.550 |
| **DNMT3B** | 0.462 | 0.488 | 0.576 | 0.698 | 0.556 |
| **ELF4** | 0.618 | 0.515 | 0.421 | 0.675 | 0.557 |
| **HSP90AA1** | 0.414 | 0.575 | 0.483 | 0.759 | 0.558 |
| **ARL6IP1** | 0.559 | 0.546 | 0.423 | 0.712 | 0.560 |
| **CXCL10** | 0.588 | 0.444 | 0.473 | 0.738 | 0.561 |
| **CKS2** | 0.595 | 0.527 | 0.427 | 0.742 | 0.573 |
| **GMNN** | 0.569 | 0.529 | 0.446 | 0.754 | 0.574 |
| **MET** | 0.752 | 0.410 | 0.428 | 0.709 | 0.574 |
| **NADK** | 0.596 | 0.589 | 0.565 | 0.559 | 0.577 |
| **IPO9** | 0.690 | 0.510 | 0.407 | 0.704 | 0.578 |
| **LAMA3** | 0.491 | 0.579 | 0.447 | 0.798 | 0.579 |
| **GTSE1** | 0.684 | 0.418 | 0.432 | 0.782 | 0.579 |
| **HNRNPA2B1** | 0.574 | 0.506 | 0.541 | 0.700 | 0.580 |
| **NUP62** | 0.734 | 0.406 | 0.456 | 0.739 | 0.584 |
| **KIF2C** | 0.626 | 0.553 | 0.538 | 0.634 | 0.588 |
| **STIL** | 0.761 | 0.565 | 0.501 | 0.542 | 0.592 |
| **MICB** | 0.612 | 0.458 | 0.517 | 0.793 | 0.595 |
| **FEN1** | 0.797 | 0.499 | 0.445 | 0.647 | 0.597 |
| **ITGB4** | 0.635 | 0.589 | 0.448 | 0.754 | 0.606 |
| **WARS** | 0.600 | 0.529 | 0.482 | 0.817 | 0.607 |
| **CDC20** | 0.615 | 0.572 | 0.520 | 0.751 | 0.614 |
| **GALNT2** | 0.620 | 0.388 | 0.676 | 0.788 | 0.618 |
| **ARPC1B** | 0.781 | 0.393 | 0.552 | 0.757 | 0.621 |
| **LAMC2** | 0.671 | 0.574 | 0.492 | 0.748 | 0.621 |
| **MMP12** | 0.564 | 0.603 | 0.465 | 0.870 | 0.625 |
| **ITGA6** | 0.486 | 0.629 | 0.543 | 0.854 | 0.628 |
| **PSMB2** | 0.657 | 0.666 | 0.598 | 0.606 | 0.632 |
| **MSN** | 0.572 | 0.632 | 0.454 | 0.874 | 0.633 |
| **TDGF1** | 0.518 | 0.706 | 0.627 | 0.757 | 0.652 |
| **GBP1** | 0.597 | 0.645 | 0.698 | 0.670 | 0.653 |
| **LY6E** | 0.777 | 0.664 | 0.651 | 0.520 | 0.653 |
| **NMI** | 0.715 | 0.742 | 0.614 | 0.589 | 0.665 |
| **MELK** | 0.753 | 0.658 | 0.552 | 0.716 | 0.670 |
| **MYC** | 0.753 | 0.649 | 0.804 | 0.713 | 0.730 |
| **PSMB9** | 0.763 | 0.627 | 0.688 | 0.863 | 0.735 |
| **HERC5** | 0.818 | 0.726 | 0.782 | 0.648 | 0.744 |
| **EGFR** | 0.645 | 0.768 | 0.879 | 0.688 | 0.745 |
| **XAF1** | 0.844 | 0.676 | 0.897 | 0.616 | 0.758 |
| **IFI35** | 0.896 | 0.810 | 0.658 | 0.679 | 0.761 |
| **TAP1** | 0.702 | 0.713 | 0.756 | 0.883 | 0.764 |
| **IRF9** | 0.832 | 0.716 | 0.780 | 0.764 | 0.773 |
| **EIF4G1** | 0.831 | 0.746 | 0.786 | 0.732 | 0.774 |
| **IFIT3** | 0.725 | 0.768 | 0.865 | 0.762 | 0.780 |
| **PARP12** | 0.909 | 0.745 | 0.873 | 0.628 | 0.789 |
| **UBE2L6** | 0.825 | 0.758 | 0.747 | 0.829 | 0.789 |
| **CRLF2** | 0.919 | 0.709 | 0.890 | 0.665 | 0.796 |
| **E2F3** | 0.740 | 0.855 | 0.906 | 0.700 | 0.800 |
| **STAT1** | 0.776 | 0.742 | 0.845 | 0.904 | 0.817 |
| **ADAR** | 0.898 | 0.808 | 0.831 | 0.745 | 0.821 |
| **IFI44L** | 0.962 | 0.899 | 0.918 | 0.558 | 0.834 |
| **DDX60** | 0.876 | 0.734 | 0.930 | 0.798 | 0.835 |
| **IFI44** | 0.895 | 0.864 | 0.904 | 0.712 | 0.844 |
